# Does low-energy sweetener consumption affect energy intake and body weight? A systematic review, including meta-analyses, of the evidence from human and animal studies

**DOI:** 10.1038/ijo.2015.177

**Published:** 2015-11-10

**Authors:** P J Rogers, P S Hogenkamp, C de Graaf, S Higgs, A Lluch, A R Ness, C Penfold, R Perry, P Putz, M R Yeomans, D J Mela

**Affiliations:** 1 School of Experimental Psychology, University of Bristol, Bristol, UK; 2 Department of Neuroscience, Uppsala University, Uppsala, Sweden; 3 Division of Human Nutrition, Wageningen University, Wageningen, the Netherlands; 4 The School of Psychology, University of Birmingham, Birmingham, UK; 5 Danone Research, Centre Daniel Carasso, RD, Palaiseau Cedex, France; 6 National Institute for Health Research Biomedical Research Unit in Nutrition, Diet and Lifestyle at the University Hospitals Bristol NHS Foundation Trust and the University of Bristol and School of Oral and Dental Sciences, University of Bristol, Level 3, University Hospitals Bristol Education Centre, Bristol, UK; 7 European Branch, ILSI Europe a.i.s.b.l., Brussels, Belgium; 8 School of Psychology, University of Sussex, Brighton, UK; 9 Unilever R&D Vlaardingen, Vlaardingen, the Netherlands

## Abstract

By reducing energy density, low-energy sweeteners (LES) might be expected to reduce energy intake (EI) and body weight (BW). To assess the totality of the evidence testing the null hypothesis that LES exposure (versus sugars or unsweetened alternatives) has no effect on EI or BW, we conducted a systematic review of relevant studies in animals and humans consuming LES with *ad libitum* access to food energy. In 62 of 90 animal studies exposure to LES did not affect or decreased BW. Of 28 reporting increased BW, 19 compared LES with glucose exposure using a specific ‘learning' paradigm. Twelve prospective cohort studies in humans reported inconsistent associations between LES use and body mass index (−0.002 kg m^−^^2^ per year, 95% confidence interval (CI) −0.009 to 0.005). Meta-analysis of short-term randomized controlled trials (129 comparisons) showed reduced total EI for LES versus sugar-sweetened food or beverage consumption before an *ad libitum* meal (−94 kcal, 95% CI −122 to −66), with no difference versus water (−2 kcal, 95% CI −30 to 26). This was consistent with EI results from sustained intervention randomized controlled trials (10 comparisons). Meta-analysis of sustained intervention randomized controlled trials (4 weeks to 40 months) showed that consumption of LES versus sugar led to relatively reduced BW (nine comparisons; −1.35 kg, 95% CI –2.28 to −0.42), and a similar relative reduction in BW versus water (three comparisons; −1.24 kg, 95% CI –2.22 to −0.26). Most animal studies did not mimic LES consumption by humans, and reverse causation may influence the results of prospective cohort studies. The preponderance of evidence from all human randomized controlled trials indicates that LES do not increase EI or BW, whether compared with caloric or non-caloric (for example, water) control conditions. Overall, the balance of evidence indicates that use of LES in place of sugar, in children and adults, leads to reduced EI and BW, and possibly also when compared with water.

## Introduction

Low-energy sweeteners (LES), such as acesulfame-K, aspartame, saccharin, stevia and sucralose are consumed throughout the world.^1^ The history of their use has been accompanied by debate and disagreements, not least about their potential nutritional impact. The use of LES to replace or partially replace added sugar in foods and beverages might well be expected to reduce energy intake (EI),^2, 3, 4^ yet over recent years there has been widely reported speculation that consumption of LES might increase the risk of becoming overweight and obese.^5, 6, 7^

Studies covertly manipulating energy density show higher EI after consumption of a reduced-energy food or beverage, but that the degree of energy ‘compensation' is variable.^8^ Crucially, energy compensation is usually lower than the difference in energy content of the comparison foods/beverages, seemingly being lowest of all for liquids.^8^ This suggests that consuming LES in place of sugar-sweetened products should reduce overall EI, and particularly so for consumption of beverages, the most popular vehicles for LES.^1^ The question also arises whether the presence of LES in beverages affects appetite and EI relative to plain water.^1, 9^ Although the imprecise control of short-term energy balance predicts that LES consumption should help reduce EI and therefore reduce risk of overweight and obesity,^3, 10^ it is possible that, as consumed in everyday life, other effects of LES balance or even outweigh the energy dilution effect. For example, a low calorie or ‘diet' label may cause the consumer to eat a larger portion of the product or eat more of accompanying foods in the meal, or eat more later.^11, 12, 13, 14^ More simply, adding sweetness to a product may increase intake owing to increased palatability.^15, 16^ Or, by ‘uncoupling' the relationship between sweetness and energy content, the consumption of LES may undermine the usefulness of sweetness as a cue in the control of energy balance.^5, 17, 18, 19, 20^

The effects of LES consumption on EI and body weight (BW) have been the subject of many studies over the past 30 years; nonetheless there is no clear consensus about this body of evidence. Taken together, a number of narrative reviews^1, 2, 3, 4, 21, 22, 23, 24, 25^ and systematic reviews of some types of studies^26, 27, 28, 29, 30^ lead to the consistent but guarded conclusion that substitution of LES for sugar, especially in beverages, can help reduce EI, but that fully convincing evidence for longer-term beneficial effects is lacking. Our principle aim was, therefore, to bring together the totality of evidence to test the primary (null) hypotheses that LES consumption *per se* or as a replacement for caloric sweeteners in foods or beverages has no effect on EI or BW outcomes in adults or children. We conducted systematic reviews of animal, human observational and human intervention studies providing information on LES consumption and EI and/or BW. We included animal studies particularly because findings from a subset of animal studies appear to suggest that consumption of LES might increase risk of BW gain.^19^ Within the human intervention studies we furthermore sought to test a secondary (null) hypothesis that the effects of consumption of LES beverages on EI and BW do not differ from the effects of consumption of water.

## Materials and methods

A systematic search of the literature was undertaken to identify studies in humans and animals that assessed the effects of consumption of LES on EI and/or BW and/or body mass index (BMI). LES were defined as ingredients contributing negligible energy to products to achieve a sweetness similar in comparison with caloric sweeteners. Thus LES include intense sweeteners and non-metabolized sugars but exclude most sugar alcohols (see Supplementary Information for full definition and examples). We use ‘sugars' to refer to caloric sweeteners, usually sucrose.

**Figure 1:**
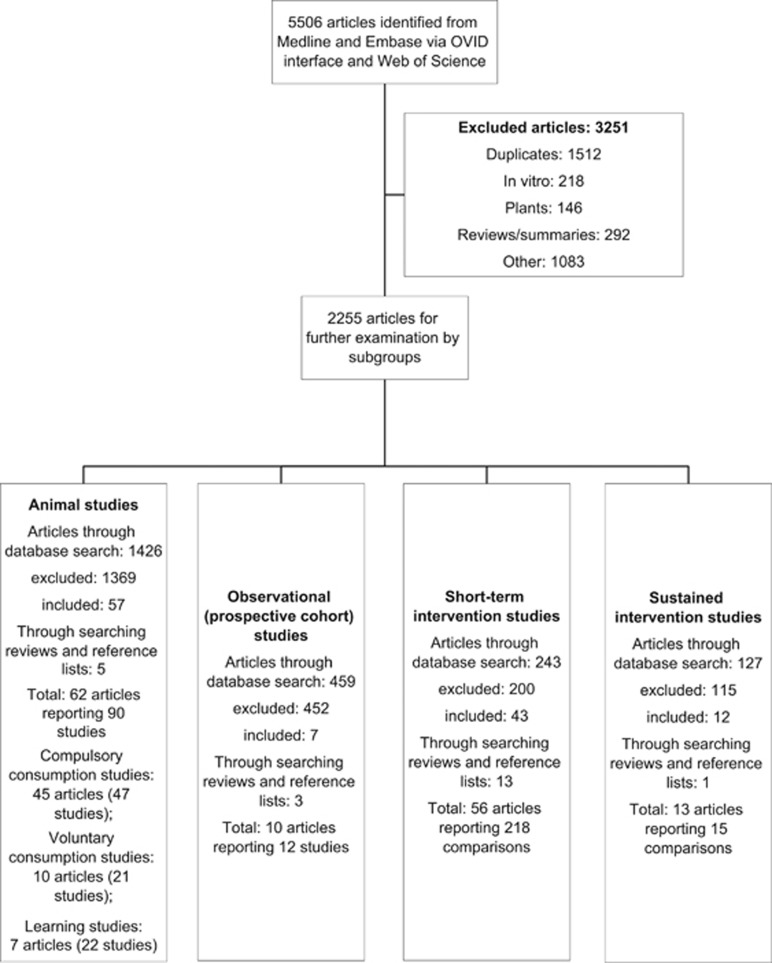
Flow of information through the different phases of the systematic review.

The databases of MEDLINE and EMBASE via OVID interface and Web of Science were searched from their inception to 01 February 2015, using a combination of MeSH terms and key word terms to identify research addressing the relevant combinations of exposures (sweetener types) and outcomes (describing food or EI, anthropometric measures or changes in these). No restrictions were applied regarding language or dates. This search generated 5506 articles, which were processed as summarised in Figure 1. Medline search terms and further details of our methods are provided in the Supplementary Information.

## Animal studies

### Study selection and data extraction

We identified 62 articles reporting a total of 90 eligible studies testing the effects of repeated consumption of LES on BW and/or EI in non-human animals (rats or mice). These studies were divided into three sets based on their primary purpose. The first set investigated effects of long-term compulsory consumption of LES on BW and EI often from primarily a toxicological perspective. The second set included a contrast of voluntary LES consumption usually as a sweetness-control in studies of effects of sugar on behaviour. The third set explored the hypothesis that LES can uncouple the predictive relationship between sweetness and EI (‘learning studies').

### Results

#### Animal studies with compulsory consumption

There were 45 articles^31, 32, 33, 34, 35, 36, 37, 38, 39, 40, 41, 42, 43, 44, 45, 46, 47, 48, 49, 50, 51, 52, 53, 54, 55, 56, 57, 58, 59, 60, 61, 62, 63, 64, 65, 66, 67, 68, 69, 70, 71, 72, 73, 74, 75^ reporting 47 studies examining changes in BW following at least daily compulsory consumption of LES (details in Supplementary Table S1). LES were either added to the animals' only source of food or water, or were orally intubated daily. The studies varied in LES (advantame, 2; aspartame, 4; cyclamate, 3; erythritol, 4; monatin, 2; neohesperidin, 2; oligo-*N*-acetylglucosamine, 1; psicose, 2; saccharin, 8; stevia, 11; sucralose, 2; thaumatin, 1; combinations of LES, 8), species tested (mice, 12; rats, 35), exposure duration (3–104 weeks) and sample size (5–272). Although exact quantification of exposures is often difficult due to the use of water and food vehicles, comparison with human intakes^76^ indicates that most of the studies included dose ranges in excess of the equivalent amounts humans would consume. As a reference example, the upper acceptable daily intake values for saccharin and stevia in the European Union are 5 and 4 mg kg^−1^ per day, respectively,^76, 77^ thus <0.1% of the diet for a 70 kg human eating 500 g dry weight of food per day. The amounts of saccharin (1–7.5% of diet by weight) and stevia (⩾1% of diet by weight, and up to 2 g kg^−1^ per day) tested in most of these animal studies are at least 10 to 100-fold greater.^32, 34, 39, 44, 47, 52, 56, 57^ The majority of studies, including all of the studies with larger sample sizes and/or longer durations of exposure, reported no statistically significant effects of LES on BW (Table 1). Eighteen studies of higher doses (>2% diet) found statistically significant decreases in BW, and in many of these studies this was reported in association with reductions in EI. Only four of these studies reported statistically significant increases in BW. One of these reported increased BW with 1% saccharin in the diet although higher doses led to reduced BW.^32^ However, four further studies also using lower doses (≈1% or less in diet) of saccharin found no statistically significant effects on BW.^33, 34, 56, 66^ One study contrasting effects of adding saccharin, cyclamate, acesulfame-K or aspartame to the drinking water of mice reported significantly increased BW after saccharin and cyclamate.^64^ Another study also reported significantly increased BW (in rats) with a low-dose of cyclamate^40^ but this was not replicated in subsequent studies.^37, 41^ Finally, in rats, addition of saccharin to an oil-enriched diet significantly reduced EI and BW, whereas addition of saccharin to a diet enriched with beef tallow significantly increased EI and BW.^71^

#### Animal studies with voluntary consumption

These studies (10 articles reporting 21 studies^78, 79, 80, 81, 82, 83, 84, 85, 86, 87^) examined effects of LES offered in water or food in addition to the usual diet (details in Supplementary Table S2). The largest group of these studies (*n*=16) offered 0.1–0.2% saccharin solution as well as normal drinking water to rats for 2–3 weeks, with no statistically significant effects on BW, whereas addition of sucrose^82, 84, 87^ or polycose^78, 83^ significantly increased BW in 7 out of 10 studies. The rats consumed ≈30 mg of saccharin solution a day, equivalent to 40 times the human acceptable daily intake. Research that added saccharin to specific types of food found variable effects on BW. In a series of seven studies rats were fed wet mash (lab chow mixed with water) with saccharin added.^86^ In four out of five of these studies in which saccharin was added at the concentration of 0.2%, the rats consumed significantly more food and gained BW significantly faster than did rats given unsweetened mash. These effects were similar to the effects of adding sucrose or fat to the diet.^86^ There was no significant effect reported on BW in two studies in which a higher concentration of saccharin (0.5%) was added to the additional food or when a saccharin solution was offered as well as drinking water.^86^ In a recent study, rats were given access to a diet supplement (yogurt) that had either sucrose, saccharin or aspartame added.^80^ A group receiving an unsweetened supplement was not included. Rats exposed to sucrose consumed less chow and gained less BW than did those exposed to the LES.

#### Learning studies

We identified seven articles,^19, 88, 89, 90, 91, 92, 93^ reporting 22 relevant studies, in which rats were exposed repeatedly on different days to two forms of a diet supplement: a sweetened version (either added glucose or LES) and a plain unsweetened version (details in Supplementary Table S3). The hypothesis under test was that, unlike rats exposed to a diet sweetened with glucose, rats exposed to a diet sweetened with LES will not learn that sweetness is associated with additional energy and as a result will have impaired ability to control intake of sweet food (specifically intake of their moderately sweet maintenance diet). Outcomes for all eligible studies were summarised in terms of whether there were statistically significant differences in BW and/or EI between LES and comparison conditions.

In 14 studies there was an increase in BW gain observed for the rats that had access to a LES-sweetened diet supplement, compared with those rats that had access to a glucose-sweetened diet supplement.^19, 88, 89, 90, 91, 92, 93^ In three studies, there was no effect of the experimental manipulation on BW gain over time.^91, 92, 93^ In four studies the effect of LES exposure was found to be moderated by another factor such as sex, previous exposure to a high-fat diet, hormonal status or genetic proneness to obesity.^92, 93^ In one study there was a significant day by sweetener interaction, but *post hoc* testing did not reveal a significant effect of sweetener exposure on any individual day.^91^

In one of the studies, a group was included that received a supplement (yogurt) sweetened with glucose on 3 days per week but no unsweetened supplement.^89^ This control group did not differ in BW gain from the group that received glucose-sweetened yogurt on some days and unsweetened yogurt on other days.

### Commentary

A large majority of studies of compulsory and voluntary long-term consumption of LES by rodents found that LES did not increase BW. Only under certain conditions, perhaps where LES increased the palatability of specific diets, was increased BW observed. At higher doses of LES most studies found either no effect, or reduced BW.

The results of the learning studies in rats suggest that intermittent exposure to a diet supplement sweetened with LES interweaved with exposure to the same non-sweetened diet can result in an increase in BW gain when compared with exposure to a glucose-sweetened diet. This has been replicated many times and, although there are some moderating factors, the effect appears robust. However, the relevance to usual human eating patterns remains unclear and specific hypotheses generated from these animal studies have yet to be tested directly in controlled studies with humans. The rats in these learning studies have limited dietary experience and, unlike humans who consume LES alongside sugar-containing foods, they do not have experience of consuming similar sweet tastes both with and without energy. Future research could assess the validity of the animal studies for human eating patterns by examining whether people who consume foods with the same taste but a range of energy densities, for example, sometimes eating regular versions and sometimes ‘diet' versions of the same food, are more likely to overeat that food due to uncertainty about the energy delivered by that food. Until such tests have been conducted, continued speculation based on the results of these animal studies about the impact of LES use by people would seem unwarranted.

This review also suggests that the precise nature of the effects of LES on BW observed in these learning studies should be investigated further. In particular, more information is needed on BW gain in rats that receive a glucose-sweetened diet supplement on some days and an unsweetened diet supplement on other days compared with appropriate control groups (for example, rats exposed to only glucose-sweetened or only unsweetened diet supplements). This would determine whether or not exposure to glucose itself, rather than learning about the relationship between sweet taste and energy content, might account for differences in BW gain relative to rats exposed to LES intermittently.

## Observational (prospective cohort) studies in humans

### Study selection and data extraction

Studies of over 500 participants that reported on prospective analyses with more than 1 year of follow-up were included. These studies had to report on LES beverages or LES intake as an exposure and BW or relative adiposity as an outcome. Studies that reported on LES consumption and metabolic syndrome but did not report separate associations for an anthropometric component of metabolic syndrome were excluded. Only one report of the results from each prospective study was included. Where there were several reports of the same study we included either the most detailed report of the results or the report with the longest follow-up. We did not include studies published only as an abstract. Our methods for data extraction are described in the Supplementary Information.

### Results

We identified 10 articles reporting 12 studies^7, 94, 95, 96, 97, 98, 99, 100, 101, 102^ that met the eligibility criteria (details in Supplementary Table S4). All of the studies were carried out in the United States. The size of study population varied from 548 to 120 877.^6, 100^ The latter article reported a combined analysis from three cohort studies with similar measures. Seven of the studies reported on adults^7, 95, 96, 99, 100^ and five on children.^94, 97, 98, 101, 102^ All the studies used diet beverages as their exposure measure but reported a range of outcome measures. Follow-up ranged from one year^94^ to 20 years.^96^

**Figure 2:**
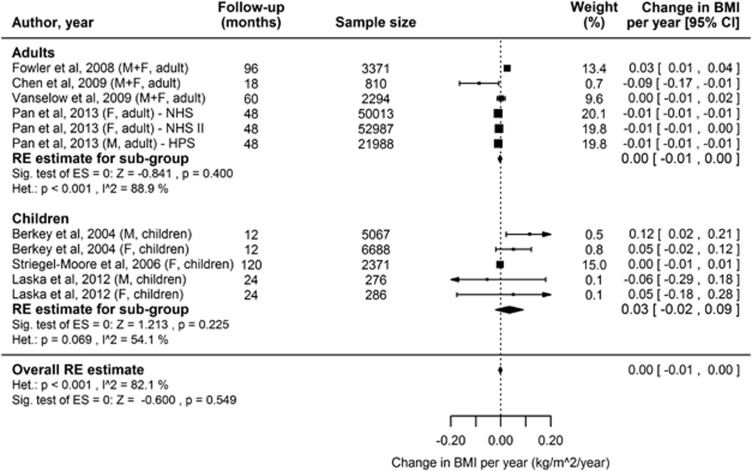
Forest plot showing individual and combined effect sizes for prospective cohort studies reporting the association between LES consumption and change in BMI over the follow-up period. Effect sizes have been standardised to a 1 year follow-up period. Negative scores favour LES consumption (BMI decrease). Squares represent change in BMI per year for the individual studies; square size is proportional to the weight of each study; horizontal lines represent 95% CIs. Diamonds represent the summary estimates and 95% CIs from random effects models for associations in adults and children separately, and in adults and children combined. BMI, Body Mass Index; LES, low-energy sweetener.

Five studies reported a higher risk of obesity associated with consumption of LES.^7, 94, 96, 99^ The higher risk was present in boys but not girls in one of these studies,^94^ and was substantially attenuated after adjustment for dieting and parental weight concern in another study.^102^ Six studies reported a lower risk of obesity associated with consumption of LES.^95, 98, 100, 101^ In three of these studies there was only weak evidence against the null (no effect) hypothesis.^95, 98, 101^ One study reported a higher risk in girls but a lower risk in boys, though in both analyses there was limited evidence against the null hypothesis.^97^ The three largest studies were reported as a pooled analysis.^100^ In this pooled analysis there was evidence against the null hypothesis (*P*=0.03). The effect size was modest (0.10 kg BW reduction over a 4-year period per serving per day of diet soda) with a wide 95% confidence interval (CI) (−0.14 to −0.06 kg). We were able to combine data from 9 of the 12 studies^7, 94, 95, 97, 100, 101, 102^ in meta-analyses. In total there were six comparisons in adults and five in children. The random effects model (Figure 2) showed no change in BMI with LES consumption. However, there was a high level of heterogeneity among the studies. The fixed effect model showed a slightly lower BMI with LES consumption (−0.008 kg m^−2^ per year, 95% CI −0.010 to −0.006) (Supplementary Table S5). An assessment of funnel plot asymmetry using Egger's regression test suggests there is weak evidence of asymmetry (Supplementary Figure S1 and Supplementary Table S6), meaning smaller studies may have been more likely to report an increase in BMI.

### Commentary

In the 12 studies meeting our criteria, the associations reported were not consistent. Similar numbers of studies reported associations in each direction with respect to risk of BW gain or obesity. The largest studies reported a lower risk of obesity associated with consumption of LES, but the effect size was small.^100^ The random effects meta-analysis showed no change in BMI with consumption of LES.

Because the associations of interest may not have been a primary purpose of relevant studies, it is possible that our search strategies did not identify all articles that reported on LES as part of a report of the associations between diet and obesity. This omission would be more likely if the results were null and reported only as a line in the text. Nevertheless, the addition of another one or two studies, unless very large, would have limited effect on the present conclusions.

All the studies included in the review reported on the frequency of consumption of diet beverages and did not attempt to estimate consumption of LES in foods. Therefore, total intake of LES may not have been estimated accurately. It is possible that associations between ‘diet' beverages and obesity represent confounding by other characteristics or behaviours of people who consume these beverages. Furthermore, people who are overweight or have a propensity to put on weight may consume ‘diet' beverages in an attempt to lose weight or reduce weight gain. If reverse causality explained some or all of the association then adjustment for baseline BW or dieting or eating concerns would attenuate the association. This was the case in the analysis reported by Vanselow *et al.*^102^ Bias seems unlikely as this would imply that people using LES and with obesity will be more or less likely to be lost to follow-up. Generalisability is an issue as all the eligible studies were carried out in the United states, where diet beverages are commonly consumed and obesity prevalence is high.

The results from prospective studies of LES, BW and obesity are inconsistent. Observational studies are difficult to interpret as associations may be due to confounding or reverse causality. Even so, taken together there is little evidence from these studies to conclude that LES increase the risk of BW gain or obesity.

## Short-term (⩽1 day) intervention studies/RCTs

### Study selection and data extraction and analysis

Eligibility criteria for short-term intervention studies were: LES exposure of ⩽24 h and EI measured in an *ad libitum* meal (test meal) after consumption of the LES (preload) compared with a ‘control' condition. We extracted data for test meal EI after the LES preload and comparison preload(s), and for the energy content of the preloads. We also noted the preload-to-test meal interval, and the number of participants and their gender, and where available their age, BW and/or BMI, dieting and/or dietary restraint status.

We categorized the extracted data into five types of comparisons: LES versus sugar, LES versus unsweetened products, LES versus water, LES versus nothing and LES in capsules versus placebo capsules. We conducted meta-analyses to derive summary estimates of differences in cumulative EI (preload plus test meal, kcal) separately for each of these types of comparison. For LES versus sugar comparisons we also derived summary estimates of compensation index (COMPX) scores.^103^ COMPX (EI in test meal after LES minus EI in test meal after sugar)/(EI from sugar preload minus EI from LES preload) is expressed as percentage. It describes the extent to which adjustment in test meal intake ‘compensates' for the difference in energy content of the LES versus sugar preload. If COMPX is <100% then LES led to under-compensation (reduced cumulative EI), if COMPX >100% then LES led to over-compensation (increased cumulative EI), compared with sugar.

For LES versus sugar there was a good number of studies that tested children, so for this comparison we stratified the analysis by age group (child or adult participants). Fuller details of study selection, data extraction and statistical methods used are available in the Supplementary Information.

### Results

We identified 56 eligible articles,^83, 104, 105, 106, 107, 108, 109, 110, 111, 112, 113, 114, 115, 116, 117, 118, 119, 120, 121, 122, 123, 124, 125, 126, 127, 128, 129, 130, 131, 132, 133, 134, 135, 136, 137, 138, 139, 140, 141, 142, 143, 144, 145, 146, 147, 148, 149, 150, 151, 152, 153, 154, 155, 156, 157, 158^ which yielded a total of 218 comparisons. Of these, 118 were between LES and sugar (sucrose, glucose, fructose and mixtures of sugars including high-fructose corn syrups). In a majority of the comparisons the participants were young, lean, low dietary restraint, non-dieting adults. The LES and sugar were most often given in a beverage (83% of studies). Within individual comparisons, the sweetness of the LES and sugar preloads was similar.

Details of the studies are shown in Supplementary Tables S7–S11. The results of the meta-analyses are summarised in Figure 3, forest and funnel plots are shown in Supplementary Figures S2–S8, and results of meta-regression analyses are shown in Supplementary Table S12.

#### LES versus sugar

**Figure 3:**
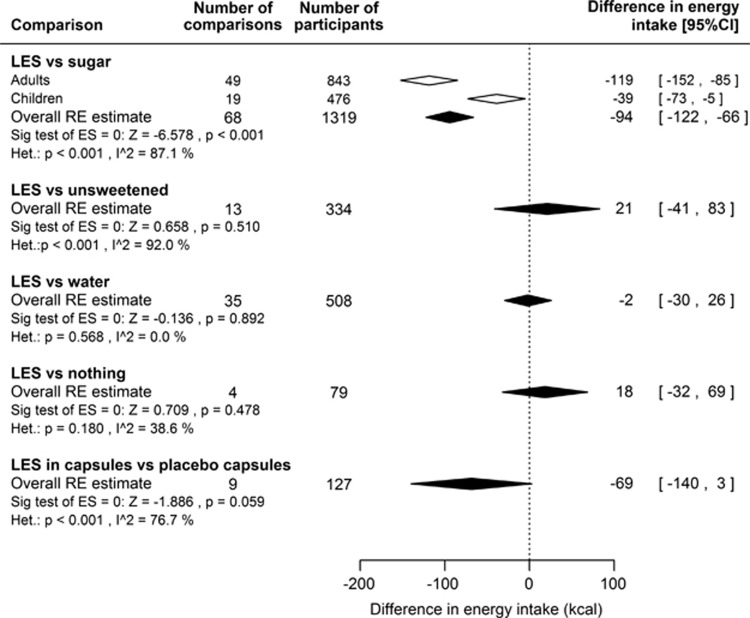
Summary of outcomes of meta-analyses of short-term intervention studies comparing the effects on EI of LES versus sugar (for adults and children separately and combined), LES versus unsweetened products, LES versus water, LES versus nothing and LES in capsules versus placebo capsules. EI difference is the difference in cumulative EI (total of preload plus test meal EI) for the LES condition minus the comparison condition. Negative scores favour LES (that is, lower cumulative intake with LES). Filled diamonds represent the summary estimates and associated 95% CIs from random effects models of all studies included in the comparison. Unfilled diamonds represent the summary random effects estimates and 95% CIs for studies of adults and children separately. Many of the included studies reported multiple results for the same participants within the same comparison (for example, LES versus several different sugars). Treating these multiple results as independent potentially biases estimates of the variance of the summary effect sizes (see Supplementary Information methods section). Therefore, only the first set of results reported from each study was analysed. Accordingly, the total number of comparisons analysed (129) is less than the total recorded (218). EI, energy intake; LES, low-energy sweetener.

Cumulative (preload plus test meal) EI was reduced with consumption of LES versus sugar preloads in adults and in children (Figure 3). The smaller absolute difference for children is partly accounted for by the lower energy content of the sugar preloads given to children compared with those given to adults. Children also showed somewhat greater compensation (COMPX score) than adults (Supplementary Figure S3). Compensation for the sugar preloads was 70% (95% CI 43 to 97%) in children and 43% (95% CI 31 to 55%) in adults, and significantly different from zero and from 100% in both groups. In other words, there was partial but not full compensation for the lower energy content of the LES compared with sugar preloads.

There was a high level of heterogeneity among the studies. A fully adjusted multivariable meta-regression model found no statistically significant associations of COMPX with year of publication, energy content of the sugar-containing preload or gender, and only weak evidence of an association with interval between preload and test meal (Supplementary Table S12). An assessment of funnel plot asymmetry using Egger's regression test suggests there is some asymmetry (Supplementary Figure S8 and Supplementary Table S6), meaning smaller studies may have been more likely to report larger COMPX scores. This bias may affect studies in children more than those in adults.

#### LES versus unsweetened, LES versus water and LES versus nothing

Cumulative EI did not differ when a LES-sweetened preload was consumed compared with when a preload of the same, but unsweetened product was consumed. Likewise, there was no difference in cumulative EI with consumption of LES-sweetened beverages versus water and with LES-sweetened beverages versus nothing (Figure 3).

There was a high level of heterogeneity within these sets of studies, reflecting inconsistency in effect sizes, which is apparent in Supplementary Figures S4–S6. For LES versus water (the largest number of studies), multivariable meta-regression showed no clear associations between difference in cumulative EI and gender, interval between preload and test meal, or year of publication (Supplementary Table S12). Egger's regression test and visual inspection of funnel plots indicate that bias is unlikely to have greatly affected these studies (Supplementary Table S6, Supplementary Figure S8), although this assessment is limited by the relatively small sample sizes of the studies and high heterogeneity.

#### LES in capsules

LES given in capsules compared with placebo capsules tended to reduce EI (Figure 3). In all but one^149^ of the comparisons analysed the LES was aspartame. There was a high level of heterogeneity among studies. Multivariable meta-regression indicated a potential association between the difference in cumulative EI and the interval between the capsule and test meal. An interval of >30 min compared with ⩽30 min was associated with a decrease in EI (Supplementary Table S12), although this finding may be unreliable due to the small number of comparisons analysed.

#### Sensitivity analyses

The sensitivity analyses (Supplementary Table S13) show that our summary effect sizes were unaffected by our estimates of unreported standard deviations, and fixed effect models (Supplementary Table S5) were broadly consistent with the main random effects models. Accounting for repeated measures on the same participants using robust variance estimation methods did not significantly alter the summary effect sizes.

### Commentary

Taken together, these results show that consumption of LES in place of sugars is consistently found to reduce short-term EI. Contrary to the concern that LES might increase intake acutely through stimulation of subsequent EI by sweetness or via other mechanisms (reviewed in Mattes and Popkin^1^), EI did not differ for LES versus water, LES versus unsweetened product or LES versus nothing. The high-observed heterogeneity in the effect sizes may be reasonably ascribed to methodological variations, only some of which could be assessed here. Preload size (difference in energy content), interval between preload and test meal, cross-over effects and nature of the test products, population and meal, have all been reported to influence absolute and relative (compensatory) EI in these types of studies.^8, 148, 159^

A limitation of these studies is that they measure intake at a single meal only, so they may miss adjustments in intake that occur subsequently. Against this, our analysis showed, if anything, reduced compensation with increasing length of interval between consumption of LES and the meal (except for the capsule studies, which is not how LES are typically consumed). Another possible limitation is that almost all studies used cross-over designs. Evidence suggests that this causes the impact of disguised nutrient manipulations to be underestimated.^148, 160^ This, however, may apply less to LES versus water comparisons. A further possibility is that on repeated exposure the perceived satiating effect of (and preference for) a LES-containing beverage or food will decrease relative its higher energy, sugar-containing counterpart.^161, 162, 163^ This again suggests that the sustained effect on EI of LES versus sugar consumption may be less than is indicated by short-term (single exposure) studies.

Unlike in everyday life, the beverage or food in these studies was not presented with any explicit information (for example, ‘diet' or ‘low calorie') identifying either its energy content or the sweetener used. It would be counterproductive if dietary restraint were relaxed as a result of a perceived ‘saving of calories' through consuming LES.^1, 11, 12, 14, 164^ Only two articles addressed this particular issue. Neither found a difference in the effect of LES versus sugar on test meal EI in participants who were informed about energy value of the preload compared with those who were not given this information.^147, 157^

The studies comparing LES to unsweetened products, water or nothing suggest that the exposure to sweetness itself was not a significant stimulus for later EI. However, the studies in which LES were consumed in capsules (in amounts similar to that contained in one or two portions of a LES-containing beverage) identify a reduction in EI after aspartame that is not apparent for other LES.^149^ The mechanism for this effect is unknown^156^ but, more generally, these studies reduce the possibility that LES might increase appetite via stimulation of ‘sweet taste' receptors in the gastrointestinal tract.^1, 165^ Despite *in vitro* and animal research related to the physiological effects of gut sensing of nutrients and gustatory (mainly sweet or bitter) stimuli, LES appear to be generally weak stimuli for gut sensing in humans.^166, 167, 168^ This conclusion is supported by a large number of studies reporting only limited effects of various LES on glycaemic, insulinaemic and gut hormone responses.^105, 123, 146, 169, 170, 171^ It is worth noting that, consistent with the suppressive effect on EI of aspartame in capsules, repeated consumption of aspartame in capsules (2.7 g per day for 13 weeks) led to a 1.1 kg greater BW loss in young adults placed on an energy-restricted diet than did placebo capsules.^172^

In sum the results of these short-term studies comprise a large body of evidence showing that consumption of LES in place of sugar reduces overall EI acutely, with no indication that LES increase appetite. They do not test the effect of repeated, sustained exposure to LES. This information is provided by longer-term intervention studies, which are reviewed next.

## Sustained (>1 day) intervention studies/RCTs

### Study selection and data extraction and analysis

All studies with a LES exposure period of >1 day were classified as ‘sustained' interventions. Studies were included only if (1) there was an explicit instruction or requirement to consume LES-sweetened foods or beverages as an alternative to or substitute for consumption of sugar-sweetened products, water or habitual diet (the comparison treatment); (2) participants had *ad libitum* access to (other) dietary energy sources; and (3) reported end points included EI and/or anthropometric measures. Studies furthermore had to have a parallel or balanced-ordered cross-over design with healthy participants (regardless of BW), who were either uninformed or correctly informed of the manipulation (not deceived). A study duration of ⩾4 weeks was applied as a further criterion for inclusion in a meta-analysis of LES effects on BW. As BW is the more reliable indicator of sustained effects on energy balance, no meta-analysis was undertaken for EI. Assessments of risk of bias and methodological quality were also undertaken for these studies.

### Results

#### Energy intake

There were nine studies comprising 10 comparisons and 1102 participants that reported EI results.^173, 174, 175, 176, 177, 178, 179, 180, 181^ In all except one study^181^ the participants were adults, and all but four of the studies^174, 177, 180, 181^ specifically recruited overweight or obese participants. In two studies^173, 179^ the research was undertaken in the context of a weight loss programme. The duration of exposures was 10 days to >1 year. For all treatment comparisons (LES vs directly relevant controls) in all studies, the LES group had the lowest absolute values for total or change in EI, compared with either sugar or water (Supplementary Table S14). The magnitude of the difference in intakes reported for LES vs non-LES interventions ranged from −75 to −514 kcal per day for comparisons with sugar, and −126 kcal per day in the single comparison with water.^179^

In three comparisons with 318 participants, the difference in total EI was reported or determined to be statistically significant.^176, 177, 181^ In three comparisons with 293 participants the difference was not reported as statistically significant.^173, 175, 178^ In the remaining comparisons a direct test of statistical significance was not reported and could not be determined.^174, 179, 180^

#### Anthropometric

**Figure 4:**
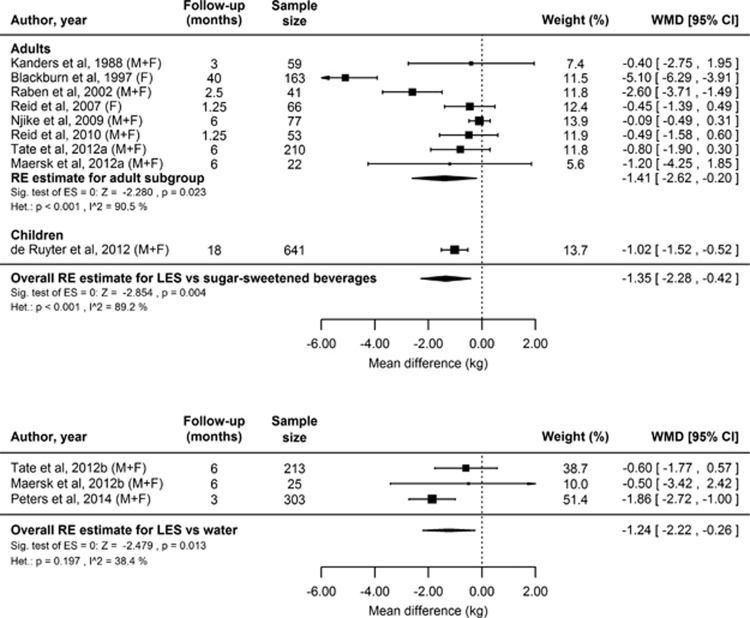
Forest plots showing individual and combined effect sizes for sustained intervention studies comparing the effects on BW of LES versus sugar (upper panel) and LES versus water (lower panel). Mean difference is weight change (end point minus baseline) in the LES condition minus weight change in the sugar condition over the intervention period (a negative score favours LES). Squares represent mean difference in BW for the individual comparisons; square size is proportional to the weight of each comparison; horizontal lines represent 95% CIs; diamonds represent the summary estimates and 95% CIs from random effects models for comparisons in adults for LES versus sugar, adults and children for LES versus sugar, and adults for LES versus water. BW, body weight; LES, low-energy sweetener.

Twelve studies comprising 14 comparisons with 1941 participants reported anthropometric data,^173, 174, 175, 176, 177, 178, 179, 180, 182, 183, 184, 185^ in most cases only BW. This total excludes 67 intentionally misinformed participants in Reid *et al.*^177^ The duration of exposures was 10 days to >3 years, and participants in all studies were adults except for those in de Ruyter *et al.*^182^ All except four studies^175, 177, 180, 182^ specifically recruited overweight or obese participants, and four studies^173, 179, 183, 185^ carried out the interventions in the context of a weight loss programme. Details of these studies are shown in Supplementary Table S14. The results of the meta-analyses of the 10 studies (12 comparisons) of ⩾4 weeks in duration are shown in Figure 4.

In all comparisons the LES intervention produced the smallest absolute gain or largest absolute loss of BW, compared with either sugar or water. In six comparisons with 1274 participants, anthropometric differences favoring LES were reported to be statistically significant.^173, 176, 177, 180, 182, 185^ In eight comparisons with 667 participants the differences were not reported as statistically significant.^175, 176, 178, 179, 183, 184^

The main meta-analysis of BW change comparing sustained use of LES vs sugar-sweetened products is based on eight comparisons with 691 adults and one comparison with 641 children. In our random effects model we found a relative BW loss in adults using LES products compared with sugar-sweetened products (Figure 4). In the only study with children there was a comparable effect size and same direction of effect, so the overall random effects model (adult and child studies combined) also found that participants who consumed LES products compared with sugar-sweetened products showed relative reductions in BW (greater loss or less gain) (Figure 4).

There was a high degree of heterogeneity among the studies, which arises from differences in the magnitude and not direction of effect, which was consistent across all studies. The overall estimates and measure of heterogeneity were little changed by removal of the one study with children. A multivariable meta-regression model of change in BW on gender (studies with males only, females only or both), BW category (all weights, overweight and obese only or obese only) and length of follow-up suggested these study characteristics are not important sources of heterogeneity (Supplementary Table S15). Egger's regression test (Supplementary Table S6) and visual inspection of funnel plots (Supplementary Figure S9) suggest that small-study bias is unlikely to be present. However, these assessments of bias and sources of heterogeneity are limited by the small number of studies and high level of heterogeneity.

The secondary meta-analysis of BW change using LES products versus water was based on three comparisons with 541 adults. The random effects model indicates a statistically significant reduction in BW in adults using LES products compared with water, with an effect size only slightly smaller than that observed for comparisons with sugar-sweetened products (Figure 4).

Sensitivity analyses suggested that using imputed standard deviations did not substantially alter the outcomes of the analyses (details in Supplementary Table S16).

The results of the ‘risk of bias' and ‘methodological quality' assessments are summarised in Supplementary Tables S17 and S18. They highlight that full blinding of the interventions (for participants and some personnel) was not possible in many studies. However, most papers also did not explicitly state whether outcome assessors were blinded to the interventions, or the methods of randomisation and allocation. Furthermore, there was inconsistent reporting of the nature of dropouts, and some studies only reported on participants fully completing the treatment arm(s) despite significant (>10%) dropouts.

### Commentary

Although these sustained intervention studies vary in design and quality, and several were not primarily intended to test effects of consumption of LES, the results are nevertheless consistent. In all cases, the use of LES led to a relative reduction in EI, and greater loss (or reduced gain) of BW. Notably, there was no example of a sustained exposure intervention trial where LES use led to a relative *increase* in mean EI or BW. This was supported quantitatively by the results of the meta-analyses of BW change, indicating lower relative BWs in LES intervention arms. Furthermore, outcomes were similar in studies with children and adults, and followed a similar pattern whether participants were blinded or not blinded to the intervention. Consumption of LES-sweetened beverages also reduced BW relative to consumption of water.

In addition to the studies meeting the criteria for inclusion here, there are also studies in adults and children where LES were a component of a mixed or more complex diet or lifestyle manipulation.^95, 186, 187, 188, 189, 190^ Although these other studies provide only indirect evidence, their results were consistent with the ‘pure' LES interventions; that is, treatments that included LES were associated with reductions or no change in EI or BW. No example was found where an intervention specifically including LES led to a relative increase in EI or BW.

Taken at face value, these results show that consumption of LES compared with sugar leads to either no change or a relative reduction in EI and energy balance. More unexpectedly, however, LES also led to reductions in BW relative to water. There are, however, some possible limitations to this evidence base to consider.

First, most protocols^175, 176, 177, 178, 179, 180, 182, 184^ required participants to consume some amount of test products with LES or sugar. If the latter constitute an energy supplement to the diet, and if adding energy to the diet tends to be poorly compensated,^191, 192^ this design will overestimate the benefit of LES. However, similar outcomes were observed where participants were recruited on the basis of already regularly consuming sugar-sweetened products, where the intervention was replacement of those by LES.^179, 182^ Thus, both ‘supplementation' and ‘substitution' interventions lead to similar conclusions.

Second, in most comparisons of LES with sugars^174, 175, 176, 180, 181, 182, 184^ participants were blind to the intervention. This does not model the ‘real-world' consumption of such products, where consumers are usually aware of choosing a LES-sweetened (‘diet') or sugar-sweetened product. In this situation, awareness of reductions in EI from LES products may be mitigated by relaxed restraint towards consumption of energy from other items in the diet. Against this, results of blinded studies did not differ appreciably from comparisons with sugar (or water) where participants were aware of the nature of the intervention products.^173, 177, 179, 183, 184, 185^ This is consistent with the short-term intervention studies and two studies of 4 weeks duration, which reported no effect of participants being specifically informed or misinformed as to beverages being LES or sugar-sweetened.^177, 178^ Thus, the evidence indicates that effects of LES on EI or BW are probably not markedly affected by awareness.

Third, the comparison of LES with water is based on only three studies.^179, 184, 185^ Of these, two were large studies carried out in a BW loss context, in which participants who habitually consumed either sugar-^179^ or LES-^185^ sweetened beverages were assigned to consume water.

BW differences were greatest in the longest study, which lasted 40 months,^173^ as can be seen in Figure 4, although there is no clear relationship between study duration and BW loss among the other studies in adults (duration 1.25–6 months). Together the studies included males and females, and lean, overweight and obese participants. We found no evidence that gender or BW status affected the outcome of the intervention, indicating that the effects of consuming LES versus sugar-sweetened are general, rather than specific to certain groups of individuals. Similarly, the recent large study by de Ruyter *et al.*^182^ is significant in that it shows an impact on BW of LES versus sugar consumption in children. Indeed, the effect in this study in favour of LES was close in size to the combined effect size for the studies in adults (Figure 4).

## Discussion

Table 2 summaries the results of this review. It shows that the weight of the evidence indicates decreased EI and BW with consumption of LES relative to sugar. Furthermore, LES do not appear to increase EI, and in human interventions studies tend to lead to a relatively decreased BW, even compared with consumption of water in the groups tested. These findings are consistently contrary to the concern that has been raised by some authors^5, 6, 7^ that consumption of LES may increase risk of becoming overweight and obese. The results in favour of a benefit of LES use in BW management come primarily from a substantial number of short-term and sustained intervention studies testing human participants, which can be reasonably said to rank above observational studies and animal studies as sources of evidence for informing health-care decisions, including dietary recommendations. In any case, close examination of the totality of the evidence including that from observational and animal studies does not contradict the conclusion that LES use is helpful, or at least neutral, with respect to BW control.

A recent systematic review and meta-analysis of the evidence from prospective cohort and sustained intervention studies on LES and BW and composition^193^ differs from ours in the studies included and excluded for both sources of evidence (see Supplementary Tables S19 and S20 for further details), and in not considering the evidence from short-term intervention studies and animal studies, or the specific comparison with water. The authors, nevertheless, reach similar conclusions to us. They state that ‘substituting LCS (low-calorie sweeteners) options for their regular-calorie versions results in modest BW loss, and may be a useful dietary tool to improve compliance with BW loss or BW maintenance plans'.^193^

We found that prospective associations between LES consumption and BW change were not consistent across the literature, with beneficial and adverse associations of LES use with BW reported by similar numbers of studies. This could be due to differences in the populations studied and/or the extent to which specific confounding factors were accounted for, chance findings in small studies and publication bias. A major confounder may be the BW control history and strategies used in the different populations. Thus, reverse causation cannot be ruled out. Notably, though, pooled analysis of the three largest studies showed a relative BW reduction for those consuming LES.^100^

Evidence from a large majority of animal studies favours reduced EI and BW gain or no effect of LES, although increases have been observed as well. A particular series of studies from one research group employing a specific procedure in rats has been widely cited in support of the suggestion that consumption of LES could lead to BW gain.^194^ The mechanism proposed for this is that LES consumption disrupts learning about the association between sweet taste and energy content. Arguably, however, the complexity of modern human diets is such that sweetness, with or without the inclusion of LES, does not reliably predict energy content foods.^195^ More likely, what is learned, if anything, is energy content (and its satiating effect) signalled by a configuration of taste, flavour, texture and other cues specific to individual foods. Therefore, whether or not LES are consumed will likely make little difference to the learned control of EI. In any case, without information from further control groups the interpretation of the results of these studies is ambiguous. Their relevance to human consumption of LES therefore remains to be demonstrated, especially in the light of the evidence from sustained human intervention studies of reduced BW with consumption of LES versus sugar, and LES versus water. In general, the animal studies did not mimic the use of LES by humans, either in amounts consumed or pattern of consumption.

A variety of human and animal intervention studies provided data on the effects of consumption of LES-sweetened liquids versus water. The results indicate that the exposure to sweetness itself (without energy) is at least neutral in terms of impact on EI and energy balance, and possibly even helpful. This challenges the suggestion that exposure to sweetness itself is detrimental,^6, 196, 197^ perhaps because exposure to sweetness could help ‘establish and maintain' a more generalised preference for sweet items.^1^ Acquired preferences for sweetness levels are relatively food-specific, although this is expressed against the background of a general innate liking for sweetness.^198^ On the other hand, evidence from studies of ‘sensory-specific satiety' show that acute exposure to sweetness decreases subsequent desire for the same or other sweet (relative to non-sweet) items.^199, 200, 201^ Consistent with this, participants in the CHOICE trial receiving LES beverages specifically reduced consumption of dessert items relative to those receiving water.^202^ One possible interpretation is that access to LES satisfies a pre-existing desire for sweetness, rather than promoting it.

A further result is that LES consumed in capsules either had no effect or reduced EI in short-term intervention studies (Figure 3), which argues against stimulation of gut receptors as a route by which LES might contribute toward increased EI and BW.

The short-term intervention studies in humans measured the effects of consuming a fixed portion of a LES-sweetened beverage or food on free (*ad libitum*) consumption of other items presented in the test. In many of the animal studies the manipulated food was also the food that was consumed freely. For the most part the results of these two types of study lead to the equivalent conclusion, namely that there was little effect of the LES versus the equi-caloric control (usually water in the human studies, and unsweetened food in the animal studies) on EI or BW. An exception is increased EI and BW gain in a small number of studies in which saccharin was added to moistened or fat-enriched diets, perhaps owing to saccharin increasing the palatability of these diets. This context differs from the consumption of LES in the human diet, where LES usually replace sugars in foods and beverages.^1^ Furthermore, as discussed above, there is no substantive evidence that consuming LES-sweetened products increases EI or BW in humans.

Our analysis of short-term intervention studies comparing LES with water supports Daniels and Popkin's^27^ conclusion that these studies are ‘relatively consistent and found no impact on EI among adults' (p 505). Their review of a subset of the studies we included found that consumption of water versus LES-sweetened beverages led to essentially no change (1.3% reduction) in EI in adults and a 6.7% reduction in children. Nevertheless, our meta-analysis of three sustained intervention studies with a water comparison finds that LES consumption leads to somewhat reduced BW relative to consumption of water.^179, 184, 185^ As well as an effect via ‘sensory-specific satiety' (above), this perhaps suggests an advantage for LES in respect of dietary compliance, in that LES beverages may be preferred to water.^179^ It would seem appropriate therefore to consider, particularly for consumers already accustomed to sweetened beverages, whether promotion of LES or water as an alternative holds greatest public health effectiveness.^203^ The answer should come from further appropriately designed intervention studies and perhaps further analyses of already completed trials where participants had free choice of water versus LES beverages.^187^

Both short-term and sustained intervention studies in humans show LES consumption reduces *ad libitum* EI. However, possibly for several reasons, short-term studies may underestimate the degree of energy compensation that occurs in response to energy dilution. This is consistent with the finding that BW differences observed in sustained intervention studies are often much smaller that would be the case if no compensation had occurred.^182^ Nonetheless, sustained studies repeatedly find effects in favour of LES consumption reducing BW relative to sugar consumption, showing that compensation is incomplete. Of course, the same argument and many of the same data are used in making the case that consumption of sugar-sweetened products, and particularly sugar-sweetened-beverages, increase risk of overweight and obesity.^182, 204, 205^

## Conclusions

We found a considerable weight of evidence in favour of consumption of LES in place of sugar as helpful in reducing relative EI and BW, with no evidence from the many acute and sustained intervention studies in humans that LES increase EI. Importantly, the effects of LES-sweetened beverages on BW also appear neutral relative to water, or even beneficial in some contexts.

A selection of animal and observational studies is often cited as the primary basis for strong assertions that LES are a contributing factor toward risk of overeating and obesity.^5^ In contrast, the present review of a large and systematically identified body of evidence from human intervention studies, with varying designs, settings and populations (including children and adults, males and females, and lean, overweight and obese groups), provide no support for that view. The question then is whether those hypotheses should be rejected or whether, as seems unlikely, the relevant human intervention studies are consistently flawed in a way that leads, in most cases, to exactly the opposite outcome.

Commentaries on LES and energy balance frequently suggest that further research is needed, but stop short of proposing any specific new hypothesis to test or new study designs. Although no single study by itself is conclusive, the correspondence of results from the studies reviewed here gives no reason to expect another similar study would yield remarkably different results. Continued selective citation and extrapolation from observational and animal studies on this topic is also likely to be of limited value. Mattes & Popkin^1^ concluded that replacement of sugar by LES has ‘the potential to aid in BW management, but whether they will be used in this way is uncertain' (p 10). This seems a reasonable conclusion from the literature, and shifts the issue from whether LES are ‘good' or ‘bad,' and re-focuses it on the question of how they are best used in practice to help in the achievement of specific public health goals, such as the reduction of intakes of free sugars and energy.

## Figures and Tables

**Table 1:** Outcomes of rodent studies providing information on effect of compulsory consumption of LES on BW

	*Change in BW relative to controls*		
	*Decrease*	*No difference*	*Increase*
At any dose (47)^a^	22	21	4
Lower LES doses (37)^b^	4	32	1
Higher LES doses (34)^c^	18	13	3

Abbreviations: BW, body weight; LES, low-energy sweetener.

Figures are numbers of studies reporting no difference or a significant change (increase or decrease) in BW relative to controls, with total numbers of relevant studies in brackets. Where a study used multiple LES doses (in some studies including doses both below and above 2% of vehicle), if the change in BW for any dose was significantly increased or decreased relative to placebo, that result was counted as the effect of the LES overall.

a All studies included in the count.

b Lower LES doses, ⩽2% of vehicle (diet or fluid source), only included in the count.

c Higher LES doses, >2% of vehicle (diet or fluid source), only included in the count.

**Table 2:** Summary of the results of the review

*Type and number of studies (or number of comparisons)*^a^	*Results*
Animal studies, 90	BW gain when LES added to food or drink compulsorily or voluntarily consumed compared with BW gain on the food or drink without LES: 22↓ 37→ 9↑
	BW gain when LES added to a dietary supplement compared with BW gain when glucose added to the same dietary supplement: 0↓ 3→ 19↑
Prospective cohort studies, 12	No overall association of LES consumption with BMI
Short-term intervention studies (129 comparisons analysed^b^)	EI from preload plus *ad libitum* meal when preload was LES versus sugar, unsweetened food, water, nothing or placebo capsule: LES<sugar (children and adults) LES=unsweetened LES=water LES=nothing LES in capsule<placebo capsule (trend)
Sustained intervention studies, EI (10 comparisons)	In all cases the absolute value for total or change in EI was lower for LES: LES versus sugar −75 to −514 kcal per day (nine comparisons) LES versus water −126 kcal per day (one comparison)
Sustained intervention studies ⩾4 weeks in duration, BW (12 comparisons)	Difference in weight loss or weight gain favoured LES: LES versus sugar −1.41 kg (adults, eight comparisons) LES versus sugar −1.02 kg (children, one comparison) LES versus water −1.24 kg (adults, three comparisons)

Abbreviations: BMI, body mass index; BW, body weight; EI, energy intake; LES, low-energy sweeteners.

↓ decrease; → no difference; ↑ increase.

a Some studies had more than one relevant comparison, for example LES versus sugar and LES versus water, or LES versus sugar in lean participants and LES versus sugar in overweight participants reported separately.

b See caption to Figure 3.

## Notes

Peter J Rogers has received grants from Sugar Nutrition, UK in support of research on the effects of sugar on human appetite. Cees de Graaf has received grants from the Dutch Sugar Bureau in support of a study on brain responses to sugars and low energy sweeteners. Suzanne Higgs has received a grant from Canderel in support of research on the effects of low-energy sweeteners on human appetite. Anne Lluch and David J Mela are employees and shareholders of companies that manufacture products containing sugars and low-energy sweeteners. Peter Putz is an employee of ILSI Europe. The remaining authors declare no conflict of interest.
