# Supplementary material for: Does low-energy sweetener consumption affect energy intake and body weight? A systematic review, including meta-analyses, of the evidence from human and animal studies
Source: Int J Obes (Lond). 2015 Nov 10;40(3):381–94. doi: 10.1038/ijo.2015.177 (PMC4786736; doi:10.1038/ijo.2015.177)
Supplement: Supplementary Information [file ijo2015177x1.pdf]

Contents

Definition of low-energy sweeteners (LES) 4

Medline search terms 5

Systematic review and meta-analysis methods 6

Overall methods for the systematic review 6

Data extraction methods for observational (prospective cohort) studies in humans 6

Data extraction methods for short-term intervention studies in humans 6

Summary of sustained intervention studies in humans 7

Meta-analysis methods – overview 8

Core meta-analysis methods 8

Specific methods for the observational (prospective cohort) studies 9

Specific methods for the short-term intervention studies 10

Specific methods for the sustained-intervention studies 11

Table S1. Characteristics and results of animal studies with compulsory consumption of LES and information on the effects of LES on BW 12

Table S2. Characteristics and results of animal studies with voluntary consumption of LES and information on the effects of LES on BW 25

Table S3. Characteristics and results of animal learning studies on the effects of LES on EI and BW 32

Table S4. Characteristics of prospective cohort studies reporting information on association between LES consumption and body weight status change 42

Table S5. Summary of meta-analysis fixed effect results for the various types of human studies 54

Table S6. Summary of results of Egger's regression test for funnel plot asymmetry for the various types of human studies 55

Table S7. Characteristics and results of short-term intervention studies comparing the effects on EI of LES versus sugar 56

Table S8. Characteristics and results of short-term intervention studies comparing the effects on EI of LES versus unsweetened products 84

Table S9. Characteristics and results of short-term intervention studies comparing the effects on EI of LES versus water 89

Table S10. Characteristics and results of short-term intervention studies comparing the effects on EI of LES versus nothing 100

Table S11. Characteristics and results of short-term intervention studies comparing the effects on EI of LES in capsules versus placebo capsules 101

Tables S12. Results of meta-regression analyses for the short-term intervention studies 104

Sensitivity analyses for short-term intervention studies 109

Table S13. Summary of sensitivity analyses for short-term intervention studies 110

Table S14. Characteristics and results of sustained intervention studies comparing the effects on EI and/or anthropometric measures of LES versus sugar and LES versus water 112

Table S15. Results of meta-regression analyses for the sustained intervention studies 120

Table S16. Summary of sensitivity analyses for sustained intervention studies 121

Table S17. Summary of risk of bias assessments for the sustained intervention studies 122

Table S18. Summary of methodological quality assessment for sustained intervention studies 123

Comparison of the present review with Miller and Perez[(144)] 124

Table S19. Prospective cohort studies that differed in inclusion/exclusion between the present review and the review by Miller and Perez[(144)] 124

Table S20. Sustained intervention studies that differed in inclusion/exclusion between the present review and the review by Miller and Perez[(144)]. 125

Figure S1. Funnel plot of prospective cohort studies reporting information on association between LES consumption and body weight status change. 126

Figure S2. Forest plot of short-term intervention studies comparing the effects on EI of LES versus sugar in children and in adults. 127

Figure S3. Forest plot of short-term intervention studies comparing the effects on COMPX of LES versus sugar in children and in adults. 129

Figure S4. Forest plot of short-term intervention studies comparing the effects on EI of LES-sweetened versus unsweetened products. 131

Figure S5. Forest plot of short-term intervention studies comparing the effects on EI of LES versus water. 132

Figure S6. Forest plot of short-term intervention studies comparing the effects on EI of LES versus nothing. 133

Figure S7. Forest plot of short-term intervention studies comparing the effects on EI of LES in capsules versus placebo capsules. 134

Figure S8. Funnel plots of short-term intervention studies comparing the effects on EI of LES versus sugar (for adults and children separately). 135

Figure S9. Funnel plots of sustained intervention studies comparing the effects on BW of LES versus sugar and LES versus water. 136

Reference List 137

Definition of low-energy sweeteners (LES)

We defined LES as sweeteners and sweetener systems that contribute negligible energy to the product (i.e., typically <15% of the 'standard' or control caloric sweetener system, in most cases sucrose[(1)]). Clearly included were products where sweetness is predominantly derived from intense and non-caloric sweeteners (e.g., saccharin, aspartame, cyclamate, sucralose, acesulfame-K, stevia, erythritol) as direct replacements for sugars. We did not separately assess or compare effects of these different specific sweeteners, as they represent a diversity of molecular structures and there is limited hypothetical basis and empirical evidence to make such comparisons. Excluded were products using fructose or other caloric sugars or sweetener systems (e.g., intense sweeteners together with maltodextrins or sugars) in place of sucrose to achieve more limited reduction in energy content.

Medline search terms

1. Body Weight/ or Energy Intake/ or Energy *Metabolism*/ or Obesity/
2. (weight and (control or gain$ or los$ or change$ or increas$ or reduc$)).ti,ab.
3. (body adj1 (weight or fat or composit$)).ti,ab.
4. Adipo$.ti,ab.
5. BMI.ti,ab.
6. waist circumferenc$.ti,ab.
7. lean body mass.ti,ab.
8. percentage body fat.ti,ab.
9. corpulen$.ti,ab.
10. fat.ti,ab.
11. obes$.ti,ab.
12. overweight.ti,ab.
13. over weight.ti,ab.
14. slim$.ti,ab.
15. (weight adj6 (cyc$ or reduc$ or los$ or maint$ or decreas$ or watch$ or control$ or gain$ or chang$)).ti,ab.
16. waist-hip ratio.ti,ab.
17. exp abdominal fat/
18. ((food or energy or calor$) and intake$).ti,ab.
19. 1 or 2 or 3 or 4 or 5 or 6 or 7 or 8 or 9 or 10 or 11 or 12 or 13 or 14 or 15 or 16 or 17 or 18
20. *Saccharin/
21. cyclamat$.ti,ab.
22. acesulfame-k.ti,ab.
23. stevia.ti,ab.
24. (sugar adj1 replacer).ti,ab.
25. *Sweetening Agents/
26. erythritol.ti,ab.
27. ((sugar-free or sugar free or reduced-sugar$) and (product$ or food$ or beverag$ or drink$)).ti,ab.
28. ((artificial$ or intens$ or high-intens$ or non-calori$ or reduced- or reduc$ or low-energ$ or low-calori$) and sweetener$).ti,ab.
29. aspartame$.ti,ab.
30. sucralos$.ti,ab.
31. 20 or 21 or 22 or 23 or 24 or 25 or 26 or 27 or 28 or 29 or 30
32. 19 and 31

Systematic review and meta-analysis methods

Overall methods for the systematic review

We excluded duplicates and obviously irrelevant articles on the basis of the article title or abstract, and allocated the remaining articles to subgroups of studies for review. Articles were included or excluded based initially on review of the abstract and then, where required review of the full report. Additional articles were identified by searching the references lists of reviews and included articles. We performed meta-analyses of the observational and the short-term and sustained intervention studies. Details of the processing of the data for each subgroup of studies are given in the relevant study selection and data extraction sections in the main text and below. In those sections we refer to 'articles' (i.e., a published paper), each of which included one or more separate 'studies' (i.e., experiments) or 'comparisons' (e.g., LES versus sugar, or LES versus water).

Data extraction methods for observational (prospective cohort) studies in humans

Data from all of the eligible studies were extracted and agreed by two co-authors, and tabulated by date of publication. We recorded the size of the study as the number of participants included in the analysis and we also documented the number of participants enrolled in the study. Where various outcomes were reported we preferred reports of change in anthropometric outcome and those reported over the longest follow up period. Results of the fully adjusted model are reported, and where adjustment made a marked difference we note this. We considered age, gender, baseline anthropometric data, dieting behavior and social position to be key potential confounders. If possible, multiple adjusted models that did not adjust for EI were selected, as this is likely to be the primary mediator of any association between LES consumption and the anthropometric outcomes.

Data extraction methods for short-term intervention studies in humans

Data extraction was undertaken by three co-authors according to a mutually agreed final protocol based on comparison and resolution of individual extracted data from ten randomly selected articles in the list. Data were extracted for test meal EI after the LES preload and comparison preload(s), and for energy content of the preloads (see below). We also noted the preload to test meal interval, and the number of participants and their gender, and where available their age, weight and/or BMI, dieting and/or dietary

restraint status. Where results were reported for subgroups within the same article (gender, weight status, preload amount, preload to test meal interval) we extracted the data separately for these subgroups. We extracted the data on the amount eaten on the first eating occasion (test meal) after the first preload, except in 4 studies (Van Wymelbeke et al.[(2)]; Appelton & Blundell[(3)]; Beridot-Therond et al.[(4)]; Lavin et al.[(5)]) in which beverage preloads were served between meals throughout the day. In these instances we included the data for total preload and total test meal intake. We also included studies in which the preload was consumed with the test meal.

Summary of sustained intervention studies in humans

Data extraction was undertaken and agreed by two co-authors. We identified 13 studies (15 comparisons) meeting the inclusion criteria (Blackburn et al.[(6)],; de Ruyter et al.[(7)]; Kanders et al.[(8)]; Maersk et al.[(9)] Naismith & Rhodes[(10)]; Njike et al.[(11)]; Peters et al.[(12)]; Raben et al.[(13)] Reid et al.[(14)]; Reid et al.[(15)] Tate et al.[(16)]; Tordoff and Alleva[(17)]; Wolraich et al.[(18)], reporting EI data for 10 comparisons and anthropometric (weight) data for 14 comparisons. In the majority of studies a clear comparison was made between LES- and sugar-sweetened test products, which were provided to participants by the investigators (de Ruyter et al.[(7)]; Maersk et al.[(9)]; Naismith & Rhodes[(10)]; Njike et al.[(11)]; Raben et al.[(13)]; Reid et al.[(14)]; Reid et al.[(15)]; Tordoff & Alleva[(17)]; Wolraich et al.[(18)]). Maersk et al.[(9)] additionally had a water intervention group, while Peters et al.[(12)] compared groups instructed to consume either LES beverages or water. In these comparisons of LES with water, blinding of participants was clearly not possible. Furthermore, Blackburn et al.[(6)] and Kanders et al.[(8)] compared participants advised to use or discouraged from using commercially available LES products during weight control programs. Tate et al.[(16)] placed regular consumers of sugar-sweetened beverages in treatment groups provided with either LES beverages or water, or maintaining their own choice of beverage. Participants in all other LES vs sugar comparisons were not informed of (i.e., blinded to) these treatments, with the exception of the 2 studies from Reid et al.[(14,15)] where half the participants were correctly informed and half deliberately misinformed. The misinformed subjects from Reid et al.[(14)], were excluded from the meta-analysis of body weight, but could not be excluded from a smaller study by this group[(15)], where the (mis)information had 'no effect on the results' and was not included or reported in the original paper. Additional treatment arms with dissimilar products (low fat milk in Maersk et al.[(9)] a placebo beverage in Njike et al.[(11)]) or no intervention (in Tordoff & Alleva[(17)]) were disregarded, as were data from

Experiment 1 (n=6) in Naismith & Rhodes[10], which did not use a counter-balanced treatment order. Final rather than interim EI and BW values were extracted wherever possible.

Meta-analysis methods – overview

The same core method was used in the meta-analyses of the observational, short-term and sustained intervention studies. The methods relating to further specific secondary analyses and sensitivity analyses relevant to the observational, short-term and sustained intervention studies are detailed after the core methods.

Core meta-analysis methods

Estimation of missing values for the accuracy of estimation of the study-level effect sizes

For the meta-analyses, along with the effect size estimates, we extracted the associated standard deviations (SDs). Where SDs for the effect sizes were not reported, these were imputed using the following methods:

1) For studies which reported appropriate test statistics (e.g. t values from t-tests) we derived the missing SDs. If studies only reported *P* values we estimated the appropriate test statistics using available conversion tables and then estimated SDs as previously described.

2) For studies which reported only the SDs for the comparison arms (i.e. SDs for pre and post intervention measures in the short-term interventions, or SDs for control and intervention arms in the sustained interventions) we estimated correlation coefficients using the equation described by Higgins & Green[19] and data from studies which reported SDs for both the effect size and the comparison arms of the study. Missing effect size SDs were then imputed using the mean of these correlation coefficients and the reported SDs for the comparison arms.

3) For studies which did not report any SDs we assumed the SDs were equal to the largest SD from those studies which did report effect size SDs. This method can potentially bias results towards a lack of effect[19], and therefore sensitivity analyses were carried out using less conservative estimates of the missing SDs and excluding those studies with missing SDs.

Sensitivity analyses

In order to determine the effect of imputing missing SDs on the summary effect size estimates we repeated the analyses with two modifications and compared these results with the main analyses. Firstly, instead of replacing missing SDs with the largest reported SDs we used the mean of the reported SDs. Secondly, studies which did not report any SDs were excluded from the analyses.

Statistical methods

Specific methods for the observational (prospective cohort) studies

If possible, multiple adjusted models that did not adjust for energy intake were selected. Fowler, Pan et al.(20,21) and Striegel-Moore et al.(22) did not adjust for energy intake. Berkey et al.(23) and Laska et al.(24) reported change in BMI both unadjusted and adjusted for energy intake, we have included the unadjusted results. Chen et al.(25) Duffey et al.(26) Ludwig et al.(27) Nettleton et al.(28)and Vanselow et al.(29) only included results adjusted for energy intake.

We excluded Ludwig et al.(27) from the meta-analyses since they did not report regression coefficients or confidence intervals related to change in BMI or body weight. While they did report odds ratios for obesity incidence, no other studies reported this outcome measure. Nettleton et al.((28)and Duffey et al.(26)reported hazard ratios for high waist circumference, which is too few studies to conduct a reliable separate meta-analysis.

The main meta-analysis of observational studies involved a comparison of change in BMI with change in LES consumption. Since the follow-up time differed between studies we standardised the effect sizes and their associated standard errors to ‘change per year’ by dividing the effect sizes and standard errors by follow-up time (in years). Chen et al.(25)and Pan et al.(21) reported change in body weight, whereas Berkey et al.(23), Strigel-Moore et al.(22), Fowler et al.(20), Vanselow et al.(29) and Laska et al.(24) reported change in BMI. Therefore, change in body weight was converted into change in BMI using the mean reported height at baseline from each study. Where necessary(22), we converted the scale used to describe consumption of diet beverages from 100 g per day to one serving per day, assuming 355 g per serving. Fowler et al.(20) compared LES beverage consumers with non-consumers. We converted the median LES consumption of consumers (2.3 servings/day) into one serving per day by dividing the effect

size estimate by the median LES consumption, assuming a linear relationship between change in LES beverage consumption and change in BMI. Where possible we included effect size estimates for males and females separately and, in the case of Pan et al.[20], we included the effect sizes of the three study populations as separate comparisons. A random-effects model was used to calculate summary estimates, accounting for between-study heterogeneity. Considering the expected heterogeneity between the child and adult studies, analysis was stratified by study age group (adult or child).

Due to the low number of studies we did not explore factors which might explain the between-study heterogeneity with meta-regression models.

Specific methods for the short-term intervention studies

The SDs of the energy content of the LES and comparison preloads were assumed to equal zero. This assumption enabled us to calculate the cumulative energy intake and COMPX scores.

Many of the short-intervention studies reported multiple results for the same participants within the same comparison (e.g., LES versus different sugars: fructose 80% and glucose 20%, high fructose corn syrup, sucrose, glucose 80% and fructose 20%[30]). Due to the potential for correlation between these repeated measures, treating the effect size estimates as independent would result in an overestimate of the total population included in the meta-analyses and potentially biased estimates of the variance of the summary effect sizes. For the main analyses the first set of results from each study were used. In the sensitivity analyses we applied robust variance estimation methods[32] using the 'robumeta' package[32] to incorporate independent and repeated measures in the calculations of the summary effect sizes and meta-regression coefficients. Repeated measures were assumed to be correlated rather than hierarchical, and the default value for the within-study effect size correlation of $\rho=0.8$ was used.

The study-design variables considered in the meta-regression were year of publication, gender of participants (male, female or mixed), interval between consumption of the preload and consumption of the test meal (0 mins, <30 mins, 30-60 mins or >60 mins) and the energy content of the comparison preload. Meta-regression models were simultaneously adjusted for all independent variables.

Specific methods for the sustained-intervention studies

A meta-analysis was carried out on BW data, using studies with a minimum exposure of 4 weeks. Two studies meeting the inclusion criteria for the review[10,17] had an exposure duration of <4 weeks and are therefore noted in the narrative text and tables but excluded from the meta-analysis.

Two studies[16,9] were multi-armed interventions where a LES trial arm was compared to both a sugar and a water intervention arm, and a third study only compared LES beverages to water. Two separate meta-analyses were therefore undertaken, depending on the control group. The control group in the main meta-analysis was the trial arm consuming sugar-sweetened products. In the secondary meta-analysis the control group was the water consumption trial arm. A random-effects model was used to calculate summary estimates, accounting for between-study heterogeneity. Considering the expected heterogeneity between the child and adult studies, analysis was undertaken for each age group separately (adult or child) and together. Meta-regression models were simultaneously adjusted for all independent variables.

Table S1. Characteristics and results of animal studies with compulsory consumption of LES and information on the effects of LES on BW

Reference	Species tested	Design	Sample size per condition	Type LES	Dietary manipulation	Experimental conditions	Measures	Outcome
Dalderup & Visser[33]	Albino rats	LES vs different nutrients vs control	17	Sodium cyclamate	4.26mg/g cyclamate in place of sugar in powdered diet vs 5 control nutrients, 6 wks	1. control 2-5. added nutrients 6. added cyclamate	Total food intake<br><br>BW change (over 6 wks)	Sign. increase in BW relative for added cyclamate
Dalderup & Visser[34]	Albino rats	Cyclamate in place of sugar in powdered diet relative to different nutrients for 6 wks	17	Sodium cyclamate	4.26mg/g cyclamate in powdered diet versus 5 controls	1. control 2-5 added nutrients 6 added cyclamate	Max. BW	No sign. difference in BW between cyclamate and control: higher than those with sugar
Friedhoff [35]	Mice (strain not specified)	2% LES solution vs 13% sucrose solution vs water	10	2% sweetener solution (6% cyclamate, 0.6% saccharin)	LES solution as sole fluid source, 23 d		BW (measured 3 times/wk)	No group differences in BW
Brantom et al.[36]	ASH-CS1 mice	Chronic feeding of 4 doses of LES vs control	30	0.7, 1.75, 3-5 or 7.0 % sodium cyclamate	Daily dose of LES, 80 wks		BW	52 wks: no differences among groups<br>52-80 wks: BW loss for female mice with some doses

Reference	Species tested	Design	Sample size per condition	Type LES	Dietary manipulation	Experimental conditions	Measures	Outcome
Munro et al.[37]	Male and female Charles River rats	Between groups: dose-dependent saccharin vs control	120	0, 90, 270, 810, or 2430 mg saccharin/kg /d	Different concentrations of saccharin added to diet, 26 months	Group housed animals	BW<br><br>food intake	No effects on food intake<br><br>Highest doses caused weight loss
Oser et al.[38]	Male and female rats (no strain specified)	Between groups: dose-dependent cyclamate/saccharin mixture vs control	160	0, 500, 1120, and 2500 mg/kg cyclamate/saccharin mixture in ratio of 10:1	Different concentrations of cyclamate/saccharin mixture added to diet, 24 months	Group housed animals	BW<br><br>food intake	No sign. changes in food intake or BW
Andersen[39]	Weanling male Charles River rats	Between groups:<br>LES vs control	10	0, 1, 3, 5 and 7.5% sodium saccharin	LES (0, 1, 3, 5 and 7.5%) added to normal diet	Group housed animals	BW<br><br>food intake<br><br>recorded weekly	Sign. linear decrease in BW gain with increasing saccharin dose
Watkins et al.[40]	Sprague-Dawley rats, male	Between groups, LES vs control using different diets	5	Sodium saccharin	Saccharin added to diets enriched with safflower oil or beef tallow	Housed individually	BW<br><br>food intake	Lower BW gain with oil-enriched diet + saccharin vs oil-enriched diet<br><br>Higher BW on diet with beef tallow + saccharin vs tallow alone

Reference	Species tested	Design	Sample size per condition	Type LES	Dietary manipulation	Experimental conditions	Measures	Outcome
Ishii et al.[(41)]	Wistar rats	Between groups: 4 conditions LES vs control	86	0, 1, 2, and 4 mg/kg aspartame, or 4mg/kg aspartame + 1mg/kg DKP	Different concentrations of LES added to diet	Group-housed rats assigned to control, 3 doses of aspartame or combined aspartame/DKP	BW<br><br>food intake<br><br>recorded weekly	Slower BW gain after 2 and 4 mg/kg aspartame, and 4mg aspartame + DKP<br><br>Reduced food intake in all aspartame treatments
Higginbotham et al.[(42)]	Sprague Dawley CD rats	Between groups: 3-doses of thaumatin vs control	5	0, 0.3, 1 and 3% added thaumatin	Different concentrations of LES added to diet,<br>13 wks	Group housed animals consuming diets with LES *ad libitum*	BW<br><br>food intake (daily)	Higher BW male rats fed 3% thaumatin at 4 wks but not other wks<br><br>Lower BW female rats fed 1% thaumatin at 10 and 13 wks<br><br>Food intake “similar” in all groups (not reported)
Schoening et al.[(43)]	Male and female Charles River rats	Between groups dose-dependent saccharin vs control	980 M/ 1960 F, 10 groups, group min. 52 M, 104 F	0, 1, 3, 4, 5, 6.25, or 7.5 % sodium saccharin, or 5% in diet after gestation	Different concentrations of saccharin added to diet	Group housed animals	BW<br><br>food intake	Dose-dependent reduction in BW with saccharin
Fisher et al.[(44)]	F344 rats	LES vs calcium added to diet	10	5% calcium or sodium saccharin	LES added to normal diet,<br>10 w	6 conditions (2 diets x 3 sweeteners)	BW<br><br>food intake	Decreased BW gain in both groups fed saccharin<br><br>higher intake with one diet with added saccharin

Reference	Species tested	Design	Sample size per condition	Type LES	Dietary manipulation	Experimental conditions	Measures	Outcome
Lina et al.[45]	Male and female Wistar rats	Between groups: dose-dependent LES vs control	40	0, 0.2, 1 or 5% neohesperidin dihydrochalcone	Different concentrations of neohesperidin added to diet, 91 d	Group housed animals consuming diets with added LES *ad libitum*	BW<br><br>food intake<br><br>recorded weekly	Highest dose caused sign. reduction in BW
Xili et al.[46]	Male and female Wistar rats	-groups, dose-dependent effects of stevioside	45	0, 0.2, 0.6 and 1.2% stevioside	Different concentrations of stevioside added to diet,<br>2 years	Group housed rats	BW	No sign. differences
Lina et al.[47]	Male and female Wistar rats	Between groups dose-dependent erythritol vs control	100	0, 2, 5 and 10% erythritol	Different concentrations of LES,<br>52 wks	Group housed animals consuming diets with LES *ad libitum*	BW<br><br>group food intake	Sign. reduced BW with 10% added erythritol<br><br>clear trend for dose-dependent reduction

Reference	Species tested	Design	Sample size per condition	Type LES	Dietary manipulation	Experimental conditions	Measures	Outcome
Til et al.[48] (Exp. 1)	Male and female Swiss CD-1 mice and Wistar Crl (WI) WU BR rats	Between-groups, dose-dependent effects of erythritol	10/group for mice, 15/group for rats	0, 5, 10, or 20% erythritol	Different concentrations of LES added to diet, 90 d	Group housed mice. Additional condition with 20% mannitol for rats.	BW	Sign. reduction in BW after 20% erythritol in mice and rats
Til et al.[49] (Exp. 2)	Male and female Wistar rats	Between-groups, dose-dependent effects of erythritol	10	0, 5, and 10% erythritol	Different concentrations of LES added to diet in place of wheat starch, 4 wks	Group housed rats	BW<br><br>food intake	Reduced food intake and BW for male rats at 10% dose at start
Bailey et al.[50]	Homozygous lean (+/+) and obese-hyperglycaemic (ob/ob) mice, 5 wks old	Between groups: LES vs control	5	Sodium saccharine	1. Main study *Ad libitum* access to 5% saccharin in water (vs normal water), 7 wks<br><br>2. Suppl. study as main study, but with 1% saccharin	Group housed animals	BW<br><br>food intake (daily)	Lean mice: No sign. effect of 5% saccharin on BW or food intake<br><br>Obese mice: Reduced food intake and 18% reduction in BW, reversed by 4-wk washout<br><br>No sign. effects 1% diet

Reference	Species tested	Design	Sample size per condition	Type LES	Dietary manipulation	Experimental conditions	Measures	Outcome
Goldsmith [51] (Exp. 1)	Sprague Dawley CD rats	Between groups: 3 doses sucralose vs control	30	0, 1.0, 2.5 and 5% added sucralose	LES added to diet for 4-wk	Group housed animals consuming diets with sucralose *ad libitum*	BW<br>food intake<br>recorded weekly	Reduced BW with 5% sucralose in both male and female rats and with 2.5% sucralose for male rats only<br>No effects on food intake
Goldsmith [51] (Exp. 2)	Sprague Dawley CD rats	Between groups: 3 doses sucralose vs control	30	0, 1.0, 2.5 and 5% added sucralose	LES added to diet for 8-wk	Group housed animals consuming diets with sucralose *ad libitum*	BW<br>food intake<br>recorded weekly	Reduced BW with 5% sucralose<br>No effects on food intake
Beck et al.[52]	Male Long Evans rats	Between groups: aspartame vs control	12	Aspartame	Ad libitum access to 1% aspartame in drinking water or normal water for 14 weeks.	Group housed animals	BW<br>food intake (daily)	Significantly slower growth in rats consuming aspartame
Jeppesen et al.[53]	Adult male type-2 diabetic GK and Wistar rats	Between groups stevioside vs control	20	0.025g/kg/d stevioside in drinking water	*Ad libitum* access to LES in drinking water vs normal water, 6 wks	Housing unclear	BW	No sign. effects on BW

Reference	Species tested	Design	Sample size per condition	Type LES	Dietary manipulation	Experimental conditions	Measures	Outcome
Waalkens-Berendsen et al.[54]	Female Wistar Crl (WI)WU BR rats	Between-groups, dose-dependent effects of neohesperidin dihydrochalcone	28	0, 1.25, 2.5 and 5% neohesperidin dihydrochalcone	Different concentrations of LES added to diet, 21 d	Group housed mated female rats	BW	No sign. differences
Jurgens et al.[55]	NRMI mice, 3 months old	Combined LES treatment vs control	8-9	Combined sweetener (sodium cyclamate, aspartame, sodium saccharin)	*Ad libitum* access to LES soft drink or drinking water as fluid source, 73 d	Individually housed mice	BW<br><br>group food intake	No sign. difference in BW gain
Dyrskog et al.[56]	Obese ZDF rats	Stevioside in water vs control, combined with 2 diets	12	0.03g/kg stevioside	Stevioside solution vs water, combined with normal or high protein diet	1. normal + water<br>2. normal + stevioside<br>3. high protein<br>4. high protein + stevioside	BW<br><br>food intake (daily)	No significant effect of stevioside on BW
Tago et al.[57]	Male and female F344 Fischer rats	Between-groups, dose-dependent effects of oligo-N-acetylglucosamine	5	0, 0.2, 1, and 5% oligo-N-acetylglucosamine	Different concentrations of LES added to diet, 90 d	Group housed animals	BW<br><br>food intake	No sign. differences in BW or intake between LES and controls

Reference	Species tested	Design	Sample size per condition	Type LES	Dietary manipulation	Experimental conditions	Measures	Outcome
Bergheim et al.[58]	C57BL/J6 mice	LES in drinking water vs control	4-6	Combination of cyclamate, Sunett, saccharin	LES vs water, 8 wks		BW<br><br>food intake (daily)	No sign. differences in food intake or BW
Curry & Roberts[59]	HsdRcc Han:Wist rats	Chronic feeding of 4 doses of rebaudioside A vs control	10 male and 10 female, 4 wk study.<br><br>20M and 20 F 13 wk study	4 wk study: 0, 25,000, 50,000, 75,000 and 100,000 ppm rebaudioside A<br><br>13-wk study: 12,500, 25,000, and 50,000 ppm	LES added to diet<br><br>4-wk study, 13-wk study	4-wk study: 1 control, 4 doses<br><br>13-wk study: control, 3 doses	BW<br><br>food intake	4-wk study: BW reduced by highest dose<br><br>13-wk study: dose-dependent reduction in BW gain<br><br>reduced food intake
Nikiforov et al.[60]	Male and female Sprague-Dawley rats	Between groups: dose-dependent rebaudioside A vs control	160	0, 500,1000 or 2000 mg Rebaudiosid e A/kg/day	Different concentrations of LES added to diet,<br>90 d	Individually housed animals	BW<br>(at least twice/wk)<br><br>Food intake (recorded weekly)	Reduced BW of male rats on highest dose (2000 mg)<br><br>No effects on food intake

Reference	Species tested	Design	Sample size per condition	Type LES	Dietary manipulation	Experimental conditions	Measures	Outcome
Figlewicz et al.[61]	Albino rats	Sweetened liquids overnight, stevioside vs control	10	12.5% stevia	Sweetener as sole fluid source for 3 nights/wk	6 conditions with different sweeteners, only stevia vs control relevant	BW<br><br>food intake	No significant effect of stevia on BW.
Yagi and Matsuo[62] (Exp. 1)	Male Wistar rats	Between-groups, D-Psicose vs sucrose	18	3% D-Psicose	D-Psicose or sucrose added to diet,<br>12 months	Group housed rats	BW<br><br>food intake	No differences between treatments
Yagi and Matsuo[62] (Exp. 2)	Male Wistar rats	Between-groups, D-Psicose vs sucrose	10	3% D-Psicose	D-Psicose or sucrose added to diet,<br>18 months	Group housed rats	BW<br><br>food intake	No differences between treatments
Park & Cha[63]	Male C57BL/6J mice	Between groups: Stevia rebaudiana supplemented to high fat diet vs control	40	Stevia rebaudiana Bertoni extract	Extract from Stevia rebaudiana Bertoni given orally daily,<br>15 wks	Not clear if housed individually or in groups	BW (weekly)<br><br>food intake (daily)	No sign. difference in food intake or BW between high fat with stevia and high fat control group

Reference	Species tested	Design	Sample size per condition	Type LES	Dietary manipulation	Experimental conditions	Measures	Outcome
Park et al.[(64)]	Rat	Between groups: saccharin versus control	Not provided	Saccharin (0.1%)	*Ad libitum* access for 2h to saccharin alongside chow, 3 wks at postnatal day 22	Group house animals	BW<br><br>food intake (daily)<br><br>Saccharine intake	No sign. effects on BW
Geeraert et al.[(65)]	Mice, obese insulin-resistant	Stevioside vs control	Treatment 12, control 20	10mg/kg stevioside	Oral dosing, 12 w	Oral dosing rather than ingestion	BW	No effect of stevioside on BW
Andrejic et al.[(66)]	Wistar rats	Between groups: saccharin vs control	12	0.0005% saccharin	*Ad libitum* access to saccharin in drinking water or normal water for 6 weeks	Group housed animals	BW<br><br>food intake (group data)	No significant effect of saccharin on BW
Polyak et al.[(67)]	CBA/CA mice, male and female	Between groups	12 (60 total)	Saccharin, cyclamate, acesulfame -K or aspartame	*Ad libitum* access to one of 4 LES solutions or water	Group-housed animals	BW (weekly)<br><br>food intake	BW gain in M+F for saccharin vs control<br><br>BW gain male mice for cyclamate vs control

Reference	Species tested	Design	Sample size per condition	Type LES	Dietary manipulation	Experimental conditions	Measures	Outcome
Hlywka et al.[68]	Sprague Dawley CD rats	Between groups: 4 doses of monatin vs control	40	0, 5000, 10000, 20000 and 35000 ppm monatin	Different concentrations of LES added to diet, 90 d	Individually housed rats assigned to one of 5 diet conditions	BW<br><br>food intake<br><br>recorded weekly	Lower BW at end of trial after 35000ppm monatin, sign. in female rats only<br><br>No sign. differences in food intake
Otabe et al.[69]	Male and female Han-Wistar rats	Between groups: dose-dependent advantame vs control	55	0, 2000, 10000, and 20000 ppm advantame	Different concentrations of advantame added to diet, 104 wks	Group housed animals	BW<br><br>food intake	No sign. effects on food intake or BW
Otabe et al.[70]	Male and female Charles River rats	Between groups: dose-dependent advantame vs control	272	0, 2000, 10000, and 20000 ppm advantame	Different concentrations of advantame added to diet, 10 wks	Individually housed animals	BW<br><br>food intake	No sign. effects on food intake or BW
Reis et al.[71] (abstract in English)	Wistar rats	Between groups dose-dependent stevia vs control or sucrose	??	2, 4 or 6% added stevia or 4% sucrose	Stevia added to diet, 45 d	Group-housed rats	BW<br><br>food intake	No difference in BW among conditions

Reference	Species tested	Design	Sample size per condition	Type LES	Dietary manipulation	Experimental conditions	Measures	Outcome
Brathwaite et al.[(72)]	Pregnant Crl:CD(SD) rats	Chronic feeding of 3 doses of LES vs control	25	0, 15000, 30000 or 50000 ppm monatin	LES added to diet, 15 d	Group housed animals consuming diets with LES *ad libitum*	BW<br><br>Food intake recorded daily	Dose-dependent reduction in BW with added monatin
Nikiforov et al.[(73)]	Male and female Sprague-Dawley rats	Between groups: dose-dependent rebaudioside A and D vs control		0, 500,1000 or 2000 mg/kg/day rebaudioside D or 2000 my/kg/day rebaudioside A	Different concentrations of rebaudioside D or a single dose of rebaudioside A added to diet, 28 d	Individually housed animals	BW<br><br>food intake<br><br>recorded weekly	No effects of rebaudioside A or D on BW
Mitsutomi et al.[(74)]	Male C57Bl/6 mice	Between groups: LES vs water or sucrose	5	Combination of erythritol (99%) and aspartame (1%)	High-fat diet with LES vs water as fluid source, 4 wks	Group housed mice	BW<br><br>food intake<br><br>recorded daily	No difference in BW or food intake between sweetener and water<br><br>(enhanced BW but reduced food intake with sucrose)

Reference	Species tested	Design	Sample size per condition	Type LES	Dietary manipulation	Experimental conditions	Measures	Outcome
Fulop et al.[(75)]	White mice AKR2 line	Between groups LES versus control or "sugar": also sugar + LES condition	160 females and 160 males	80 mg/L sodium saccharin + 800 mg/L sodium cyclamate.	Ad libitum access to LES in drinking water	Group-housed for each condition and sex	Body weight over 52 weeks	No significant effects on BW
Abu-Taweel et al.[(76)]	Swiss-Webster mice	Between groups LES or LES + MSG versus control	10	Aspartame 32mg/kg	Ad libitum access to LES in tap water	Group housed	Body weight over 30 day exposure	Reduced growth with aspartame both alone and in combination with MSG
Palmnas et al.[(77)]	Sprague-Dawley rats	Between groups Aspartame versus water either with normal or HF diet	10-12	Aspartame 60mg/l	Ad libitum access to LES in tap water	Group housed	Body weight over 8 weeks	Reduced BW in rats on HF diet with ASP, no effect of ASP with normal diet

LES, low energy sweetener; BW, body weight.

Table S2. Characteristics and results of animal studies with voluntary consumption of LES and information on the effects of LES on BW

Reference	Species tested	Design	Sample size per condition	Type LES	Dietary manipulation	Experimental conditions	Measures	Outcome
Porikos & Koopmans (78)	Rats, female	Between groups	27 (81 total)	0.2% saccharin plus either 0.055, 0.11 or 0.22 aspartame	Solutions available alongside chow and water for 8 wks<br><br>After 8 wks each group subdivided:<br>1. continue on sweetened f`solution,<br>2. switch to other sweetened solution<br>3. sacrificed to determine body composition	Group 1: chow + water<br><br>Group 2 : 11% sucrose solution<br><br>Group 3: saccharin + one of doses aspartame		Higher BW sucrose rats than controls, no diff LES vs control.<br><br>When sucrose switched to sweetener: BW loss, when sweetener switched to sucrose: rapid BW gain (fat mass)<br><br>Food intake in line with BW

Reference	Species tested	Design	Sample size per condition	Type LES	Dietary manipulation	Experimental conditions	Measures	Outcome
Ramirez[79] (Exp. 1)	Charles River rats, female	Between groups	19	0.2% sodium saccharin	Addition of saccharin to wet diet (chow with added water) 6 weeks	Group-housed rats fed diet with 80% water added, with or without added saccharin	BW<br>food intake	Greater food intake and higher BW with LES relative to control
Ramirez[79] (Exp. 2)	Charles River rats, female	Between groups	10 or 11	0.2% sodium saccharin	Addition of saccharin to wet diet (chow with added water) 6 weeks	Group-housed rats fed diet with 80% water added, with or without added saccharin	BW<br>food intake	No significant differences in BW or food intake
Ramirez[79] (Exp. 4)	Charles River rats, female	Between groups	20 or 21	0.2% sodium saccharin	Addition of saccharin to high fat wet diet (chow with added water and oil) 6 weeks	Group-housed rats fed diet with 80% water added, with or without added saccharin	BW<br>food intake	Greater food intake and higher BW with LES relative to control
Ramirez[79] (Exp. 5)	Charles River rats, female	Between groups	16	0.2% sodium saccharin	Addition of saccharin to high fat sucrose-sweetened wet diet (chow with added water, sucrose and oil) 6 weeks	Group-housed rats fed diet with 80% water added, with or without added saccharin	BW<br>food intake	Greater food intake and higher BW with LES relative to control

Reference	Species tested	Design	Sample size per condition	Type LES	Dietary manipulation	Experimental conditions	Measures	Outcome
Ramirez[79] (Exp. 6)	Charles River rats, female	Between groups	10 or 11	0.2% sodium saccharin	Addition of saccharin to wet diet (chow with added water) 3 weeks, condition reversed after 3 weeks	Group-housed rats fed diet with 80% water added, with or without added saccharin	BW<br><br>food intake	Greater food intake and higher BW with LES relative to control over first 3 weeks but no differences between diets after diet reversal
Ramirez[79] (Exp. 7a)	Charles River rats, female	Between groups	9	0.5%sodium saccharin as proportion of solid diet	Addition of 0.5% saccharin to diet (chow) with added water 60% or 80%) for 3 weeks	Group-housed rats fed diet with 80% water added, with or without added saccharin	BW<br><br>food intake	No significant differences in BW or food intake
Ramirez[79] (Exp. 7b)	Charles River rats, female	Between groups	9	1.0% sodium saccharin as proportion of solid diet	Addition of 1.0% saccharin to diet (chow) with added water 60% or 80%) for 6 weeks	Group-housed rats fed diet with 80% water added, with or without added saccharin	BW<br><br>food intake	No significant differences in BW or food intake
Kanarek et al.[80]	Sprague Dawley CD rats	Between groups: saccharin vs control	9	0.15% sodium saccharin	*Ad libitum* access to 0.15% saccharin in drinking water or normal water for 20 days	Individually housed animals with access to saccharin + water vs water alone	BW<br><br>food intake (daily)	No significant differences in BW or food intake

Reference	Species tested	Design	Sample size per condition	Type LES	Dietary manipulation	Experimental conditions	Measures	Outcome
D'Anci et al.[81]	Male Long Evans rats	Sweetener in drinking water vs control	10	Sodium saccharin	0.15% saccharin vs water only, 3 wks	Sweetener vs normal tap water	BW	No significant difference in body-weight
Kanarek et al.[82]	Long Evans rats	Between groups: saccharin vs control	21	0.15% sodium saccharin	*Ad libitum access to 0.15% saccharin in drinking water or normal water for 3 weeks*	Individually housed animals with access to saccharin + water vs water alone	BW<br><br>food intake (daily)	No significant differences in BW or food intake
Kanarek et al.[82] (Exp. 1)	Long Evans rats	Between groups: saccharin vs control	11	0.15% sodium saccharin	*Ad libitum* access to saccharin in drinking water<br><br>4 wks	Individually housed animals with access to saccharin + water vs water alone	BW<br><br>food intake (daily)	No significant differences in BW or food intake
Kanarek et al.[83] (Exp. 2)	Long Evans rats	Between groups: saccharin vs control	8	0.15% sodium saccharin	*Ad libitum* access to saccharin in drinking water<br><br>3 wks	Individually housed animals with access to saccharin + water vs water alone	BW<br><br>food intake (daily)	No significant differences in BW or food intake

Reference	Species tested	Design	Sample size per condition	Type LES	Dietary manipulation	Experimental conditions	Measures	Outcome
Yeomans & Clifton(84) (Exp. 1)	Hooded wistar rats	Between groups	12	0.2% sodium saccharin	saccharin vs water or (10%) sucrose, 17 d	Rats given *ad libitum* access to LES as well as water	BW<br><br>food + fluid intake (daily)	No difference BW saccharin vs water<br><br>Lower BW gain of both saccharin and water vs sucrose
Yeomans & Clifton(84) (Exp. 2)	Hooded wistar rats	Between groups	12	0.2% sodium saccharin	saccharin vs water or (10%) sucrose, 15 d	Rats given *ad libitum* access to LES as well as water	BW<br><br>food + fluid intake (daily)	No difference BW saccharin vs water<br><br>Lower BW gain of both saccharin and water vs sucrose
Yeomans & Clifton(84) (Exp. 3)	Hooded wistar rats	Between groups	12	0.2% sodium saccharin	saccharin vs water or (10%) sucrose, 13 d	Rats given *ad libitum* access to LES as well as water	BW<br><br>food + fluid intake (daily)	No difference BW saccharin vs water<br><br>Lower BW gain of both saccharin and water vs sucrose

Reference	Species tested	Design	Sample size per condition	Type LES	Dietary manipulation	Experimental conditions	Measures	Outcome
D'Anci[85] (Exp. 1)	Male Long Evans rats	Sweetener in drinking water vs control	10	Sodium saccharin	0.15% saccharin vs water only, 3 wks	Sweetener vs normal tap water	BW<br><br>food intake (daily)	No significant differences in food intake or BW
D'Anci[85] (Exp. 2)	Male Long Evans rats	Sweetener in drinking water vs control	10	Sodium saccharin	0.15% saccharin vs water only, 3 wks	Sweetener vs normal tap water	BW<br><br>food intake (daily)	No significant differences in food intake or BW
Kanarek & Homoleski[86] (Exp. 1)	Long–Evans VAF rats, male and female	Between groups: saccharin vs control	18	0.15% sodium saccharin	*Ad libitum* access to 0.15% saccharin in drinking water vs normal water, 3 wks	Individually housed animals with access to saccharin + water vs water alone	BW<br><br>food intake (daily)	No significant differences in BW or food intake
Kanarek & Homoleski[86] (Exp. 2)	Long–Evans VAF rats, male and female	Between groups: saccharin vs control	16	0.15% sodium saccharin	*Ad libitum* access to 0.15% saccharin in drinking water vs normal water, 3 wks	Individually housed animals with access to saccharin + water vs water alone	BW<br><br>food intake (daily)	No significant differences in BW or food intake

Reference	Species tested	Design	Sample size per condition	Type LES	Dietary manipulation	Experimental conditions	Measures	Outcome
Feijó et al.[87]	Wistar rats	Yogurt with added aspartame, saccharin or sugar	10	Saccharin, aspartame	Supplementary sweetened foods with different LES	Aspartame, saccharin or sucrose sweetened supplement	BW<br><br>food intake	Higher BW gain saccharin and aspartame vs sucrose supplements, associated with increased chow intake

LES, low energy sweetener; BW, body weight.

Table S3. Characteristics and results of animal learning studies on the effects of LES on EI and BW

Reference	Species tested	Design	Sample size per condition	Type LES	Dietary manipulation	Experimental conditions	Measures	Outcome
Swithers & Davidson [88] (Exp. 1)	Rat	Between groups: predictive vs non-predictive vs sweet-only	8 in glucose (predictive) 9 in LES group (non-predictive) 10 in control (sweet-only)	Saccharin	30 g low-fat yogurt alongside chow + water, access 23 h/d for 5 wks<br><br>3 d for yogurt + chow and 1 day for chow + water only	Glucose: plain yogurt on 3 d/w, sweet yogurt (20% glucose) on 3 d/w<br><br>LES: plain yogurt on 3 d/w and sweet yogurt (0.3% saccharin) on 3 d/w<br><br>Control: sweet yogurt (20% glucose) on 3 d/w only	BW<br><br>energy intake<br><br>body composition	Higher BW gain LES vs glucose or control No sign. effects on food intake<br><br>Greater adiposity LES vs glucose and control
Swithers & Davidson [88] (Exp. 2)	Rat	Between groups: predictive vs non-predictive	11 in glucose (predictive) 9 in LES group (non-predictive)	Saccharin	30 g low-fat, plain yogurt alongside chow + water, access 23 h/d for 14 d	Glucose: plain yogurt on 7 d, sweet yogurt (20% glucose) on 7 d<br><br>LES: plain yogurt on 7 d, sweet yogurt (0.3% saccharin) on 7 d	BW gain<br><br>energy intake<br><br>energy compensation	Higher BW gain LES vs glucose<br><br>Higher energy intake LES vs sugar over course of training<br><br>Glucose rats showed caloric compensation by decreasing chow intake after novel yogurt

Reference	Species tested	Design	Sample size per condition	Type LES	Dietary manipulation	Experimental conditions	Measures	Outcome
Swithers et al.[89]<br><br>(Exp. 1)	Rat	Between groups: Saccharin vs AceK vs Glucose	8	Saccharin, AceK	30 g low-fat, plain yogurt alongside chow + water, access 23 h/d for 14 d	Glucose: plain yogurt on 7 d, sweet yogurt (20% glucose) on 7 d<br><br>LES (sacch): plain yogurt on 7 d, sweet yogurt (0.3% saccharin) on 7 d<br><br>LES (AceK): plain yogurt on 7 d, sweet yogurt (0.3% AceK) on 7 d	BW gain	Greater BW gain saccharin- or AceK vs glucose
Swithers et al.[89]<br><br>(Exp. 2)	Rat	Between groups: Saccharin vs AceK vs Glucose	11	Saccharin, AceK	20 g of yogurt for 1 h/d; 6 d/wk for 2 wks, with 1 d of chow + water (between 1st + 2nd wk)	Glucose: plain yogurt on 7 d, sweet yogurt (20% glucose) on 7 d<br><br>LES (sacch): plain yogurt on 7 d, sweet yogurt (0.3% saccharin) on 7 d<br><br>LES (AceK): plain yogurt on 7 d, sweet yogurt (0.3% AceK) on 7 d	BW gain	Greater BW gain saccharin- or AceK vs glucose

Reference	Species tested	Design	Sample size per condition	Type LES	Dietary manipulation	Experimental conditions	Measures	Outcome
Swithers et al.[(89)]<br><br>(Exp. 3)	Rat	Between groups: saccharin vs glucose<br><br>Additional factor: discontinuation of diets	13	Saccharin	20 g of yogurt for 1 h/d; 6 d/wk for 2 wks, with 1 d of chow + water (between 1st + 2nd wk)<br><br>After 2 wks, no more yogurt, but BW measured for 2 additional wks	Glucose: plain yogurt on 7 d, sweet yogurt (20% glucose) on 7 d<br><br>LES: plain yogurt on 7 d, sweet yogurt (0.3% saccharin) on 7 d	BW gain	Higher BW gain saccharin vs sucrose<br><br>Once yogurt presentation was discontinued, BW gain similar across the groups
Swithers et al.[(89)]<br><br>(Exp. 4)	Rat	Between groups: Base diet (yogurt vs beans), sweetener type (LES vs glucose), and phase (yogurt first vs beans first)	5 to 7	Saccharin	30 g of plain unsweetened diet +<br>30 g of sweetened diet for 23 h/d, 6 d/w (3 d sweetened + 3 days plain)	Group 1: glucose beans then glucose yogurt<br>Group 2: glucose beans then LES yogurt<br>Group 3: LES beans then glucose yogurt<br>Group 4: LES beans then LES yogurt<br>Group 5: glucose yogurt then glucose beans<br>Group 6: glucose yogurt then LES beans<br>Group 7: LES yogurt then glucose beans<br>Group 8: LES yogurt then LES beans	BW gain<br><br>energy compensation for pre meal	Phase 1: higher BW gain LES vs glucose rats regardless of diet (overall: beans group higher BW than yogurt group)<br>Phase 2: Glucose–glucose rats gained less BW than all other groups

Reference	Species tested	Design	Sample size per condition	Type LES	Dietary manipulation	Experimental conditions	Measures	Outcome
Swithers et al.[90]<br><br>(Exp. 1)	Rat	Between groups: saccharin vs glucose<br><br>Additional factor: caffeine added to diet	12 or 13	Saccharin	50 g of a flavored, sweetened liquid, 14 d	Group 1: glucose (20%)<br>Group 2: glucose + caffeine<br>Group 3: saccharin (0.3%)<br>Group 4: saccharin + caffeine	BW gain<br><br>energy intake	higher BW gain when access to LES vs glucose
Swithers et al.[90]<br><br>(Exp. 2)	Rat	Between groups: saccharin vs glucose<br>Additional factor: caffeine added to diet	8 or 9	Saccharin	High fat/sweet diet, with 30 g of sweetened liquid 6 d/w for 4 wks	Group 1: glucose (20%)<br>Group 2: glucose + caffeine<br>Group 3: saccharin (0.3%)<br>Group 4: saccharin + caffeine	BW gain<br><br>body fat % gain	Higher BW gain LES vs glucose
Davidson et al.[91]<br><br>(Exp. 2)	rats	Three diets (standard, standard + unsweet carb, standard + sweet carb) combined with supplement food	10	Saccharin	supplement sweetened with either glucose or saccharin on 50% of days	6 different diet/supplement combinations	BW change	Faster growth with saccharin vs glucose supplement
Swithers et al.[92]<br><br>(Exp. 1)	Rat	Between groups: saccharin vs glucose	15 for saccharin 16 for glucose	Saccharin	30 g plain unsweetened diet +<br>30 g sweetened diet,<br>6 d/w (3 d sweetened + 3 d plain)<br><br>Yogurt diets	Group 1 (LES): plain yogurt on 3 d/w, sweetened yogurt (0.3% saccharin) on 3 d/w<br><br>Group 2 (LES): plain yogurt on 3 d/w, sweetened yogurt (0.3% saccharin) on 3 d/w	BW gain<br><br>blood glucose response with and without test meal	Higher BW gain LES vs glucose<br><br>Rats previously given LES higher blood glucose levels following a test meal vs glucose

Reference	Species tested	Design	Sample size per condition	Type LES	Dietary manipulation	Experimental conditions	Measures	Outcome
					available for 3 h daily, for 14 d			
Swithers et al.[92] (Exp. 2)	Rat	Between groups: saccharin vs glucose	10 (blood glucose from 5/group)	Saccharin	30 g plain unsweetened diet + 30 g sweetened diet, 6 d/w (3 d sweetened + 3 d plain)<br><br>Yogurt diets available for 24 h daily, for 20 d	Group 1 (LES): plain yogurt on 3 d/w, sweetened yogurt (0.3% saccharin) on 3 d/w<br><br>Group 2 (LES): plain yogurt on 3 d/w, sweetened yogurt (0.3% saccharin) on 3 d/w	BW gain<br><br>blood glucose to oral glucose	Higher BW gain LES vs glucose<br><br>Higher blood glucose levels LES vs glucose (indicating an increase for levels in LES rats)
Swithers et al.[92] (Exp. 3)	Rat	Between groups: saccharin vs glucose	7 for saccharin 6 for glucose	Saccharin	30 g plain unsweetened diet + 30 g sweetened diet, 6 d/w (3 d sweetened + 3 d plain)<br><br>Yogurt diets available for 24 h daily, for 14 d	Group 1 (LES): plain yogurt on 3 d/w, sweetened yogurt (0.3% saccharin) on 3 d/w<br><br>Group 2 (LES): plain yogurt on 3 d/w, sweetened yogurt (0.3% saccharin) on 3 d/w	BW gain<br><br>glycemic responses to glucose (oral intake or delivered by gavage)	No differences BW gain<br><br>Higher blood glucose levels LES vs glucose rats following oral glucose load<br><br>No difference in response to gavage

Reference	Species tested	Design	Sample size per condition	Type LES	Dietary manipulation	Experimental conditions	Measures	Outcome
Swithers et al.[(92)] (Exp. 4)	Rat	Between groups: saccharin vs glucose	7 for saccharin 8 for glucose	Saccharin	30 g plain unsweetened diet + 30 g sweetened diet, 6 d/w (3 d sweetened + 3 d plain)<br><br>Yogurt diets available for 24 h daily, for 24 d	Group 1 (LES): plain yogurt on 3 d/w, sweetened yogurt (0.3% saccharin) on 3 d/w<br><br>Group 2 (LES): plain yogurt on 3 d/w, sweetened yogurt (0.3% saccharin) on 3 d/w	BW gain<br><br>glycemic responses to glucose (oral intake or delivered by gavage)	Day X Sweetener interaction but post-hoc testing did not reveal significant differences on any individual day for body weight<br><br>Higher blood glucose with oral glucose and taste + gavage, but not with gavage only.<br><br>No effect on insulin
Swithers et al.[(92)]<br><br>(Exp. 5)	Rat	Between groups: saccharin vs glucose	8	Saccharin	High fat/sweet diet, with 30 g of sweetened solution (0.3% saccharin vs 20% glucose) Solution available for 24 h daily, for 24 d	Group 1 (glucose): high fat diet + daily access to 10% glucose solution<br><br>Group 2 (LES): high fat diet plus daily access to 0.3% saccharin solution	BW gain<br><br>energy intake<br><br>glycemic responses<br><br>GLP1	Higher BW gain and greater food intake LES vs glucose<br><br>Blood glucose higher and GLP lower in LES vs glucose group, but only for oral and not for gavage test

Reference	Species tested	Design	Sample size per condition	Type LES	Dietary manipulation	Experimental conditions	Measures	Outcome
Swithers et al.[92]<br><br>(Exp. 6)	Rat	Between groups: saccharin vs glucose	23	Saccharin	High-fat/sweet diet, with 30 g plain unsweetened diet +<br>30 g sweetened diet 6d/w for 4 wks	Group 1 (glucose): plain yogurt on 3 days and yogurt sweetened with 20% glucose on 3 days per week<br><br>Group 2 (saccharin) plain yogurt on 3 days and yogurt sweetened with 0.3% saccharin on 3 days per week	BW gain<br><br>Body composition<br><br>glycemic response<br><br>GLP1	Higher BW gain and calorie intake LES vs glucose<br><br>Fat mass not affected<br><br>Higher blood glucose LES after presentation of the glucose<br><br>A trend for GLP 1 to be lower overall<br><br>No effect for insulin
Swithers et al.[93]<br><br>(Exp. 1)	Ovariectomised female rats	Between groups: saccharin vs glucose	13 for saccharin 12 for glucose	Saccharin	30 g plain unsweetened diet +<br>30 g sweetened diet 6d/w for 4 wks	Group 1 (glucose): plain yogurt on 3 days and yogurt sweetened with 20% glucose on 3 days per week<br><br>Group 2 (saccharin) plain yogurt on 3 days and yogurt sweetened with 0.3% saccharin on 3 days per week	BW gain<br><br>energy intake	No effect of sweetener on BW gain or energy intake

Reference	Species tested	Design	Sample size per condition	Type LES	Dietary manipulation	Experimental conditions	Measures	Outcome
Swithers et al.[93]<br><br>(Exp. 2)	Ovariectomised Female rats	Between groups: saccharin vs glucose<br><br>Additional factor: of age of Ovariectomy and inhibition of local estrogen production using Anastrozole	8 or 9 (adolescents)<br>12 (adults)	Saccharin	30 g plain unsweetened diet +<br>30 g sweetened diet 6d/w for 20 d (10 d sweet, 10 d unsweetend)	Group 1: adult, glucose, Anastrozole<br>Group 2: adult, glucose, Anastrozole vehicle<br>Group 3: adult, saccharin, Anastrozole<br>Group 4: adolescent, LES, Anastrozole vehicle<br>Group 5: adolescent, glucose, Anastrozole<br>Group 6: adolescent, glucose, Anastrozole vehicle<br>Group 7: adolescent, LES, Anastrozole<br>Group 8: adolescent, LES, Anastrozole vehicle	BW gain<br>energy intake<br><br>body composition	Four-way interaction for BW (Age × LES/gucose x Drug × Day)<br><br>Adult rats: Higher BW gain LES vs glucose, but only for Anastrozole. Adolescents rats: higher BW gain LES vs glucose, but only for vehicle group<br><br>Similar effect for total intake and fat mass
Swithers et al.[94]<br><br>(Exp. 1)	Female rats	Between groups: saccharin vs glucose<br><br>Additional factor: diet resistant (DR) and diet-induced obese (DIO)	10	Saccharin	30 g plain unsweetened diet +<br>30 g sweetened diet 6d/w for 4 wks	Group 1 (glucose): plain yogurt on 3 days and yogurt sweetened with 20% glucose on 3 days per week<br><br>Group 2 (saccharin) plain yogurt on 3 days and yogurt sweetened with 0.3% saccharin on 3 days per week	BW gain	No effect on BW gain

Reference	Species tested	Design	Sample size per condition	Type LES	Dietary manipulation	Experimental conditions	Measures	Outcome
Swithers et al.[94]<br><br>(Exp. 2)	Female rats	Between groups: saccharin vs glucose<br><br>Additional factor: diet resistant (DR) and diet-induced obese (DIO)	7 to 9	Saccharin	High-fat sweet diet, with 30 g plain unsweetened diet +<br>30 g sweetened diet 6d/w for 4 wks	Group 1: glucose, DR<br>Group 2: glucose, DIO<br>Group 3: LES, DR<br>Group 4: LES, DIO	BW gain<br><br>energy intake<br><br>body composition	Greater BW LES vs glucose, but only for DIO rats<br><br>Fat mass greater in LES vs glucose, but only for DIO rats<br><br>Both DIO and DR: Higher intake HE-chow LES vs glucose groups, but overall energy intake was higher in the DIO group only
Swithers et al.[94]<br><br>(Exp. 3)	Female rats	Between groups: saccharin vs glucose<br><br>Additional factor: diet resistant (DR) and diet-induced obese (DIO)	10 or 11	Saccharin	High-fat sweet diet, 30 g plain unsweetened diet +<br>30 g sweetened diet 6d/w for 4 wks	Group 1: glucose, DR<br>Group 2: glucose, DIO<br>Group 3: LES, DR<br>Group 4: LES, DIO	BW gain<br><br>body composition	Greater BW LES vs glucose but only for DIO rats<br><br>Greater fat mass LES vs glucose, but only in DIO-rats

Reference	Species tested	Design	Sample size per condition	Type LES	Dietary manipulation	Experimental conditions	Measures	Outcome
Swithers et al.[94]<br><br>(Exp. 4)	Male rats	Between groups: saccharin vs glucose<br><br>Additional factor: diet resistant (DR) and diet-induced obese (DIO)	10 to 12	Saccharin	High fat/sweet diet, 30 g plain unsweetened diet + 30 g of a sweetened diet for 16 d (8 d plain, 8 d sweetened), with 2 d of chow + water alone intervening	Group 1: glucose, DR<br>Group 2: glucose, DIO<br>Group 3: LES, DR<br>Group 4: LES, DIO	BW gain	Higher BW gain LES vs glucose for both DIO and DR male rats
Swithers et al.[94]<br><br>(Exp. 5)	female rats, offspring of adult obesity prone (OP) obesity resistant (OR) male and female rats	Between groups: saccharin vs glucose<br><br>Additional factor: diet resistant (DR) and diet-induced obese (DIO)	8 to 13 (44 total)	Saccharin	Yogurt supplements for 6 d (3 d sweetened + 3 d plain) along with 1 d of chow alone<br><br>Rats were then given *ad lib* access to sweetened HE diet assigned yogurt continued for 6 d/w for an additional 2 weeks	Group 1: glucose, DR<br>Group 2: glucose, DIO<br>Group 3: LES, DR<br>Group 4: LES, DIO	BW gain<br><br>body composition	Greater BW LES vs glucose, but only for OP rats<br>Greater fat mass gain with saccharin vs glucose

LES, low energy sweetener; BW, body weight.

Table S4. Characteristics of prospective cohort studies reporting information on association between LES consumption and body weight status change

**Authors, year**<br><br>**Cohort name**	**N with complete data, gender, age (range or mean)**	**Inclusion criteria (study population, location)**	**Dietary assessment method**	**Covariates (in adjusted models)**	**Duration, *n* at follow-up**	**Endpoint**	**Effect size**	**Notes**
Ludwig et al.[27]<br>Planet health intervention and evaluation project	n= 548<br>M / F<br>(unspecified)<br>11.7 y in 1995	Children, 6th or 7th grade on five schools<br><br>1995 - 1997<br><br>USA (Boston, 5 control schools)	**Youth FFQ**<br>(one question on diet soda)	Age, sex, baseline BMI + skin folds, ethnicity, school, dietary variables, physical activity, TV viewing	**19 months**<br><br>84% complete	**BMI**<br>regression coefficients	baseline and ΔBMI coefficients negative p=0.10	Association baseline obesity and diet soda NS (p=0.69)<br><br>Δdiet soda OR=0.44 (p=0.03)

Authors, year<br><br>Cohort name	N with complete data, gender, age (range or mean)	Inclusion criteria (study population, location)	Dietary assessment method	Covariates (in adjusted models)	Duration, *n* at follow-up	Endpoint	Effect size	Notes
Berkey et al.[23]<br><br>Growing up today study (GUTS)	n= 11 755<br>M / F<br>(unspecified)<br>9-14 y in 1996	Children, offspring of US Nurse's Health Study II<br><br>1996-1998<br><br>USA<br>(50 states)	**132-item FFQ** (beverages: diet soda, sugar-added drinks, fruit juice, milk)	Age, tanner stage, race, menarche, prior BMI z-score, height growth, milk, milk type, sugar added drinks, fruit juices, physical activity, inactivity	**1 y**<br>1996 to 1997, 1997 to 1998	**ΔBMI** self-reported regression coefficients	Boys: 0.116 (SE 0.049), p=0.016<br><br>Girls: 0.052 (SE 0.035), p=0.15<br><br>ΔBMI per serving of LES/d	Energy adjustment made no difference.

Authors, year<br><br>Cohort name	N with complete data, gender, age (range or mean)	Inclusion criteria (study population, location)	Dietary assessment method	Covariates (in adjusted models)	Duration, *n* at follow-up	Endpoint	Effect size	Notes
Striegel-Moore et al.[22]<br><br>US National Heart, Lung and Blood Institute Growth and Health Study (NGHS)	n=2371<br>0 M/ 2371 F<br>9-10 y at study entry<br><br>(1210 black, 1161 white)	Girls<br><br>1987-1997<br><br>USA (Schools in Berkeley, near Cincinnati, Washington DC; HMO and scouts)	**3-day food record** (diet sodas: artificially sweetened fizzy soft drinks except water, diet or low energy)<br><br>consecutive days:<br>1 weekend + 2 week days	Site, visit, race, milk, regular soda, fruit juice, fruit drinks, coffee/tea, energy intake	**10 y**<br>10 annual assessments<br><br>82% at visit 7, 89% at visit 10	**BMI** regression coefficient	-0.010 (SE 0.013)	

Authors, year<br><br>Cohort name	N with complete data, gender, age (range or mean)	Inclusion criteria (study population, location)	Dietary assessment method	Covariates (in adjusted models)	Duration, *n* at follow-up	Endpoint	Effect size	Notes
Fowler et al.[20]<br><br>San Antonio Heart Study	n= 3371<br>M / F<br>aged 25-64 y, NW/OW	Adults, random sample of residents<br><br>enrolled 1979-1988<br><br>USA (San Antonio, Texas)	Questions on sweeteners in soda, tea and coffee to quantify artificially sweetened beverages (ASB)	Age, gender, BMI, ethnicity, education, socioeconomic index, exercise, smoking cessation	**7-8 y**<br><br>65% (n=5158 enrolled)	**ΔBMI** regression coefficients (95% CI)	0.47 (0.26-0.66) in ASB users vs non-users	

**Authors, year**<br><br>**Cohort name**	**N with complete data, gender, age (range or mean)**	**Inclusion criteria (study population, location)**	**Dietary assessment method**	**Covariates (in adjusted models)**	**Duration, *n* at follow-up**	**Endpoint**	**Effect size**	**Notes**
Chen et al.[25]<br><br>PREMIER RCT (on blood pressure, BP)	n= 810<br>M / F (unspecified)<br>25-79 y (mean 50y)	Adults<br><br>2000-2002 (behavioral interventions)<br><br>USA (4 US centres, SBP 120-159 mmHg and DBP 80-95 mmHg)	**Two 24-hr recalls** (diet drinks: diet soft drink and other 'diet' drinks)<br><br>1 weekend, 1 weekday	Sex, race, age, income education, marital status, employment, BMI, all beverages, intervention, change in fitness and physical activity, total EI	**18 months**<br><br>recall at baseline, 6 and 18 months<br><br>no loss to follow up	**ΔBW** at 18 months regression coefficients (95% CI)	-0.38 (-0.22 - 0.01) for Δ1 serving/d of 355 ml diet drink)	Additional adjustment for dietary factors made no difference

Authors, year<br><br>Cohort name	N with complete data, gender, age (range or mean)	Inclusion criteria (study population, location)	Dietary assessment method	Covariates (in adjusted models)	Duration, *n* at follow-up	Endpoint	Effect size	Notes
Nettleton et al.[(28)]<br><br>Multi-Ethnic Study of Atherosclerosis (MESA)	n=2928<br>M / F<br>(unspecified)<br>45-84 y	Adults, white, black, Hispanic, Chinese<br><br>2000-2002<br><br>USA<br>(6 sites)	**FFQ**<br>(diet soda: diet soft drinks, unsweetened mineral water)	Study site, age, sex, race/ethnicity, education, energy intake, physical activity, smoking status, pack years, supplement use	**5 y**<br><br>n(n=6814 enrolled)	**High WC**<br>(M: ≥102 mc, F: ≥ 88cm)<br>Hazard ratio (95% CI)	1.59 (1.23 - 2.07)<br>(≥1 serving/d vs. rare/never)	Dose-response across WC categories

**Authors, year**<br><br>**Cohort name**	**N with complete data, gender, age (range or mean)**	**Inclusion criteria (study population, location)**	**Dietary assessment method**	**Covariates (in adjusted models)**	**Duration, *n* at follow-up**	**Endpoint**	**Effect size**	**Notes**
Vanselow et al.[29]<br><br>Project EAT (Eating Among Teens)	n=2294 (1032 M / 1262 F) 14.9 y	Adolescents<br><br>enrolled 1998-1999<br><br>USA (Minneapolis, 31 middle + public high schools)	**149-item FFQ** (with low-calorie soft drinks)	Age, cohort, sex, race, BMI, SES, baseline beverages, physical activity, TV watching, tea, coffee	**5 y** (follow-up in 2003-2004)<br><br>48.7% (n=4706 enrolled)	**ΔBMI** 5 yrs ≥ 7 serving/wk vs none	1.81 (SE 0.29) vs 1.80 (SE 0.09)	Attenuated with dieting and parent weight concern adjustment

**Authors, year**<br><br>**Cohort name**	**N with complete data, gender, age (range or mean)**	**Inclusion criteria (study population, location)**	**Dietary assessment method**	**Covariates (in adjusted models)**	**Duration, *n* at follow-up**	**Endpoint**	**Effect size**	**Notes**
Duffey et al.[26] Coronary Artery Risk Development in Young Adults (CARDIA) Study	n= 3524<br>M / F<br>(unspecified)<br>18-30 y	Young adults<br><br>1985-1986<br><br>USA<br>(4 sites)	**Diet history** questionnaire and quantitative diet history (with 'diet beverages' food group)	Race, sex, study centre, age, BMI, education, smoking status, family structure, total EI, physical activity	**20 y**<br><br>72%<br>(n=5115 enrolled)	**High WC**<br>(M: ≥102 mc, F: ≥ 88cm)<br>Hazard ratio (95% CI)	0.84 (0.73 - 0.97)<br>(non-consumer vs. consumer)	

Authors, year<br><br>Cohort name	N with complete data, gender, age (range or mean)	Inclusion criteria (study population, location)	Dietary assessment method	Covariates (in adjusted models)	Duration, *n* at follow-up	Endpoint	Effect size	Notes
Laska et al.[24]<br><br>Identifying determinants of activity (IDEA) and Etiology of Childhood Obesity (ECHO)	n= 535<br><br>M / F (unspecified)<br>14.6 y	Adolescents<br><br>2006-2008<br><br>USA (Minneapolis St Paul, Minnesota)	**Three 24-hr recalls** (diet drinks: ‘artificially sweetened’ soft drinks, fruit drinks, tea, coffee and/or coffee substitutes)<br><br>1 weekend, 2 weekday	Age, study, physical activity, puberty, race, parental education, eligibility for free/reduced price lunch	**2 y**<br><br>74% (n=723 enrolled)	**ΔBMI**<br>2 y servings /day	Males -0.11 (SE 0.24)<br><br>Females 0.10 (SE 0.23)	**ΔPBF**<br>2 y servings /day<br><br>Males -0.22 (SE 0.778)<br><br>Females 0.54 (SE 0.35)

Authors, year<br><br>Cohort name	N with complete data, gender, age (range or mean)	Inclusion criteria (study population, location)	Dietary assessment method	Covariates (in adjusted models)	Duration, *n* at follow-up	Endpoint	Effect size	Notes
Pan et al.[21]<br><br>Nurses' Health Study (NHS)	n=50 013<br>0 M/50 013 F<br>40-64 y	Nurses in 11 states, baseline 1986<br><br>USA	**FFQ**<br>(diet soda)	Age, BMI, sleep duration, physical activity, alcohol, TV viewing, smoking, dietary factors	**20 y**	**ΔBW** (95% CI) (self-reported)<br><br>Self-report weight<br><br>change in 4 y period<br><br>Pooled results across the three cohorts	-0.10 (-0.14 to -0.06) kg per serving/d increase	Age adjustment made no difference<br><br>Effect size slightly stronger in HPS and in overweight or obese people

Authors, year<br><br>Cohort name	N with complete data, gender, age (range or mean)	Inclusion criteria (study population, location)	Dietary assessment method	Covariates (in adjusted models)	Duration, *n* at follow-up	Endpoint	Effect size	Notes
Pan et al.[21]<br><br>Nurses' Health study II (NHS II)	n= 52 987<br>0 M/ 52 987 F<br>27-44 y	Younger nurses in 14 states, baseline 1991<br><br>USA			**16 y**			
Pan et al.[21]<br><br>Health Professionals Follow up Study (HPFS)	n= 22 988<br>22 988 M / 0 F<br>40-64 y	Male health professionals in 50 states, baseline 1986<br><br>USA			**20 y**			

BW, body weight; EI, energy intake; FFQ, food frequency questionnaire; IHD, ischemic heart disease; LES, low-energy sweeteners; NW, normal weight; OW, overweight; PBF, percentage body fat; SES, socioeconomic status; WC, waist circumference.

Table S5. Summary of meta-analysis fixed effect results for the various types of human studies

	Significance test of ES = 0		
**Comparison**	**Effect size**	**95% CI**	**P**
Observational studies	-0.03 kg/m2	-0.04 to -0.03	<0.001
Short interventions			
LES vs sugar (total energy)	-124 kcal	-133 to -115	<0.001
LES vs sugar (COMPX)	29.4%	25.0 to 33.8	<0.001
LES vs unsweetened	51.7 kcal	35.8 to 67.6	<0.001
LES vs water	-1.9 kcal	-29.7 to 25.8	0.892
LES vs nothing	20.9 kcal	-15.2 to 57.0	0.257
LES in capsules vs placebo capsules	-45.0 kcal	-75.3 to -14.7	0.004
Sustained interventions			
LES vs sugar	-0.81 kg	-1.07 to -0.56	<0.001
LES vs water	-1.36 kg	-2.04 to -0.69	<0.001

Table S6. Summary of results of Egger's regression test for funnel plot asymmetry for the various types of human studies

Comparison	Z statistic	P
Observational studies	1.57	0.12
Short interventions		
LES vs sugar (total energy)	0.92	0.36
LES vs sugar (COMPX)	2.21	0.03
LES vs unsweetened	0.49	0.62
LES vs water	0.38	0.71
LES vs nothing	-0.30	0.76
LES in capsules vs placebo capsules	0.62	0.53
Sustained interventions		
LES vs sugar	-0.35	0.73
LES vs water	0.74	0.46

Table S7. Characteristics and results of short-term intervention studies comparing the effects on EI of LES versus sugar

Children <br>**Reference**	**Sample size**	**Gender, age (y)** <br>**(mean, SD/SEM** <br>**or range)**	**Weight status, restraint status**	**LES and comparison preloads**	**Preload to test meal interval (min)**	**Intake test meal (kcal), LES vs comparison**	**Δ cumulative intake (kcal),** <br>**LES minus comparison (kcal)**	**COMPX (energy compen sation %)**	**Notes**
Anderson et al.[95] (Exp. 2)	20	10 M / 10 F, 10 y	Mean weight 30 kg X	300 ml strawberry Kool-Aid with 0.3 g aspartame (5 kJ, 1 kcal) or 52.5 g sucrose (892 kJ, 210 kcal)	90	777 vs 765	-197	6%	
Birch et al.[96] (Exp. 1)	24	10 M / 14 F, 5 y	X X	205 mL water with 140 mg aspartame (15 kJ, 3.5 kcal) or 22 g sucrose (376 kJ, 90 kcal)	0	451 vs 397	-32	62%	
Birch et al.[96] (Exp. 1)	24	10 M / 14 F, 5 y	X X	205 mL water with 140 mg aspartame (15 kJ, 3.5 kcal) or 22 g sucrose (376 kJ, 90 kcal)	30	458 vs 459	-87.5	-1%	
Birch et al.[96] (Exp. 1)	24	10 M / 14 F, 5 y	X X	205 mL water with 140 mg aspartame (15 kJ, 3.5 kcal) or 22 g sucrose (376 kJ, 90 kcal)	60	378 vs 388	-96.5	-12%	
Birch et al.[96] (Exp. 2)	20	7 M / 13 F, 3 y	X X	150 mL water with 102 mg aspartame (11 kJ, 2.6 kcal) or 16 g sucrose (276 kJ, 66 kcal)	0	350 vs 290	-3.4	95%	
Birch et	20	7 M / 13 F,	X	150 mL water with 102 mg	30	353 vs 300	-10.4	84%	

Children<br><br>**Reference**	**Sample size**	**Gender, age (y)**<br><br>**(mean, SD/SEM**<br><br>**or range)**	**Weight status, restraint status**	**LES and comparison preloads**	**Preload to test meal interval (min)**	**Intake test meal (kcal), LES vs comparison**	**Δ cumulative intake (kcal),**<br><br>**LES minus comparison (kcal)**	**COMPX (energy compen sation %)**	**Notes**
al.[96]<br>(Exp. 2)		3 y	X	aspartame (11 kJ, 2.6 kcal) or 16 g sucrose (276 kJ, 66 kcal)					
Birch et al.[96]<br>(Exp. 2)	20	7 M / 13 F,<br>3 y	X<br>X	150 mL water with 102 mg aspartame (11 kJ, 2.6 kcal) or 16 g sucrose (276 kJ, 66 kcal)	60	346 vs 317	-34.4	46%	
Gheller et al.[97]	22	22 M / 0 F<br>12 y	OW and OB	250 mL orange Kool-Aid with 150 mg sucralose (0 kJ, 0 kcal) or 50 g glucose (837 kJ, 200 kcal)	30	1093 vs 1021	-128	36%	No video-game playing during preload to test meal interval
Gheller et al.[97]	22	22 M / 0 F<br>12 y	OW and OB	250 mL orange Kool-Aid with 150 mg sucralose (0 kJ, 0 kcal) or 50 g glucose (837 kJ, 200 kcal)	30	1187 vs 1054	-67	67%	Video-game playing during preload to test meal interval
Branton et al.[98]	19	19 M / 0 F<br>12 y	NW	250 mL orange Kool-Aid with 150 mg sucralose (0 kJ, 0 kcal) or 50 g glucose (837 kJ, 200 kcal)	30	977 vs 770	7	104%	No video-game playing during preload to test meal interval

Children<br><br>**Reference**	**Sample size**	**Gender, age (y)**<br>**(mean, SD/SEM**<br>**or range)**	**Weight status, restraint status**	**LES and comparison preloads**	**Preload to test meal interval (min)**	**Intake test meal (kcal), LES vs comparison**	**Δ cumulative intake (kcal),**<br>**LES minus comparison (kcal)**	**COMPX (energy compen sation %)**	**Notes**
Branton et al.[98]	19	19 M / 0 F<br>12 y	NW	250 mL orange Kool-Aid with 150 mg sucralose (0 kJ, 0 kcal) or 50 g glucose (837 kJ, 200 kcal)	30	881 vs 749	68	66%	
Van Engelen et al.[99] (Exp. 1)	15	15 M / 0 F<br>12 y	NW	250 mL orange Kool-Aid with 150 mg sucralose (0 kJ, 0 kcal) or 50 g glucose (837 kJ, 200 kcal)	60	1127 vs 975	-48	76%	
Van Engelen et al.[99] (Exp. 1)	15	15 M / 0 F<br>12 y	NW	250 ml orange Kool-Aid with 150 mg sucralose (0 kJ, 0 kcal) or 50 g sucrose (837 kJ, 200 kcal)	60	1127 vs 1074	-147	27%	
Van Engelen et al.[99] (Exp. 1)	15	15 M / 0 F<br>12 y	NW	250 ml orange Kool-Aid with 150 mg sucralose (0 kJ, 0 kcal) or 50 g HFCS-55 (837 kJ, 200 kcal)	60	1127 vs 1075	-148	26%	
Hetherington et al.[100] (Exp. 1)	15	7 M / 8 F,<br>2-5 y	X<br>X	100 g raspberry dessert with 0.025 g aspartame (25 kJ, 6 kcal) or 17.2 g sucrose (305 kJ, 73 kcal)	120	572 vs 488	17	125%	
Hetherington et al.[100] (Exp. 1)	10	5 M / 5 F,<br>7-10 y	X<br>X	100 g rasbberry dessert with 0.025 g aspartame (25 kJ, 6 kcal) or 17.2 g sucrose (305 kJ, 73 kcal)	120	549 vs 645	-163	-143%	

Children <br>**Reference**	**Sample size**	**Gender, age (y)** <br>**(mean, SD/SEM** <br>**or range)**	**Weight status, restraint status**	**LES and comparison preloads**	**Preload to test meal interval (min)**	**Intake test meal (kcal), LES vs comparison**	**Δ cumulative intake (kcal),** <br>**LES minus comparison (kcal)**	**COMPX (energy compen sation %)**	**Notes**
Hetherington et al.[100] (Exp. 2)	19	10 M / 9 F, 2-5 y	X X	150 g rasbberry dessert with 0.037 g aspartame (25 kJ, 9 kcal) or 25.8 g sucrose (458 kJ, 109 kcal)	120	480 vs 410	-30	70%	
Hetherington et al.[100] (Exp. 2)	12	6 M / 6 F, 7-10 y	X X	225 g raspberry dessert with 0.056 g aspartame (56 kJ, 13.5 kcal) or 38.7 g sucrose (686 kJ, 164 kcal)	120	430 vs 395	-116	23%	
Wilson et al.[101]	135	63 M / 72 F, 1.5-5.5 y	NW X	1.1 L chocolate milk with aspartame (345 kJ, 83 kcal) or 1.3 L chocolate milk with sucrose (560 kJ, 134 kcal) - with macaroni (meal #1)	0	242 vs 252	-60	-18%	each condition was conducted twice
Wilson et al.[101]	135	63 M / 72 F, 1.5-5.5 y	NW X	1.5 L chocolate milk with aspartame (389 kJ, 93 kcal) or 1.3 L chocolate milk with sucrose (581 kJ, 139 kcal) - with scrambled eggs (meal #2)	0	206 vs 214	-55	-20%	each condition was conducted twice

Children <br>**Reference**	**Sample size**	**Gender, age (y)** <br>**(mean, SD/SEM** <br>**or range)**	**Weight status, restraint status**	**LES and comparison preloads**	**Preload to test meal interval (min)**	**Intake test meal (kcal), LES vs comparison**	**∆ cumulative intake (kcal),** <br>**LES minus comparison (kcal)**	**COMPX (energy compen sation %)**	**Notes**
Wilson et al.[101]	135	63 M / 72 F, 1.5-5.5 y	NW X	1.1 L chocolate milk with aspartame (345 kJ, 83 kcal) or 1.3 L chocolate milk with sucrose (581 kJ, 139 kcal) - with spaghetti and meat sauce (meal #3)	0	204 vs 209	-51	9%	each condition was conducted twice x
Wilson et al.[101]	135	63 M / 72 F, 1.5-5.5 y	NW X	1.5 L chocolate milk with aspartame (389 kJ, 93 kcal) or 1.5 L chocolate milk with sucrose (648 kJ, 155 kcal) - with grilled cheese sandwich (meal #4)	0	241 vs 248	-55	11%	each condition was conducted twice
Bellissimo et al.[102]	14	14 M / 0 F, 9-14 y	11 NW/ 1 OW/ 2 OB Non-dieting	250 mL water with sucralose (0 kJ) matched for sweetness with 1.0g/kg BW glucose (mean 836 kJ, 200 kcal)	30	1332 vs 1097	35	118%	'No TV'-condition only
Bellissimo et al.[103]	14	14 M / 0 F, 9-14 y	14 NW Non-dieting	250 mL water with sucralose (0 kJ) matched for sweetness with 1.0g/kg BW glucose (mean 836 kJ, 200 kcal)	30	1082 vs 893	-11.2	95%	

Children Reference	Sample size	Gender, age (y) (mean, SD/SEM or range)	Weight status, restraint status	LES and comparison preloads	Preload to test meal interval (min)	Intake test meal (kcal), LES vs comparison	Δ cumulative intake (kcal), LES minus comparison (kcal)	COMPX (energy compensation %)	Notes
Patel et al.[(104)]	29	29 M / 0 F, 9-14 y	Mean weight 51 kg Non-dieting	250 mL water with sucralose (0 kcal) matched for sweetness with 0.75g/kg BW glucose (640 kJ, 153 kcal)	30	1008 vs 848	7	105%	
Patel et al.[(104)]	29	0 M / 29 F, 9-14 y	Mean weight 51 kg Non-dieting	250 mL water with sucralose (0 kcal) matched for sweetness with 0.75g/kg BW glucose (640 kJ, 153 kcal)	30	841 vs 662	26	117%	
Patel et al.[(104)]	29	29 M / 0 F, 9-14 y	Mean weight 51 kg Non-dieting	250 mL water with sucralose (0 kcal) matched for sweetness with 0.75g/kg BW glucose (640 kJ, 153 kcal)	60	889 vs 927	-191	-25%	
Patel et al.[(104)]	29	0 M / 29 F, 9-14 y	Mean weight 51 kg Non-dieting	250 mL water with sucralose (0 kcal) matched for sweetness with 0.75g/kg BW glucose (640 kJ, 153 kcal)	60	765 vs 695	-83	46%	

Children Reference	Sample size	Gender, age (y) (mean, SD/SEM or range)	Weight status, restraint status	LES and comparison preloads	Preload to test meal interval (min)	Intake test meal (kcal), LES vs comparison	Δ cumulative intake (kcal), LES minus comparison (kcal)	COMPX (energy compen sation %)	Notes
Patel et al.[104]	25	0 M / 25 F, 9-14 y	21 NW/ 3 OW/ 1 OB Non-dieting	250 mL water with sucralose (0 kcal) matched for sweetness with 1.0 g/kg BW glucose (706 kJ, 169 kcal)	30	940 vs 779	-8	95%	
Tamam et al.[105] (Exp. 1)	18	18 M / 0 F, 9-14 y	NW Non-dieting	250 mL water with sucralose (0 kcal) matched for sweetness with 1.0 g/kg BW glucose (744 kJ, 178 kcal)	30	910 vs 763	-31	83%	Sedentary condition
Tamam et al.[105] (Exp. 1)	17	17 M / 0 F, 9-14 y	OW and OB Non-dieting	250 mL water with sucralose (0 kcal) matched for sweetness with 1.0 g/kg BW glucose (853 kJ, 204 kcal)	30	1182 vs 994	10	106%	Sedentary condition
Tamam et al.[105] (Exp. 2)	19	19 M / 0 F, 9-14 y	NW Non-dieting	250 mL water with sucralose (0 kcal) matched for sweetness with 1.0 g/kg BW glucose (782 kJ, 187 kcal)	30	1064 vs 807	70	137%	
Booth et al.[106]	12	11 M / 1 F, X	X X	100 ml drink with 50 g glucose (kJ, 184 kcal) or with saccharin and cyclamate (0 kJ, 0 kcal)	0	352 vs 295	-143	29%	

Children<br><br>Reference	Sample size	Gender, age (y)<br><br>(mean, SD/SEM<br><br>or range)	Weight status, restraint status	LES and comparison preloads	Preload to test meal interval (min)	Intake test meal (kcal), LES vs comparison	Δ cumulative intake (kcal),<br><br>LES minus comparison (kcal)	COMPX (energy compen sation %)	Notes
Booth et al.[106]	12	11 M / 1 F,<br>X	X<br>X	100 ml drink with 50 g glucose (kJ, 184 kcal) or with saccharin and cyclamate (0 kJ, 0 kcal)	20	322 vs 211	-89	56%	
Booth et al.[106]	12	11 M / 1 F,<br>X	X<br>X	100 ml drink with 50 g glucose (kJ, 184 kcal) or with saccharin and cyclamate (0 kJ, 0 kcal)	180	201 vs 158	-157	22%	
Brala & Hagen[107]	34	M/ F (not specified, about half-half), undergraduate students	NW<br>X	240 ml chocolate milk with 6 g aspartame (828 kJ, 198 kcal) or 60 g glucose (1795kJ, 429 kcal)	75	643 vs 400	12	105%	Rinse with sweetness blocker (gymnemic acid)<br><br>n=16 sucrose, n=18 aspartame

Adults <br>**Reference**	**Sample size**	**Gender, age (y) (mean, SD/SEM or range)**	**Weight status, restraint status**	**LES and comparison preloads**	**Preload to test meal interval (min)**	**Intake test meal (kcal), LES vs comparison**	**Δ intake preload + test meal (kcal), LES minus comparison (kcal)**	**COMPX (energy compen sation %)**	**Notes**
Brala & Hagen[107]	34	M/ F (not specified, about half-half), undergraduate students	NW X	240 ml chocolate milk with 6 g aspartame (828kJ, 198 kcal) or 60 g glucose (1795 kJ, 429 kcal)	75	623 vs 595	-203	12%	Rinse with tea <br>n=17 sucrose, n=17 aspartame
Rogers et al.[108]	33	25 M / 8 F, 19 y	NW NR	200 ml of tap water with 240 mg ace-K (0 kJ) or 50 g glucose (786 kJ, 188 kcal)	60	1395 vs 1271	-64	66%	
Rogers et al.[108]	33	25 M / 8 F, 19 y	NW NR	200 ml of tap water with 145 mg sacharine (o kJ) or 50 g glucose (786 kJ, 188 kcal)	60	1388 vs 1271	-71	62%	
Rogers et al.[108]	33	25 M / 8 F, 19 y	NW NR	200 ml of tap water with 162 mg aspartame (13 kJ, 3 kcal) or 50 g glucose (786 kJ, 188 kcal)	60	1333 vs 1271	-123	33%	
Rogers & Blundell[109]	21	4 M / 17 F, 18-29 y	NW NR	Yogurt with saccharin (548 kJ, 131 kcal) or glucose (1234 kJ, 295	60	949 vs 741	44	127%	

Adults<br><br>Reference	Sample size	Gender, age (y)<br><br>(mean, SD/SEM<br><br>or range)	Weight status, restraint status	LES and comparison preloads	Preload to test meal interval (min)	Intake test meal (kcal), LES vs comparison	Δ intake preload + test meal (kcal),<br><br>LES minus comparison (kcal)	COMPX (energy compen sation %)	Notes
				kcal)					
Rolls et al.(110)	16	8 M / 8 F, 18-35 y	NW NR	347 g of jello with aspartame (145 kJ, 35 kcal) or 318 g jello with sucrose (799 kJ, 191 kcal)	120	1103 vs 1062	-115	26%	Participants informed about preload energy content
Rolls et al.(110)	16	8 M / 8 F, 18-35 y	NW NR	389 g of chocolate pudding with aspartame (1628 kJ, 389 kcal) or 344 g of chocolate pudding with sucrose (2303 kJ, 551 kcal)	120	986 vs 856	-32	80%	Participants informed about preload energy content
Rolls et al.(110)	16	8 M / 8 F, 18-35 y	NW NR	287 g of jello with aspartame (121 kJ, 29 kcal) or 294 g jello with sucrose (739 kJ, 177 kcal)	120	1057 vs 938	-29	80%	Participants not informed about preload energy content.
Rolls et	16	8 M / 8 F,	NW	289 g of chocolate pudding with	120	910 vs 816	-112	46%	Participants not informed

Adults<br><br>Reference	Sample size	Gender, age (y)<br><br>(mean, SD/SEM<br><br>or range)	Weight status, restraint status	LES and comparison preloads	Preload to test meal interval (min)	Intake test meal (kcal), LES vs comparison	∆ intake preload + test meal (kcal),<br><br>LES minus comparison (kcal)	COMPX (energy compen sation %)	Notes
al.[110]		18-35 y	NR	asapartame (1209 kJ, 289 kcal) or 310 g of chocolate pudding with sucrose (2072 kJ, 495 kcal)					about preload energy content.
Rodin et al.[111]	6	6 M / 0 F, 22-50 y	OW<br>X	500 ml of lemon flavored water with 0.25 g of aspartame (15 kJ, 3.7 kcal) or 50 g of fructose (854 kJ, 204 kcal)	50	1597 vs 1191	206	203%	
Rodin et al.[111]	6	6 M / 0 F, 22-50 y	OW<br>X	500 ml of lemon flavored water with 0.25 g of aspartame (15 kJ, 3.7 kcal) or 50 g of glucose (854 kJ, 204 kcal)	50	1597 vs 1267	130	168%	
Rodin et al.[111]	4	4 M / 0 F, 22-50 y	NW<br>X	500 ml of lemon flavored water with 0.25 g of aspartame (15 kJ, 3.7 kcal) or 50 g of fructose (854 kJ, 204 kcal)	50	1217 vs 794	223	211%	
Rodin et al.[111]	4	4 M / 0 F, 22-50 y	NW<br>X	500 ml of lemon flavored water with 0.25 g of aspartame (15 kJ, 3.7 kcal) or 50 g of glucose	50	1217 vs 1142	-125	37%	

Adults<br><br>Reference	Sample size	Gender, age (y)<br><br>(mean, SD/SEM<br><br>or range)	Weight status, restraint status	LES and comparison preloads	Preload to test meal interval (min)	Intake test meal (kcal), LES vs comparison	∆ intake preload + test meal (kcal),<br><br>LES minus comparison (kcal)	COMPX (energy compen sation %)	Notes
				(854 kJ, 204 kcal)					
Rodin et al.[111]	6	0 M / 6 F, 22-50 y	OW<br>X	500 ml of lemon flavored water with 0.25 g of aspartame (15 kJ, 3.7 kcal) or 50 g of fructose (854 kJ, 204 kcal)	50	1204 vs 854	150	175%	
Rodin et al.[111]	6	0 M / 6 F, 22-50 y	OW<br>X	500 ml of lemon flavored water with 0.25 g of aspartame (15 kJ, 3.7 kcal) or 50 g of glucose (854 kJ, 204 kcal)	50	1204 vs 1150	-146	27%	
Rodin et al.[111]	4	4 M / 0 F, 22-50 y	NW<br>X	500 ml of lemon flavored water with 0.25 g of aspartame (15 kJ, 3.7 kcal) or 50 g of fructose (854 kJ, 204 kcal)	50	978 vs 813	-35	83%	
Rodin et al.[111]	4	4 M / 0 F, 22-50 y	NW<br>X	500 ml of lemon flavored water with 0.25 g of aspartame (15 kJ, 3.7 kcal) or 50 g of glucose (854 kJ, 204 kcal)	50	978 vs 916	-138	31%	

Adults Reference	Sample size	Gender, age (y) (mean, SD/SEM or range)	Weight status, restraint status	LES and comparison preloads	Preload to test meal interval (min)	Intake test meal (kcal), LES vs comparison	∆ intake preload + test meal (kcal), LES minus comparison (kcal)	COMPX (energy compen sation %)	Notes
Rogers et al.[112]	18	M / F (not specified), 18-25	NW, non-dieting	Yogurt with 200 mg aspartame (548 kJ, 131 kcal) or 50 g glucose (1233 kJ, 295 kcal)	60	878 vs 734	-20	88%	Subjects not informed about sweetener or kcal content of yogurt
Rogers et al.[112]	23	M / F (not specified), 18-25	NW, non-dieting	Yogurt with 200 mg aspartame (548 kJ, 131 kcal) or 50 g glucose (1233 kJ, 295 kcal)	60	1130 vs 974	-8	95%	Subjects informed about sweetener or kcal content of yogurt
Rolls et al.[113]	13	13 M / 0 F, 25 (4) y	NW Non-dieting	237 ml drink with 0.11 g aspartame (21 kJ, 5 kcal) or sucrose (347 kJ, 83 kcal)	0	1022 vs 1138	-194	149%	Preload consumed with the meal
Rolls et al.[113]	13	13 M / 0 F, 25 (4) y	NW Non-dieting	473 ml drink 0.22 gaspartame (42 kJ, 10 kcal) or sucrose (694 kJ, 166 kcal)	0	1113 vs 1046	-89	43%	Preload consumed with the meal
Rolls et	13	13 M / 0 F,	NW Non-	237 ml drink with 0.11 g aspartame (21 kJ, 5	30	1093 vs	-83	-6%	

Adults **Reference**	**Sample size**	**Gender, age (y) (mean, SD/SEM or range)**	**Weight status, restraint status**	**LES and comparison preloads**	**Preload to test meal interval (min)**	**Intake test meal (kcal), LES vs comparison**	**Δ intake preload + test meal (kcal), LES minus comparison (kcal)**	**COMPX (energy compen sation %)**	**Notes**
al.[113]		25 (4) y	dieting	kcal) or sucrose (347 kJ, 83 kcal)		1098			
Rolls et al.[113]	13	13 M / 0 F, 25 (4) y	NW Non-dieting	473 ml drink 0.22 gaspartame (42 kJ, 10 kcal) or sucrose (694 kJ, 166 kcal)	30	1138 vs 1096	-114	27%	
Rolls et al.[113]	13	13 M / 0 F, 25 (4) y	NW Non-dieting	237 ml drink with 0.11 g aspartame (21 kJ, 5 kcal) or sucrose (347 kJ, 83 kcal)	60	1211 vs 1104	-29	137%	
Rolls et al.[113]	13	13 M / 0 F, 25 (4) y	NW Non-dieting	473 ml drink 0.22 gaspartame (42 kJ, 10 kcal) or sucrose (694 kJ, 166 kcal)	60	1140 vs 1134	-150	4%	
Canty & Chan[114]	20	20 M / 0 F, 29 (1) y, 23-37 y	NW NR	200 ml of cherry flavored drink with 112 mg aspartame (not reported; 0 kcal?) or 20 g of sucrose (not reported; 80 kcal?)	60	606 vs 504	22	128%	

Adults<br><br>Reference	Sample size	Gender, age (y)<br><br>(mean, SD/SEM<br><br>or range)	Weight status, restraint status	LES and comparison preloads	Preload to test meal interval (min)	Intake test meal (kcal), LES vs comparison	Δ intake preload + test meal (kcal),<br><br>LES minus comparison (kcal)	COMPX (energy compen sation %)	Notes
Canty & Chan[114]	20	20 M / 0 F, 29 (1) y, 23-37 y	NW NR	200 ml of cherry flavored drink with 67.5 mg saccharine (energy not reported) or 20 g of sucrose (energy not reported)	60	568 vs 504	-16	80%	
Drewnowski et al.[115]	12	0 M 12 F, 34 y	OB R	500 g of cream cheese with aspartame (1255 kJ, 300 kcal) or aspartame + maltodextrin (2929 kJ, 700 kcal)	180	1255 vs 1180	-325	19%	
Drewnowski et al.[116]	12	0 M / 12 F, 25 y	NW NR	500 g of cream cheese with aspartame (1255 kJ, 300 kcal) or sucrose (2929 kJ, 700 kcal)	180	584 vs 535	-351	12%	
Drewnowski et al.[116]	12	12 M / 0 F, 26 y	NW NR	500 g of cream cheese with aspartame (1255 kJ, 300 kcal) or sucrose (2929 kJ, 700 kcal)	0	900 vs 817	-317	21%	
Guss et al.[117]	8	0 M / 8 F, 20 y	NW X	500 ml lemon flavored drink with fructose 1% + 250 mg aspartame (88 kJ, 21 kcal) or with	30	679 vs 536	-36	80%	

Adults <br>**Reference**	**Sample size**	**Gender, age (y)** <br>**(mean, SD/SEM** <br>**or range)**	**Weight status, restraint status**	**LES and comparison preloads**	**Preload to test meal interval (min)**	**Intake test meal (kcal), LES vs comparison**	**∆ intake preload + test meal (kcal),** <br>**LES minus comparison (kcal)**	**COMPX (energy compen sation %)**	**Notes**
				fructose 10% (837 kJ, 200 kcal)					
Guss et al.[(117)]	8	0 M / 8 F, 22 y	NW X	500 ml lemon flavored drink with fructose 1% + 250 mg aspartame (88 kJ, 21 kcal) or with fructose 10% (837 kJ, 200 kcal)	135	580 vs 524	-123	31%	
Guss et al.[(117)]	8	0 M / 8 F, 20 y	NW X	500 ml lemon flavored drink with glucose 1% + 250 mg aspartame (88 kJ, 21 kcal) or with fructose 10% (837 kJ, 200 kcal)	30	674 vs 519	-24	87%	
Guss et al.[(117)]	8	0 M / 8 F, 22 y	NW X	500 ml lemon flavored drink with fructose 1% + 250 mg aspartame (88 kJ, 21 kcal) or with glucose 10% (837 kJ, 200 kcal)	135	520 vs 570	-229	-28%	

Adults<br><br>**Reference**	**Sample size**	**Gender, age (y)**<br><br>**(mean, SD/SEM**<br><br>**or range)**	**Weight status, restraint status**	**LES and comparison preloads**	**Preload to test meal interval (min)**	**Intake test meal (kcal), LES vs comparison**	**Δ intake preload + test meal (kcal),**<br><br>**LES minus comparison (kcal)**	**COMPX (energy compen sation %)**	**Notes**
Reid and Hammersley (118)	18	18 M / 0 F, 18-55 y	NW<br>X	568 ml of orange squash with saccharin (42 kJ, 10 kcal) or 40 g sucrose (669 kJ, 160 kcal)	≥60, varied, next meal was not fixed in time	560 vs 614	-204	-36%	Between subjects design<br><br>Data recorded in diet diary
Reid and Hammersley (118)	11	0 M / 11 F, 18-55 y	NW<br>X	568 ml of orange squash with saccharin (42 kJ, 10 kcal) or 40 g sucrose (669 kJ, 160 kcal)	≥60, varied, next meal was not fixed in time	397 vs 262	-15	90%	Between subjects design. Data recorded in diet diary
Kim & Kissileff(119)	8	3 M / 5 F, 22 (2) y	NW<br>NR	500 ml of 1%-glucose solution with 260 mg aspartame (84 kJ, 20kcal) vs 15%-glucose (1254 kJ, 300kcal)	30	613 vs 465	-132	53%	
Kim & Kissileff(119)	8	3 M / 5 F, 22 (2) y	NW<br>NR	500 ml of 1%-glucose solution with 260 mg aspartame (84 kJ, 20kcal) vs 15%-glucose (1254 kJ, 300kcal)	30	668 vs 517	-129	54%	

Adults Reference	Sample size	Gender, age (y) (mean, SD/SEM or range)	Weight status, restraint status	LES and comparison preloads	Preload to test meal interval (min)	Intake test meal (kcal), LES vs comparison	Δ intake preload + test meal (kcal), LES minus comparison (kcal)	COMPX (energy compen sation %)	Notes
Lavin et al.[5]	14	0 M / 14 F, students	NW R, non-dieting	4*330 ml lemonade with aspartame (42 kJ or 10 kcal/1320 ml) or sucrose (1381 kJ/1320 ml, 330 kcal/1320 ml)	Intake during 24h	3181 vs, 2775	86	127%	Beverages served at 09.30, 11.30, 14.00 and 16.00
Beridot – Therond et al.[4]	24	12 M / 12 F, 20-25 y	NW NR	876 ml orange-flavored beverage with aspartame (0 kJ) or 686 ml beverage with sucrose (1296 kJ, 310 kcal)	0 to 15	803 vs 782	-289	7%	intake continued up to and during lunch
Beridot – Therond et al.[4]	24	12 M / 12 F, 20-25 y	NW NR	541 ml orange-flavored beverage with aspartame (0 kJ, 0 kcal) or 658 ml beverage with sucrose (1099 kJ, 263 kcal)	0 to 360, continuous ad lib access from end of lunch intill diner	595 vs 627	-295	-12%	intake continued up to and during diner
King et al.[120]	16	16 M / 0 F, 21 y	NW NR	Ad libitum drink with aspartame /ace-K (50 kJ, 12) or sucrose (971 kJ, 232)	10	1520 vs 1331	-31	86%	Preload after exercise
Melanson et al.[121]	10	10 M / 0 F, 25 y	NW X	350 g of lemon flavored drink with aspartame (36 kcal) or sucrose (239 kcal)	Not fixed	1401 vs 1460	-262	-29%	

Adults <br>**Reference**	**Sample size**	**Gender, age (y)** <br>**(mean, SD/SEM** <br>**or range)**	**Weight status, restraint status**	**LES and comparison preloads**	**Preload to test meal interval (min)**	**Intake test meal (kcal), LES vs comparison**	**∆ intake preload + test meal (kcal),** <br>**LES minus comparison (kcal)**	**COMPX (energy compen sation %)**	**Notes**
Reid and Hammersley [122]	20	20 M / 0 F, 31 y	NW X	80 g of low fat yogurt with sucrose (859 kJ, 205 kcal) or saccharin (230 kJ, 55 kcal)	≥0, varied, next meal was not fixed in time	1026 vs 688	551	232%	Between subjects design <br>Data recorded in diet diary
Reid and Hammersley [122]	20	0 M / 20 F, 30 y	NW X	80 g of low fat yogurt with sucrose (859 kJ, 205 kcal) or saccharin (230 kJ, 55 kcal)	≥0, varied, next meal was not fixed in time	327 vs 305	-153	13%	Between subjects design. Data recorded in diet diary
Holt et al.[123]	11	11 M / 0 F, 22 (3) y	NW NR	375 ml of diet coca cola + 40 g crushed ice (7 kJ, 2 kcal) or regular coca cola (629 kJ, 150 kcal)	20	490 vs 502	-160	-8%	
Woodend & Anderson[124]	14	14 M / 0 F, 24 y	NW / OW Non-dieting	360 ml of beverage preloads with sucralose (0 kJ) or 25 g sucrose (418 kJ, 100kcal)	60	1066 vs978	-12	88%	
Woodend & Anderson[124]	14	14 M / 0 F, 24 y	NW / OW Non-dieting	360 ml of beverage preloads with sucralose (0 kJ, 0 kcal) or 50 g sucrose (836 kJ, 200 kcal)	60	1066 vs 978	-112	44%	

Adults<br><br>Reference	Sample size	Gender, age (y)<br><br>(mean, SD/SEM<br><br>or range)	Weight status, restraint status	LES and comparison preloads	Preload to test meal interval (min)	Intake test meal (kcal), LES vs comparison	Δ intake preload + test meal (kcal),<br><br>LES minus comparison (kcal)	COMPX (energy compen sation %)	Notes
Woodend & Anderson[124]	14	14 M / 0 F, 24 y	NW / OW Non-dieting	360 ml of beverage preloads with sucralose (0 kJ, 0 kcal) or 75 g sucrose (1254 kJ, 300 kcal)	60	1066 vs 831	-65	78%	
Van Wymelbeke et al.[2]	24	12 M / 12 F, 20-25 y	NW<br>X	Consumed total over day of 2 l of orange or raspberry flavored beverage with aspartame / saccharin / ace-K (0 kJ, 0 kcal) or sucrose (1672 kJ / l, 400 kcal / l)	24h intake	2057 vs 1928	-671	16%	
Delavalle et al.[125]	44	0 M / 44 F, 25 y	NW / OW<br>NR	360 g of diet cola (0 kJ, 0 kcal) or regular cola (653 kJ, 150 kcal)	0	893 vs. 795	-52	65%	
Akhavan & Anderson[30] (Exp. 1)	12	12 M/ 0 F, 29 y	NW<br>NR	300 ml water with lemon juice added with sucralose (0 kJ, 0 kcal) or fructose(80%)/ glucose(20%) mix (1254 kJ, 300 kcal)	(min)	1220 vs 1207	-287	4%	

Adults <br>**Reference**	**Sample size**	**Gender, age (y)** <br>**(mean, SD/SEM** <br>**or range)**	**Weight status, restraint status**	**LES and comparison preloads**	**Preload to test meal interval (min)**	**Intake test meal (kcal), LES vs comparison**	**Δ intake preload + test meal (kcal),** <br>**LES minus comparison (kcal)**	**COMPX (energy compen sation %)**	**Notes**
Akhavan & Anderson[30] (Exp. 1)	12	12 M/ 0 F, 29 y	NW NR	300 ml water with lemon juice added with sucralose (0 kJ, 0 kcal) or HFCS55 (1254 kJ, 300 kcal)	80	1220 vs 1132	-212	29%	
Akhavan & Anderson[30] (Exp. 1)	12	12 M/ 0 F, 29 y	NW NR	300 ml water with lemon juice added with sucralose (energy not reported) or sucrose (1254 kJ, 300 kcal)	80	1220 vs 1052	-132	56%	
Akhavan & Anderson[30] (Exp. 1)	12	12 M/ 0 F, 29 y	NW NR	300 ml water with lemon juice added with sucralose (0 kJ, 0 kcal) or glucose(80%)/ fructose(20%) mix (1254 kJ, 300 kcal)	80	1220 vs 1045	-126	58%	
Appleton & Blundell[3]	10	0 M/ 10 F, X	NW / OW NR, <br>low LES consumer	2 x 330 ml beverage with LES (41 kJ, 10 kcal) or sugars (1046 kJ, 250 kcal) before lunch	90 + 180	597 vs 584	-227	5%	test meal 180 min after 1st drink and 90 min after 2nd drink (morning)

Adults<br><br>Reference	Sample size	Gender, age (y)<br><br>(mean, SD/SEM<br><br>or range)	Weight status, restraint status	LES and comparison preloads	Preload to test meal interval (min)	Intake test meal (kcal), LES vs comparison	∆ intake preload + test meal (kcal),<br><br>LES minus comparison (kcal)	COMPX (energy compen sation %)	Notes
Appleton & Blundell[(3)]	10	0 M/ 10 F, X	OW<br>NR, high LES consumer s	2 x 330 ml beverage with LES (41 kJ, 10 kcal) or sugars (1046 kJ, 250 kcal) before lunch	90 + 180	906 vs 868	-202	16%	test meal 180 min after 1st drink and 90 min after 2nd drink (morning)
Appleton & Blundell[(3)]	10	0 M/ 10 F, X	NW / OW<br>NR,<br><br>low LES consumer s	2 x 330 ml beverage with LES (41 kJ, 10 kcal) or sugars (1046 kJ, 250 kcal) before evening meal	90 + 180	591 vs 540	-189	21%	test meal 180 min after 1st drink and 90 min after 2nd drink (afternoon)
Appleton & Blundell[(3)]	10	0 M/ 10 F, X	OW<br>NR, high LES consumer	2 x 330 ml beverage with LES (41 kJ, 10 kcal) or sugars (1046 kJ, 250 kcal) before evening meal	90 + 180	642 vs 643	-241	0%	test meal 180 min after 1st drink and 90 min after 2nd drink (afternoon)
Monsivais et al.[(126)]	37	19 M / 18 F<br>M: 23 (4.0) y<br>F: 23 (2.8) y	NW<br>NR	475 ml of cola with aspartame (8 kJ, 2 kcal) or HCFS42 (899 kJ, 215 kcal)	120	1009 vs 979	-182	14%	

Adults Reference	Sample size	Gender, age (y) (mean, SD/SEM or range)	Weight status, restraint status	LES and comparison preloads	Preload to test meal interval (min)	Intake test meal (kcal), LES vs comparison	∆ intake preload + test meal (kcal), LES minus comparison (kcal)	COMPX (energy compen sation %)	Notes
Monsivais et al.[126]	37	19 M / 18 F<br>M: 23 (4.0) y<br>F: 23 (2.8) y	NW<br>NR	475 ml of cola with aspartame (8 kJ, 2 kcal) or HCFS55 (899 kJ, 215 kcal)	120	1009 vs 969	-171	19%	
Monsivais et al.[126]	37	19 M / 18 F<br>M: 23 (4.0) y<br>F: 23 (2.8) y	NW<br>NR	475 ml of cola with aspartame (8 kJ, 2 kcal) or sucrose (899 kJ, 215 kcal)	120	1009 vs 957	-161	24%	
Soenen and Westerterp – Plantenga [127]	20	0 M / 20 F,<br>21 y	NW<br>NR	800 ml of orange flavored beverage with aspartame / ace-K / sodium cyclamate (2 kJ, 0.5 kcal) or sucrose (1500 kJ, 359 kcal)	50	548 vs 417	-227	36%	
Soenen and Westerterp-Plantenga [127]	20	0 M / 20 F,<br>21 y	NW<br>NR	800 ml of orange flavored beverage with aspartame / ace-K / sodium cyclamate (2 kJ, 0.5 kcal) or hfcs (1500 kJ, 359 kcal)	50	548 vs 448	-259	28%	

Adults<br><br>Reference	Sample size	Gender, age (y)<br><br>(mean, SD/SEM<br><br>or range)	Weight status, restraint status	LES and comparison preloads	Preload to test meal interval (min)	Intake test meal (kcal), LES vs comparison	∆ intake preload + test meal (kcal),<br><br>LES minus comparison (kcal)	COMPX (energy compen sation %)	Notes
Soenen and Westerterp-Plantenga [127]	20	20 M / 0 F, 22 y	NW<br>NR	800 ml of orange flavored beverage with aspartame / ace-K / sodium cyclamate (2 kJ, 0.5 kcal) or sucrose (1500 kJ, 359 kcal)	50	753 vs 567	-172	52%	
Soenen and Westerterp-Plantenga [127]	20	20 M / 0 F, 22 y	NW<br>NR	800 ml of orange flavored beverage with aspartame / ace-K / sodium cyclamate (2 kJ, 0.5 kcal) or hfcs (1500 kJ, 359 kcal)	50	753 vs 558	-163	54%	
Anton et al.[128]	31	M / F (not specified), 28 y	19 NW/ 12 OB<br>NR	400 g of crackers and cream cheese with stevia (1212 kJ, 290 kcal) or sucrose (2060 kJ, 493 kcal) before lunch	20	575 vs 554	-182	10%	
Anton et al.[128]	31	M / F (not specified), 28 y	19 NW/ 12 OB<br>NR	400 g of crackers and cream cheese with aspartame (1212 kJ, 290 kcal) or sucrose (2060 kJ, 493 kcal) before lunch	20	590 vs 554	-167	18%	

Adults <br>**Reference**	**Sample size**	**Gender, age (y)** <br>**(mean, SD/SEM** <br>**or range)**	**Weight status, restraint status**	**LES and comparison preloads**	**Preload to test meal interval (min)**	**Intake test meal (kcal), LES vs comparison**	**Δ intake preload + test meal (kcal),** <br>**LES minus comparison (kcal)**	**COMPX (energy compen sation %)**	**Notes**
Anton et al.[128]	31	M / F (not specified), 28 y	19 NW/ 12 OB NR	400 g of crackers and cream cheese with stevia (1212 kJ, 290 kcal) or sucrose (2060 kJ, 493 kcal) before dinner	20	624 vs, 548	-127	37%	
Anton et al.[128]	31	M / F (not specified), 28 y	19 NW/ 12 OB NR	400 g of crackers and cream cheese with aspartame (1212 kJ, 290 kcal) or sucrose (2060 kJ, 493 kcal) before dinner	20	618 vs 548	-133	34%	
Ranawana & Henry[129]	23	23 M / 0 F, 24 y	NW NR	325 ml of fruit drink with aspartame + ace-K (0 kJ) or 349 ml of fruit drink with sucrose (627 kJ, 150 kcal)	60	1207 vs 1045	12	108%	
Ranawana & Henry[129]	23	23 M / 0 F, 24 y	NW NR	325 ml of fruit drink with aspartame + ace-K (0 kJ) or 319 ml of orange juice (627 kJ, 150 kcal)	60	1207 vs 1033	24	116%	

Adults <br>**Reference**	**Sample size**	**Gender, age (y)** <br>**(mean, SD/SEM** <br>**or range)**	**Weight status, restraint status**	**LES and comparison preloads**	**Preload to test meal interval (min)**	**Intake test meal (kcal), LES vs comparison**	**Δ intake preload + test meal (kcal),** <br>**LES minus comparison (kcal)**	**COMPX (energy compen sation %)**	**Notes**
Ranawana & Henry[129]	24	0 M / 24 F, 24 y	NW NR	325 ml of fruit drink with aspartame + ace-K (0 kJ) or 349 ml of fruit drink with sucrose (627 kJ, 150 kcal)	60	786 vs 776	-140	7%	
Ranawana & Henry[129]	24	0 M / 24 F, 24 y	NW NR	325 ml of fruit drink with aspartame + ace-K (0 kJ) or 319 ml of orange juice (627 kJ, 150 kcal)	60	786 vs 701	-65	57%	
Akhavan et al.[130] (Exp. 2)		15 M/ 0 F, 19-28 y	NW NR	300 ml lemon-flavored water with 0.13 g sucralose and 6 g gelatin not set (84 kJ, 20 kcal) or 75 g sucrose and 6 g gelatin set (1339 kJ, 320 kcal)	60	15	-204	32%	
Akhavan et al.[130] (Exp. 2)	15	15 M/ 0 F, 19-28 y	NW NR	300 ml lemon-flavored water with 0.13 g sucralose and 6 g gelatin not set (84 kJ, 20 kcal) or 75 g sucrose and 6 g gelatin not set (1339 kJ, 320 kcal)	60	1465 vs 1360	-195	35%	

Adults Reference	Sample size	Gender, age (y) (mean, SD/SEM or range)	Weight status, restraint status	LES and comparison preloads	Preload to test meal interval (min)	Intake test meal (kcal), LES vs comparison	Δ intake preload + test meal (kcal), LES minus comparison (kcal)	COMPX (energy compensation %)	Notes
Akhavan et al.[(130)] (Exp. 2)	15	15 M/ 0 F, 19-28 y	NW NR	300 ml lemon-flavored water with 0.13 g sucralose and 6g gelatin not set (84 kJ, 20 kcal) or 75 g sucrose and 6 g gelatin not set (1272 kJ, 304 kcal) or glucose (50%)/ fructose (50%) (1339 kJ, 320 kcal)	60	1465 vs 1358	-193	36%	
Rogers et al.[(131)]	15	15 M / 0 F, 25 y	NW, NR	300 ml of blackcurrant juice with sucralose (8 kJ, 2 kcal) or sucrose (669 kJ, 160 kcal)	20	1294 vs 1198	-62	61%	
Rogers et al.[(131)]	18	0 M / 18 F, 25 y	NW, NR	300 ml of blackcurrant juice with sucralose (8 kJ, 2 kcal) or sucrose (669 kJ, 160 kcal)	20	827 vs 760	-91	42%	
Maersk et al.[(132)]	14	7 M / 7 F, 34 (9.2) y	OW / OB X	500 ml of diet cola with aspartame (7.5 kJ, 2 kcal) or regular cola with sucrose (900 kJ, 215 kcal)	240	1196 vs 1155	-172	20%	

Adults<br><br>**Reference**	**Sample size**	**Gender, age (y)**<br><br>**(mean, SD/SEM**<br><br>**or range)**	**Weight status, restraint status**	**LES and comparison preloads**	**Preload to test meal interval (min)**	**Intake test meal (kcal), LES vs comparison**	**∆ intake preload + test meal (kcal),**<br><br>**LES minus comparison (kcal)**	**COMPX (energy compen sation %)**	**Notes**
Carvalho et al.[(133)]	24	13 M / 11 F, 21.5 (2.33) y	NW Non-dieting	500 ml of pineapple soda with sucralose (92 kJ, 22 kcal) or sugar (920 kJ, 220 kcal)	150	1385 vs 1488	-301	-52%	
Panahi et al.[(134)]	29	15 M / 14 F, 22 y	NW NR	373 g of diet cola (0 kJ, 0 kcal) or 443 g of regular cola (815 kJ, 195 kcal)	0	926 vs 915	-184	6%	Beverages consumed *ad libitum* with meal; *ad libitum* intake diet vs. regular cola NS

EI, energy intake; LES, low energy sweetener; Δ, change (difference from baseline to last time point unless otherwise described); COMPX, compensation index.

Table S8. Characteristics and results of short-term intervention studies comparing the effects on EI of LES versus unsweetened products

Children Reference	Sample size	Gender, age (y) (mean, SD/SEM or range)	Weight status, restraint status	LES and comparison preloads	Preload to test meal interval (min)	Intake test meal (kcal), LES vs comparison	∆ cumulative intake (kcal), LES minus comparison (kcal)	Notes
Wilson et al.[102]	135	63 M / 72 F, 1.5-5.5 y	NW X	1.1 L chocolate milk with aspartame (345 kJ, 83 kcal) or 0.8 L plain milk (222 kJ, 53 kcal)	0	242 vs 262	10	
Wilson et al.[102]	135	63 M / 72 F, 1.5-5.5 y	NW X	1.5 L chocolate milk with aspartame (389 kJ, 93 kcal) or 0.8 L plain milk (205 kJ, 49 kcal)	0	206 vs 250	0	
Wilson et al.[102]	135	63 M / 72 F, 1.5-5.5 y	NW X	1.1 L chocolate milk with aspartame (345 kJ, 83 kcal) or 0.8 L plain milk (205 kJ, 49 kcal)	0	209 vs 219	24	
Wilson et al.[102]	135	63 M / 72 F, 1.5-5.5 y	NW X	1.5 L chocolate milk with aspartame (389 kJ, 93 kcal) or 0.8 L plain milk (275 kJ, 66 kcal)	0	248 vs 275	0	

Adults <br>**Reference**	**Sample size**	**Gender, age (y)** <br>**(mean, SD/SEM** <br>**or range)**	**Weight status, restraint status**	**LES and comparison preloads**	**Preload to test meal interval (min)**	**Intake test meal (kcal), LES vs comparison**	**Δ intake preload + test meal (kcal),** <br>**LES minus comparison (kcal)**	**Notes**
Brala & Hagen[108]	39	M/ F (not specified, about half-half), undergraduate students	NW X	240 ml chocolate milk with 0.6 g aspartame (828 kJ, 198 kcal) (n = 18) or nothing added (828 kJ, 198 kcal) (n=21)	75	634 vs 480	163	Rinse with sweetness blocker (gymnemic acid) <br>n=18 aspartame, n=21 nothing added
Brala & Hagen[108]	34	M/ F (not specified, about half-half), undergraduate students	NW X	240 ml chocolate milk with 0.6 g aspartame (828 kJ, 198 kcal) (n=17) or nothing added (828 kJ, 198 kcal) (n=17)	75	623 vs 678 kcal	-55	Rinse with tea <br>n=17 aspartame, n=17 nothing [illegible]
Rogers & Blundell[110]	21	4 M / 17 F, 18-29 y	NW NR	Yogurt with saccharin (548 kJ, 131 kcal) or unsweetened yougurt (548 kJ, 131 kcal)	60	949 vs 947	102	
Ho et al.[135]	8	0 M / 8 F 40 y	NW R	167 ml lemon flavored pudding with cyclamate (280 kJ, 67 kcal) or unsweetened (280 kJ, 67 kcal)	30	456 vs 545	-89	

Adults<br><br>Reference	Sample size	Gender, age (y)<br><br>(mean, SD/SEM<br><br>or range)	Weight status, restraint status	LES and comparison preloads	Preload to test meal interval (min)	Intake test meal (kcal), LES vs comparison	Δ intake preload + test meal (kcal),<br><br>LES minus comparison (kcal)	Notes
Ho et al.[135]	9	0 M / 9 F<br>49 y	OW<br>R	167 ml lemon flavored pudding with cyclamate (280 kJ, 67 kcal) or unsweetened (280 kJ, 67 kcal)	30	480 vs 498	-18	
Ho et al.[135]	7	0 M / 7 F<br>41 y	NW<br>NR	167 ml lemon flavored pudding with cyclamate (280 kJ, 67 kcal) or unsweetened (280 kJ, 67 kcal)	30	657 vs 567	90	
Ho et al.[135]	8	0 M / 8 F<br>40 y	NW<br>R	167 ml lemon flavored pudding with cyclamate (1644 kJ, 393 kcal) or unsweetened (1644 kJ, 393 kcal)	30	406 vs 461	-55	
Ho et al.[135]	9	0 M / 9 F<br>49 y	OW<br>R	167 ml lemon flavored pudding with cyclamate (1644 kJ, 393 kcal) or unsweetened (1644 kJ, 393 kcal)	30	403 vs 415	-12	
Ho et al.[135]	7	0 M / 7 F<br>41 y	NW<br>NR	167 ml lemon flavored pudding with cyclamate (1644 kJ, 393 kcal) or unsweetened (1644 kJ, 393 kcal)	30	534 vs 552	-18	

Adults<br><br>Reference	Sample size	Gender, age (y)<br><br>(mean, SD/SEM<br><br>or range)	Weight status, restraint status	LES and comparison preloads	Preload to test meal interval (min)	Intake test meal (kcal), LES vs comparison	∆ intake preload + test meal (kcal),<br><br>LES minus comparison (kcal)	Notes
Rogers et al.[112]	18	M / F (not specified), 18-25	NW non-dieting	Yogurt with 200 mg aspartame (548 kJ, 131 kcal) or unsweetened yogurt (548 kJ, 131 kcal)	60	878 vs 814	64	Subjects not informed about sweetener or kcal content
Rogers et al.[112]	23	M / F (not specified), 18-25	NW non-dieting	Yogurt with 200 mg aspartame (548 kJ, 131 kcal) or unsweetened yogurt (548 kJ, 131 kcal)	60	1130 vs 1017	113	Subjects informed about sweetener or kcal content
Drewnowski et al.[115]	12	0 M / 12 F 34 y	OB NR	400 g cream cheese with aspartame (1255 kJ, 300 kcal) or plain cream cheese (1255 kJ, 300 kcal)	180 min	1255 vs 1224	31	
Drewnowski et al.[116]	12	0 M / 12 F 25 y	NW NR	400 g cream cheese with aspartame (1255 kJ, 300 kcal) or plain cream cheese (1255 kJ, 300 kcal)	180 min	584 vs 657	-73	
Drewnowski et al.[116]	12	12 M / 0 F 26 y	NW NR	400 g cream cheese with aspartame (xx kJ, xx kcal) or plain cream cheese (xx kJ, xx kcal)	180	900 vs 868	34	

Adults <br>**Reference**	**Sample size**	**Gender, age (y) (mean, SD/SEM or range)**	**Weight status, restraint status**	**LES and comparison preloads**	**Preload to test meal interval (min)**	**Intake test meal (kcal), LES vs comparison**	**Δ intake preload + test meal (kcal), LES minus comparison (kcal)**	**Notes**
Beridot – Therond et al.[(4)]	24	12 M / 12 F 20-25 y	NW NR	876 ml orange beverage with aspartame (0 kJ, 0 kcal) or 572 ml unsweetened orange flavored beverage	Ad libitum, 0 to 15	803 vs 819	-16	Ad libitum intake; intake from lunch up until and during dinner
Beridot – Therond et al.[(4)]	24	12 M / 12 F 20-25 y	NW NR	541 ml orange flavored beverage with aspartame (0 kJ, 0 kcal) or 572 ml unsweetened orange flavored beverage	Ad libitum beverage intake from 6 h before dinner	595 vs 632	-37	Ad libitum intake; intake from lunch up until and during dinner
Akhavan et al.[(130)] (Exp. 1)	14	14 M / 0 F, 19-28 y	NW, NR	300 ml of orange-flavored water with 6 g gelatin not set and 0.13 g sucralose (0 kJ, 0 kcal) or orange-flavored water with 6 g gelatin not set	60	1273 vs 1373	-100	

EI, energy intake; LES, low energy sweetener; Δ, change (difference from baseline to last time point unless otherwise described).

Table S9. Characteristics and results of short-term intervention studies comparing the effects on EI of LES versus water

Children Reference	Sample size	Gender, age (y) (mean, SD/SEM or range)	Weight status, restraint status	LES and comparison preloads	Preload to test meal interval (min)	Intake test meal (kcal), LES vs comparison	Δ cumulative intake (kcal), LES minus comparison (kcal)	Notes
Birch et al.[(96)] (Exp. 1)	24	10 M / 14 F, 5 y	NW/OW, non-dieting	205 mL water with 140 mg aspartame (15 kJ, 3.5 kcal) or water	0	451 vs 454	0.5	
Birch et al.[(96)] (Exp. 1)	24	10 M / 14 F, 5 y	NW/OW, non-dieting	205 mL water with 140 mg aspartame (15 kJ, 3.5 kcal) or water	30	458 vs 521	-59.5	
Birch et al.[(96)] (Exp. 1)	24	10 M / 14 F, 5 y	NW/OW, non-dieting	205 mL water with 140 mg aspartame (15 kJ, 3.5 kcal) or water	60	378 vs 421	-39.5	
Birch et al.[(96)] (Exp. 2)	20	7 M / 13 F, 3 y	NW/OW, non-dieting	205 mL water with 140 mg aspartame (15 kJ, 3.5 kcal) or water	0	350 vs 371	-18.4	
Birch et al.[(96)] (Exp. 2)	20	7 M / 13 F, 3 y	NW/OW, non-dieting	205 mL water with 140 mg aspartame (15 kJ, 3.5 kcal) or water	30	353 vs 391	-35.4	
Birch et al.[(96)] (Exp. 2)	20	7 M / 13 F, 3 y	NW/OW, non-dieting	205 mL water with 140 mg aspartame (15 kJ, 3.5 kcal) or water	60	346 vs 367	-18.4	

Adults<br><br>Reference	Sample size	Gender, age (y)<br><br>(mean, SD/SEM<br><br>or range)	Weight status, restraint status	LES and comparison preloads	Preload to test meal interval (min)	Intake test meal (kcal), LES vs comparison	Δ intake preload + test meal (kcal),<br><br>LES minus comparison (kcal)	Notes
Rogers et al.[108]	12	4 M / 8 F<br>19 y	NW<br>NR	200 ml of water with acesulfame K (0 kcal) or water	60	1395 vs 1436	-41	
Rogers et al.[108]	12	4 M / 8 F<br>19 y	NW<br>NR	200 ml of water with saccharine (0 kcal) or water	60	1388 vs 1436	-48	
Rogers et al.[108]	12	4 M / 8 F<br>19 y	NW<br>NR	200 ml of water with aspartame (13 kJ, 3 kcal) or water	60	1333 vs 1436	-100	
Rodin et al.[111]	6	6 M / 0 F<br>22-50 y	OW<br>X	500 ml of xx with aspartame (15 kJ, 3.7 kcal) or water	50	1597 vs 1487	114	
Rodin et al.[111]	4	4 M / 0 F<br>22-50 y	NW<br>X	500 ml of xx with aspartame (15 kJ, 3.7 kcal) or water	50	1217 vs 1333	-112	
Rodin et al.[111]	6	0 M / 6 F<br>22-50 y	OW<br>X	500 ml of xx with aspartame (15 kJ, 3.7 kcal) or water	50	1204 vs 1285	-77	
Rodin et al.[111]	4	0 M / 4 F<br>22-50 y	NW<br>X	500 ml of xx with aspartame (15 kJ, 3.7 kcal) or water	50	978 vs 809	173	

Adults<br><br>Reference	Sample size	Gender, age (y)<br><br>(mean, SD/SEM<br><br>or range)	Weight status, restraint status	LES and comparison preloads	Preload to test meal interval (min)	Intake test meal (kcal), LES vs comparison	∆ intake preload + test meal (kcal),<br><br>LES minus comparison (kcal)	Notes
Rogers et al.[136] (Exp. 1)	12	6 M / 6 F<br>18-26 y	NW<br>NR	200 ml of water with 235 mg aspartame (21 kJ, 5 kcal) plus placebo capsule with 1.2 g corn flour (21 kJ, 5 kcal) or water with same placebo capsule (21 kJ, 5 kcal)	60	1214 vs 1263	-49	
Rogers et al.[136] (Exp 2)	15	5 M / 10 F<br>19-24 y	NW<br>NR	200 ml of water with aspartame (4 kJ, 1 kcal) plus placebo capsule with 235 mg corn flour (4 kJ, 1 kcal) or water with placebo capsule (4 kJ, 1 kcal)	60	1339 vs 1467	-128	
Rolls et al.[114]	13	13 M / 0 F<br>25 (4.3) y	NW<br>NR	237 ml drink with 0.11 g aspartame (21 kJ, 5 kcal) or water	0	1022 vs 1083	-56	
Rolls et al.[113]	13	13 M / 0 F<br>25 (4.3) y	NW<br>NR	473 ml drink 0.22 gaspartame (42 kJ, 10 kcal) or water	0	1113 vs 1077	41	
Rolls et al.[113]	13	13 M / 0 F<br>25 (4.3) y	NW<br>NR	237 ml drink with 0.11 g aspartame (21 kJ, 5 kcal) or water	30	1093 vs 1137	-39	

Adults <br>**Reference**	**Sample size**	**Gender, age (y)** <br>**(mean, SD/SEM** <br>**or range)**	**Weight status, restraint status**	**LES and comparison preloads**	**Preload to test meal interval (min)**	**Intake test meal (kcal), LES vs comparison**	**Δ intake preload + test meal (kcal),** <br>**LES minus comparison (kcal)**	**Notes**
Rolls et al.[113]	13	13 M / 0 F<br>25 (4.3) y	NW<br>NR	473 ml drink 0.22 gaspartame (42 kJ, 10 kcal) or water	30	1138 vs 1199	-56	
Rolls et al.[113]	13	13 M / 0 F<br>25 (4.3) y	NW<br>NR	237 ml drink with 0.11 g aspartame (21 kJ, 5 kcal) or water	60	1211 vs 1147	69	
Rolls et al.[113]	13	13 M / 0 F<br>25 (4.3) y	NW<br>NR	473 ml drink 0.22 gaspartame (42 kJ, 10 kcal) or water	60	1140 vs 1125	-80	
Black et al.[137]	7	7 M / 0 F<br>19-25 y	NW / OW<br>R	280 ml soda with aspartame (0 kcal) or water	60	1770 vs 1970	-200	Consumed in 2 min
Black et al.[137]	13	13 M / 0 F<br>19-25 y	NW / OW<br>NR	280 ml soda with aspartame (0 kcal) or water	60	1370 vs 1290	80	Consumed in 2 min
Black et al.[137]	7	7 M / 0 F<br>19-25 y	NW / OW<br>R	280 ml soda with aspartame (0 kcal) or water	60	1880 vs 1970	-90	Consumed in 10 min
Black et al.[137]	13	13 M / 0 F<br>19-25 y	NW / OW<br>NR	280 ml soda with aspartame (0 kcal) or water	60	1432 vs 1290	142	Consumed in 10 min

Adults<br><br>Reference	Sample size	Gender, age (y)<br><br>(mean, SD/SEM<br><br>or range)	Weight status, restraint status	LES and comparison preloads	Preload to test meal interval (min)	Intake test meal (kcal), LES vs comparison	∆ intake preload + test meal (kcal),<br><br>LES minus comparison (kcal)	Notes
Black et al.[137]	7	7 M / 0 F<br>19-25 y	NW / OW<br>R	560 ml soda with aspartame (0 kcal) or water	60	1740 vs 1970	-230	Consumed in 10 min
Black et al.[137]	13	13 M / 0 F<br>19-25 y	NW / OW<br>NR	560 ml soda with aspartame (0 kcal) or water	60	1456 vs 1290	166	Consumed in 10 min
Canty & Chan[115]	20	20 M / 0 F<br>29 (1 (23-37))	NW<br>NR	200 ml cherry flavored drink with aspartame (energie not reported) or water	60	606 vs 589	17	
Canty & Chan[114]	20	20 M / 0 F<br>29 (1) (23-37) y	NW<br>NR	200 ml cherry flavored drink with saccharine (energy not reported) or water	60	568 vs 589	-21	
Black et al.[138]	18	18 M / 0 F<br>19-25 y	NW<br>X	280 ml diet soda (0 kJ, 0 kcal) or water	60	1721 vs 1721	0	
Black et al.[138]	18	18 M / 0 F<br>19-25 y	NW<br>X	560 ml diet soda (0 kJ, 0kcal) or water	60	1827 vs 1711	116	
Reid and Hammersley[118]	18	18 M / 0 F<br>18-55 y	NW<br>X	568 ml of orange squash with saccharin (42 kJ, 10 kcal) or water	≥60, varied, next meal was not fixed in time	560 vs 698	-128	Between subjects design

Adults **Reference**	**Sample size**	**Gender, age (y) (mean, SD/SEM or range)**	**Weight status, restraint status**	**LES and comparison preloads**	**Preload to test meal interval (min)**	**Intake test meal (kcal), LES vs comparison**	**Δ intake preload + test meal (kcal), LES minus comparison (kcal)**	**Notes**
Reid and Hammersley [118]	20	0 M / 20 F 18-55 y	NW X	568 ml of orange squash with saccharin (42 kJ, 10 kcal) or water	≥60, varied, next meal was not fixed in time	397 vs 548	-141	Between subjects design
Kim & Kissilef[119]	8	3 M / 5 F 22 (2) y	NW NR	500 ml of 1%-glucose solution with 260 mg aspartame (84 kJ, 20kcal) or water	30	613 vs 641	-8	Lab-setting
Kim & Kissilef[119]	8	3 M / 5 F 22 (2) y	NW NR	500 ml of 1%-glucose solution with 260 mg aspartame (84 kJ, 20kcal) or water	30	668 vs 641	47	Cafetaria-setting
Lavin et al.[5]	14	0 M / 14 F X	NW R	4 * 330 ml lemonade with aspartame (42 kJ or 10 kcal/1320 ml) or water	24h intake	3181 vs 2967	224	4*330 ml lemonade consumed during day
Beridot – Therond et al.[4]	24	12 M / 12 F 20-25 y	NW NR	876 ml orange beverage with aspartame (0 kJ, 0 kcal) or 646 ml water	*Ad libitum*, 0 to 15	803 vs 857	-54	*Ad libitum* intake <br>intake continued up to and during lunch

Adults<br><br>Reference	Sample size	Gender, age (y)<br><br>(mean, SD/SEM<br><br>or range)	Weight status, restraint status	LES and comparison preloads	Preload to test meal interval (min)	Intake test meal (kcal), LES vs comparison	Δ intake preload + test meal (kcal),<br><br>LES minus comparison (kcal)	Notes
Beridot – Therond et al.[(4)]	24	12 M / 12 F<br>20-25 y	NW<br>NR	876 ml orange flavored beverage with aspartame (0 kJ, 0 kcal) or 1480 ml water	*Ad libitum*, 0 to 15	803 vs 969	-166	*Ad libitum* intake<br><br>intake continued up to and during lunch
Beridot-Therond et al.[(4)]	24	12 M / 12 F<br>20-25 y	NW<br>NR	541 ml orange flavored with aspartame beverage (0 kJ, 0 kcal) or 1457 ml water	*Ad libitum* beverage intake from 6 hr before dinner	595 vs 615	-20	*Ad libitum* intake<br><br>intake from lunch up until and during dinner
Beridot-Therond et al.[(4)]	24	12 M / 12 F<br>20-25 y	NW<br>NR	541 ml orange flavored beverage with aspartame (0 kJ, 0 kcal) or 578 ml water	*Ad libitum* beverage intake from 6 hr before dinner	595 vs 647	-52	*Ad libitum* intake<br><br>intake from lunch up until and during dinner
King et al.[(120)]	16	16 M / 0 F<br>21 y	NW<br>NR	*Ad libitum* drink with aspartame /ace-K (50 kJ, 12 kcal) or water	10	1520 vs 1358	174	
Holt et al.[(123)]	11	11 M / 0 F<br>22 (3) y	NW<br>NR	375 ml diet coca cola + 40 g crushed ice (7 kJ, 2 kcal) or water	20	490 vs 554	-62	

Adults<br><br>Reference	Sample size	Gender, age (y)<br><br>(mean, SD/SEM<br><br>or range)	Weight status, restraint status	LES and comparison preloads	Preload to test meal interval (min)	Intake test meal (kcal), LES vs comparison	Δ intake preload + test meal (kcal),<br><br>LES minus comparison (kcal)	Notes
Woodend & Anderson[124]	14	14 M / 0 F<br>24 y	NW / OW<br>NR	300 ml of xx with 125 mg sucralose (0 kJ, 0 kcal) or water	60	1066 vs 1101	-35	
Delavalle et al.[125]	44	0 M / 44 F<br>25 (20-56) y	NW / OW<br>NR	360 ml diet coke (0 kJ, 0 kcal) or water	0 min	795 vs 794	1	
Akhavan & Anderson[30] (Exp. 1)	12	12 M / 0 F,<br>29 y	NW,<br>NR	300 ml water with lemon juice added with sucralose (0 kJ, 0 kcal) or water	80	1120 vs 1320	-100	
Appleton & Blundell[3]	10	0 M /10 F<br>X	NW / OW<br>NR	2 x 330 ml beverage with artifical sweeteners (42 kJ, 10 kcal) or water (0 kJ, 0 kcal) in the morning before lunch	90 + 180	616 vs 577	49	low LES consumers<br><br>test meal served 180 min after 1st drink and 90 min after 2nd drink (morning)
Appleton & Blundell[3]	10	0 M /10 F<br>X	NW / OW<br>NR	2 x 330 ml beverage with artifical sweeteners (42 kJ, 10 kcal) or water (0 kJ, 0 kcal) in the morning before lunch	90 + 180	917 vs 913	14	high LES consumers<br><br>test meal served 180 min after 1st drink and 90 min after 2nd drink (morning)

Adults<br><br>Reference	Sample size	Gender, age (y)<br><br>(mean, SD/SEM<br><br>or range)	Weight status, restraint status	LES and comparison preloads	Preload to test meal interval (min)	Intake test meal (kcal), LES vs comparison	∆ intake preload + test meal (kcal),<br><br>LES minus comparison (kcal)	Notes
Appleton & Blundell[3]	10	0 M /10 F<br>X	NW / OW<br>NR	2 x 330 ml beverage with artifical sweeteners (42 kJ, 10 kcal) or water (0 kJ, 0 kcal) in the morning before evening meal	90 + 180	605 vs 587	28	low LES consumers<br><br>test meal served 180 min after 1st drink and 90 min after 2nd drink (afternoon)
Appleton & Blundell[3]	10	0 M /10 F<br>X	NW / OW<br>NR	2 x 330 ml beverage with artifical sweeteners (42 kJ, 10 kcal) or water (0 kJ, 0 kcal) in the morning before evening meal	90 + 180	653 vs 681	-18	high LES consumers<br><br>test meal served 180 min after 1st drink and 90 min after 2nd drink (afternoon)
Akhavan et al.[130]<br>(Exp. 1)	14	14 M / 0 F, 19-28 y	NW,<br>NR	300 ml of orange-flavored water with 0.13 g sucralose (0 kJ, 0 kcal) or orange-flavored water	60	1319 vs 1418	-99	
Ford et al.[139]	8	7 M / 1 F<br>X	NW<br>X	50 ml of water with 42 mg sucralose (0 kJ,0 kcal) or 50 ml water	60	620 vs 562	58	

Adults<br><br>Reference	Sample size	Gender, age (y)<br><br>(mean, SD/SEM<br><br>or range)	Weight status, restraint status	LES and comparison preloads	Preload to test meal interval (min)	Intake test meal (kcal), LES vs comparison	Δ intake preload + test meal (kcal),<br><br>LES minus comparison (kcal)	Notes
Rogers et al.(131)	14	14 M / 0 F<br>25 y	NW<br>NR	300 ml of blackcurrant juice with sucralose (8 kJ, 2 kcal) or water	20	1150 vs 1134	18	
Rogers et al.(131)	18	0 M / 18 F<br>25 y	NW<br>NR	300 ml of blackcurrant juice with sucralose (8 kJ, 2 kcal) or water	20	808 vs 783	27	
Maersk et al.(132)	14	7 M / 7 F<br>34 (9.2)	OB<br>X	500 ml of diet cola with aspartame (7,5 kJ, 2 kcal) or water	240	1196 vs 1147	51	
Carvalho et al.(133)	24	13 m / 11 F<br>22 (2.33) y	NW<br>NR	500 ml of pineapple soda with sucralose (92 kJ, 22 kcal) or 500 ml water	150	1385 vs 1377	30	
Carvalho et al.(133)	27	13 M / 14 F<br>22 (1.84) y	NW / OW<br>NR	500 ml of sweetened beverage with sucralose (105 kJ, 25 kcal) or 500 ml water	150	1485 vs 1335	175	
Carvalho et al.(133)	27	13 M / 14 F<br>22 (1.84) y	NW / OW<br>NR	500 ml of sweetened beverage with sucralose (105 kJ, 25 kcal) or 500 ml water	150	1265 vs 1335	-45	

Adults <br>**Reference**	**Sample size**	**Gender, age (y)** <br>**(mean, SD/SEM** <br>**or range)**	**Weight status, restraint status**	**LES and comparison preloads**	**Preload to test meal interval (min)**	**Intake test meal (kcal), LES vs comparison**	**Δ intake preload + test meal (kcal),** <br>**LES minus comparison (kcal)**	**Notes**
Panahi et al.[134]	29	15 M / 14 F 22 (0.4) y	NW NR	373 g diet cola (0 kJ, 0 kcal) or 456 g water	0	926 vs 962	-36	Beverages consumed *ad libitum* <br>difference in intake diet cola and water NS

EI, energy intake; LES, low energy sweetener; Δ, change (difference from baseline to last time point unless otherwise described).

Table S10. Characteristics and results of short-term intervention studies comparing the effects on EI of LES versus nothing

LES vs nothing **Reference**	**Sample size**	**Gender, age (y) (mean, SD/SEM or range)**	**Weight status, restraint status**	**LES and comparison preloads**	**Preload to test meal interval (min)**	**Intake test meal (kcal), LES vs comparison**	**Δ cumulative intake (kcal), LES minus comparison (kcal)**	**Notes**
Rolls et al.[(113)]	13	13 M / 0 F 25 (4) y	NW Non-dieting	237 ml drink with 0.11 g aspartame (21 kJ, 5 kcal) or nothing	0	1022 vvs 1083	-56	
Rolls et al.[(113)]	13	13 M / 0 F 25 (4) y	NW Non-dieting	473 ml drink 0.22 gaspartame (42 kJ, 10 kcal) or nothing	0	1113 vs 1083	35	
Rolls et al.[(113)]	13	13 M / 0 F 25 (4) y	NW Non-dieting	237 ml drink with 0.11 g aspartame (21 kJ, 5 kcal) or nothing	30	1083 vs 1053	45	
Rolls et al.[(113)]	13	13 M / 0 F 25 (4) y	NW Non-dieting	473 ml drink 0.22 gaspartame (42 kJ, 10 kcal) or nothing	30	1138 vs 1053	90	
Rolls et al.[(113)]	13	13 M / 0 F 25 (4) y	NW Non-dieting	237 ml drink with 0.11 g aspartame (21 kJ, 5 kcal) or nothing	60	1211 vs 1150	66	
Rolls et al.[(113)]	13	13 M / 0 F 25 (4) y	NW Non-dieting	473 ml drink 0.22 gaspartame (42 kJ, 10 kcal) or nothing	60	1140 vs 1150	-5	
Monsivais et al.[(126)]	37	19 M / 18 F M: 23 (4) y F: 23 (3) y	M: NW / OW F: NW NR	475 ml of xx with aspartame (8 kJ, 2 kcal) or nothing	120	1009 vs 1008	3	

EI, energy intake; LES, low energy sweetener; Δ, change (difference from baseline to last time point unless otherwise described).

Table S11. Characteristics and results of short-term intervention studies comparing the effects on EI of LES in capsules versus placebo capsules

LES capsules **Reference**	**Sample size**	**Gender, age (y)** **(mean, SD/SEM** **or range)**	**Weight status, restraint status**	**LES and comparison preloads**	**Preload to test meal interval (min)**	**Intake test meal (kcal), LES vs comparison**	**Δ cumulative intake (kcal),** **LES minus comparison (kcal)**	**Notes**
Rogers et al.[136] (Exp. 1)	12	6 M / 6 F 18-26 y	NW NR	200 ml of water with 235 mg aspartame (21 kJ, 5 kcal) or water with 1.2 g corn flour (21 kJ, 5 kcal) in capsules	60	1088 vs 1263	-175	
Rogers et al.[136] (Exp. 2)	15	5 M / 10 F 19-24 y	NW NR	200 ml of water with 235 mg of aspartame (4 kJ, 1 kcal) or water with 235 mg corn flour (4 kJ, 1 kcal) in capsules	60	1329 vs 1467	-138	
Rogers et al.[136] (Exp. 2)	15	5 M / 10 F 19-24 y	NW NR	200 ml of water with 470mg of aspartame (8 kJ, 2 kcal) or water with 235 mg corn flour (4 kJ, 1 kcal) in capsules	60	1317 vs 1467	-149	
Rogers et al.[136]	16	6 M / 10 F 20-37 y	N=15, 22.4, N=1, 52.2 NR	200 ml water with 400 mg aspartame (8 kJ, 2 kcal) or 400 mg of cornflour (8 kJ, 2 kcal) in capsules		533 vs 485	-253	
Black et al.[138]	18	18 M / 0 F 19 y	NW X	280 ml of water with 340 mg aspartame in capsules (0 kcal) or water	60	1885 vs 1721	165	
Rogers & Blundell[140]	26	13 M / 0 F X	NW X	Aspartame (84 kJ, 20 kcal) or with 5.04 g alanine (84 kJ, 20	60 (aspartam e)/ 105	1103 vs 1330	-227	Between subjects,

LES capsules <br>Reference	Sample size	Gender, age (y) <br>(mean, SD/SEM <br>or range)	Weight status, restraint status	LES and comparison preloads	Preload to test meal interval (min)	Intake test meal (kcal), LES vs comparison	Δ cumulative intake (kcal), <br>LES minus comparison (kcal)	Notes
				kcal) in capsules	(alanine)			n=13/group
Rogers & Blundell[140]	13	13 M / 0 F X	NW X	Aspartame 169 kJ, 40 kcal) or with 10.08 g alanine (169 kJ, 40 kcal) in capsules	105	1124 vs 1232	-108	Within subjects
Rogers et al[125]	12	6 M / 6 F 18-30 y	NW Non-dieting	50 ml water with 400 mg aspartame (8 kJ, 2 kcal) or 400 mg of cornflour (8 kJ, 2 kcal) in capsules	5	785 vs 792	-7	
Rogers et al.[141]	12	6 M / 6 F 18-30 y	NW Non-dieting	50 ml water with 400 mg aspartame (8 kJ, 2 kcal) or 400 mg of cornflour (8 kJ, 2 kcal) in capsules	30	856 vs 814	42	
Rogers et al.[141]	12	6 M / 6 F 18-30 y	NW Non-dieting	50 ml water with 400 mg aspartame (8 kJ, 2 kcal) or 400 mg of cornflour (8 kJ, 2 kcal) in capsules	60	705 vs 875	-170	
Rogers et al.[142]	17	10 M / 7 F 18-29 y	Normal weight for height non-dieting, NR	450 mg AceK (0 kcal) vs 500 mg cornflour (8 kJ, 2 kcal) in a capsule	60	1033 vs 1045	-14	

LES capsules <br>**Reference**	**Sample size**	**Gender, age (y)** <br>**(mean, SD/SEM** <br>**or range)**	**Weight status, restraint status**	**LES and comparison preloads**	**Preload to test meal interval (min)**	**Intake test meal (kcal), LES vs comparison**	**Δ cumulative intake (kcal),** <br>**LES minus comparison (kcal)**	**Notes**
Rogers et al.[(142)]	17	10 M / 7 F 18-29 y	Normal weight for height non-dieting, NR	500 mg aspartame (8 kJ, 2 kcal) vs 500 mg cornflour (8 kJ, 2 kcal) in a capsule	60	948 vs 1045	-97	
Rogers et al.[(142)]	17	10 M / 7 F 18-29 y	Normal weight for height non-dieting, NR	330 mg saccharin (0 kcal) vs 500 mg cornflour (8 kJ, 2 kcal) in a capsule	60	1028 vs 1045	-19	
Rogers et al.[(142)]	17	10 M / 7 F 18-29 y	Normal weight for height non-dieting, NR	150 mg sucralose (0 kcal) vs 500 mg cornflour (8 kJ, 2 kcal) in a capsule	60	1021 vs 1045	-26	

EI, energy intake; LES, low energy sweetener; Δ, change (difference from baseline to last time point unless otherwise described)

Tables S12. Results of meta-regression analyses for the short-term intervention studies

LES versus sugar

Factor	B	P	95% CI
Gender (ref = Female)			
Male	14.8	0.644	-49.0 to 78.6
Mixed	-10.3	0.754	-75.3 to 54.8
Comparison preload (kcal)	-0.46	<0.001	-0.63 to -0.30
Preload to test meal time interval (ref = 0 mins)			
0 to 30 mins	87.4	0.032	7.9 to 166.9
>30 to 60 mins	66.4	0.086	-9.7 to 142.4
>60 mins	8.2	0.820	-63.7 to 80.1
Year	-2.8	0.045	-5.64 to -0.06

Residual I^2 = 69.2%
Adjusted R^2 = 47.8%

LES versus sugar (COMPX)

Factor	B	P	95% CI
Gender (ref = Female)			
Male	3.07	0.863	-32.4 to 38.6
Mixed	-16.7	0.366	-53.4 to 20.0
Comparison preload (kcal)	-0.04	0.348	-0.12 to 0.04
Preload to test meal time interval (ref = 0 mins)			
0 to 30 mins	49.2	0.036	3.3 to 95.1
>30 to 60 mins	44.4	0.053	-0.68 to 89.4
>60 mins	9.2	0.663	-32.9 to 51.4
Year	-1.31	0.098	-2.86 to 0.25

Residual I^2 = 64.9%
Adjusted R^2 = 12.0%

LES versus unsweetened

Factor	B	P	95% CI
Gender (ref = Female)			
Male	-99.0	0.233	-281.8 to 83.8
Mixed	151.3	0.030	20.7 to 281.8
Comparison preload (kcal)	0.66	0.042	0.03 to 1.30
Preload to test meal time interval (ref = 0 mins)			
0 to 30 mins	216.3	0.071	-24.7 to 457.3
>30 to 60 mins	44.3	0.554	-128.6 to 217.1
>60 mins	27.4	0.719	-149.9 to 204.6

Residual I^2 = 61.2%
Adjusted R^2 = 58.6%

LES versus water

Factor	B	P	95% CI
Gender (ref = Female)			
Male	51.3	0.400	-72.1 to 174.8
Mixed	-34.3	0.431	-122.3 to 53.7
Comparison preload (kcal)	-3.4	0.827	-35.0 to 28.2
Preload to test meal time interval (ref = 0 mins)			
0 to 30 mins	42.6	0.499	-85.0 to 170.3
>30 to 60 mins	4.9	0.936	-119.2 to 128.9
>60 mins	57.6	0.421	-87.2 to 202.4

Residual I^2 = 0.0%
Adjusted R^2 = 20.5%

LES versus nothing

Meta-regression not undertaken due to the small number of studies.

LES in capsules versus placebo capsules

Factor	B	P	95% CI
Gender (ref = Female)			
Male	N/A	N/A	N/A
Mixed	-307.6	0.017	-524.6 to -90.7
Comparison preload (kcal)	-6.9	0.082	-15.1 to 1.4
Preload to test meal time interval (ref = 0 mins)			
0 to 30 mins	N/A	N/A	N/A
>30 mins	-146.9	0.012	-239.2 to -54.6
Year	6.9	0.062	-0.56 to 14.3

Residual I^2 = 0.0%
Adjusted R^2 = 100.0%

Sensitivity analyses for short-term intervention studies

Sensitivity analyses were conducted to determine the effect of replacing missing SDs with those from other studies (as detailed in the Supplementary Information (SI)). The results are shown in the Table below. They indicate that the summary effect estimates were not affected by using less conservative replacement SD values. Also, the summary effect estimates were not affected by excluding studies which did not report SDs. The inclusion of results from repeated measures on the same participants in the estimation of summary effects (as detailed in the SI) attenuated the summary effect estimates slightly towards the null, but did not affect the overall findings of the meta-analyses.

Table S13. Summary of sensitivity analyses for short-term intervention studies

Main results: Imputation of missing SDs with maximum SDs

Difference in total energy (overall)	Sensitivity analyses			
	Main results	SA1	SA2	SA3
**LES vs sugar**				
Summary ES	-94 kcal	-94 kcal	-95 kcal	-102 kcal
95% CI	-122 to -66 kcal	-121 to -67 kcal	-124 to -67 kcal	-134 to -70 kcal
Number of comparisons	68	68	62	118
**LES vs unsweetened**				
Summary ES	21 kcal	22 kcal	18 kcal	12 kcal
95% CI	-41 to 83 kcal	-30 to 73 kcal	-56 to 93 kcal	-29 to 52 kcal
Number of comparisons	13	13	10	21
**LES vs water**				
Summary ES	-2 kcal	-5 kcal	2 kcal	-6 kcal
95% CI	-30 to 26 kcal	-32 to 22 kcal	-29 to 32 kcal	-38 to 26 kcal
Number of comparisons	35	35	30	57
**LES vs nothing**		N/A*	N/A*	
Summary ES	18 kcal			22 kcal
95% CI	-32 to 69 kcal			-32 to 77 kcal
Number of comparisons	4			7
**LES in capsules**		N/A*	N/A*	
Summary ES	-69 kcal			-77 kcal
95% CI	-140 to 3 kcal			-176 to 23 kcal
Number of comparisons	9			13
**Difference in COMPX (overall)**				
**LES vs sugar**				
Summary ES	50.5%	51.1%	50.4%	51.7%
95% CI	39.0% to 62.0%	39.9% to 62.4%	38.7% to 62.2%	37.9% to 65.5%
Number of comparisons	68	68	62	118

SA1: Imputation of missing SDs with mean SDs
SA2: Exclusion of studies with no reported SDs
SA3: Use of robust variance estimation to include repeated measures (where appropriate)
*All studies reported SDs, therefore these sensitivity analyses were not undertaken

Table S14. Characteristics and results of sustained intervention studies comparing the effects on EI and/or anthropometric measures of LES versus sugar and LES versus water

Reference	Design,<br><br>Location, n	Subjects:<br><br>M/F,mean age and BMI	Intervention<br><br>(nutritional characteristics)	Duration and n at completion or follow-up	Blinding	Energy intake (reported values)	Anthropometric measures (reported values)
*Kanders et al.[8]	Parallel design n=59:<br>LES (n=29) vs control with advice to avoid LES (n=30)<br><br>USA	13 M / 46 F,<br>46 y, BMI 37	LES (aspartame) sweetened dairy products in place of unsweetened; encouraged to use LES table-top, soft drinks, desserts<br><br>Control: advised to avoid LES.	12 wk weight loss (3-wk run-in before randomization to treatment)<br><br>Completers n=55:<br><br>LES 28, Control 27	Not blinded	Not reported	**ΔBW wk 0-12**<br>LES: -7.9 kg<br>control - 7.6 kg<br><br>**Δ %BW wk 0-12**<br>LES: -7.8%<br>Control: -6.9%<br><br>**ΔBMI units wk 0-12**<br>LES: -2.9<br>Control: -2.6<br><br>Weighted mean combined both genders (data from n=54)
Tordoff & Alleva[17]	Cross-over design<br>LES vs. sucrose vs. no beverage<br>n=41 randomized<br><br>USA	21 M/ 9 F,<br>25 y, BMI 25.2<br><br>(Completers only)	4*300 ml soda/day:<br>LES: 3 kcal/day<br>SSB (sugar-control): 530 kcal/d<br>Control (no drink): No soda	3 wk per treatment counterbalanced<br>(9 wks in total)<br><br>Completers n=30	Covert for LES vs SSB	**EI**<br>LES: 2647 kcal/d<br>SSB: 3175 kcal/d<br>No-drink control: 2801 kcal/d<br><br>Weighted mean combined both genders	**ΔBW**<br>LES: -0.28 kg<br>SSB: +0.63 kg<br>No-drink control: +0.19<br><br>Weighted mean combined both genders

Reference	Design,<br><br>Location, n	Subjects:<br><br>M/F,mean age and BMI	Intervention<br><br>(nutritional characteristics)	Duration and n at completion or follow-up	Blinding	Energy intake (reported values)	Anthropometric measures (reported values)
Wolraich et al.[18]	Cross-over design aspartame vs. saccharin vs. sucrose with pre-school (3-5 yr) and primary school children (6-10 yr)<br>n=58 randomized	M / F not reported, preschool 4.7 yr, primary school 8.1 yr,<br>weight status not reported	Manipulated foods (a.o. fruit juice, cereals, pudding, yogurt, cookies, soft drinks) with<br>Aspartame (Asp)<br>Saccharin (Sacch)<br>Sucrose (Sugar)	3 wk per treatment counterbalanced<br>(9 wks in total)<br><br>Completers n=48:<br>pre-school 25, primary school 23	Covert, subjects blinded to treatments	**Mean EI**<br>Preschool children:<br>LES (Asp): 1604 kcal/d<br>LES (Sacch): 1587 kcal/d<br>Sucrose: 1847 kcal/d<br><br>Primary school children:<br>LES (Asp): 1936 kcal/d<br>LES (Sacch): 1980 kcal/d<br>Sucrose: 2221 kcal/d	**Not reported**
Naismith & Rhodes[10]<br><br>Experiment 2	Parallel design LES vs sugar<br>n=8	8 M / 0 F,<br>24 yr, BMI 21	Experiment 2 only ((n=8):<br>LES: LES (aspartame, AceK) replaced sugar (equal to -451 kcal/d)<br>Control: Sugar used or supplemented to wide range of foods/beverages	10 d/treatment<br>3 d run-in<br>(= 23 d total)<br><br>Completers n=8	Covert, subjects blinded to treatments (foods provided in metabolic ward)	**ΔEI**<br><br>LES vs sugar: -297 kcal	%ΔBW vs baseline Overall mean LES vs sugar:<br><br>-0.051 kg

Reference	Design,<br><br>Location, n	Subjects:<br><br>M/F,mean age and BMI	Intervention<br><br>(nutritional characteristics)	Duration and n at completion or follow-up	Blinding	Energy intake (reported values)	Anthropometric measures (reported values)
*Blackburn et al.[6]	Parallel design n=163:<br>LES (n= 82) vs control with caloric sweeteners (n=81).<br><br>USA	0 M / 163 F,<br>42 y, BMI 37	LES: Aspartame products during active weight loss, encouraged to use LES during follow-up<br><br>Control: Use of LES products or substitution discouraged	3 wk run-in, 16 wk weight loss (wks 4-19), follow-up at weeks 71 and 175.<br><br>Wk 19: n=139 (LES 71, Control 65)<br><br>Wk 71: n=125 (LES 61, Control 64)<br><br>Wk 175: n=83 (LES 42, Control 41)	Blinding of subjects not possible	**ΔEI wk 0-71:**<br>LES: -2.45 MJ/d<br><br>(-585 kcal/d)<br><br>Control: -1.90 MJ/d<br><br>(-454 kcal/d)<br><br>**ΔEI wk 19-71:**<br>LES: +0.73 MJ/d(+174 kcald/d)<br><br>Control: +0.95 MJ/d(+227 kcal/d)<br><br>Wk 175: EI not reported	**ΔBW wk 0-175:**<br>LES: -5.1 kg<br>Control: 0 kg (reported as 'no net change')<br><br>**ΔBW wk 19-175:**<br>LES: +5.4 kg<br>Control: +9.4 kg<br><br>Intention-to-treat analysis per study phase (weight loss or maintenance)

Reference	Design,<br><br>Location, n	Subjects:<br><br>M/F,mean age and BMI	Intervention<br><br>(nutritional characteristics)	Duration and n at completion or follow-up	Blinding	Energy intake (reported values)	Anthropometric measures (reported values)
*Raben et al.[13]	Parallel design LES vs sucrose n=41<br><br>Denmark	6 M / 35 F, 35 y, BMI 27.8	LES: food and drinks with LES<br><br>Control: similar food and drinks with sucrose<br><br>Target: to replace 2g/kg/d sucrose with LES (54% aspartame, 22% AceK, 23% cyclamate, 1% saccharin)<br><br>70% substituted via drinks, 30% via foods	10 wk<br><br>Completers n:<br><br>LES 20, Sucrose 21	Covert, Subjects blinded to treatments	**ΔEI**<br>LES: -0.44 MJ/d<br><br>(-105 kcal/d)<br>Sucrose: +1.71 MJ/d<br><br>(+408 kcal/d)	**ΔBW**<br>LES: -1.0 kg<br>Sucrose: +1.6 kg<br><br>**Δfat mass**<br>LES: -0.7 kg<br>Sucrose: +1.3 kg<br><br>**Δlean mass**<br>LES: -0.3 kg<br>Sucrose: +0.3 kg
*Reid et al.[14]	Parallel design LES vs SSB n=161 randomized<br><br>UK	0 M / 133 F, 32 y, BMI 22.5<br><br>(Completers)	4 x 250 ml drinks/d sweetened with aspartame or sucrose<br><br>LES: 0.07 MJ/d<br>Control: 1.8 MJ/d	4 wk,<br>1 wk baseline<br>(5 wk in total) ,<br><br>Completers n=133<br><br>(Correctly informed: LES 33, sucrose 33; Misinformed LES 32, sucrose 35)	Subjects 'correctly informed' or 'mis-informed'	**ΔEI**<br>LES: -0.39 MJ/d<br><br>(-93 kcal/d)<br>SSB: +0.79 MJ/d<br>(+189 kcal/d)	**ΔBW**<br>LES: -0.37 kg<br>SSB: +0.08 kg<br><br>Data from correctly informed subjects only, provided by authors

Reference	Design,<br><br>Location, n	Subjects:<br><br>M/F,mean age and BMI	Intervention<br><br>(nutritional characteristics)	Duration and n at completion or follow-up	Blinding	Energy intake (reported values)	Anthropometric measures (reported values)
*Njike et al.[11]	Cross-over design<br><br>LES cocoa vs SSB cocoa comparison n=37<br><br>USA	6 M / 31 F,<br>52 y, BMI 30.3	2/d cocoa beverage with LES (90 kcal/d) or sugar control (460 kcal/d)	6 wk/treatment,<br>4 wk washouts<br>(26 wks in total)<br><br>Completers n:<br><br>LES 32, Control 33	Double-blind, covert	**Mean EI**<br>LES: 1779 kcal/d<br>SSB: 1991 kcal/d	**ΔBW**<br>LES: 0.0 kg<br>SSB: +0.2 kg<br><br>**ΔBMI**<br>LES: 0.0<br>SSB: +0.1<br><br>**Δ waist circumf.**<br>LES: -1.8 cm<br>SSB: -1.3 cm
*Reid et al.[122]	Parallel design LES vs SSB<br><br>n=71 randomized<br><br>UK	0 M / 71 F,<br>34 y, BMI 27.5 with history of dieting	4*250 ml drinks sweetened with aspartame (0.07 MJ/d) or sucrose (1.8 MJ/d)	4 wk,<br>1 wk baseline<br>(5 wk in total)<br><br>Completers n=53:<br><br>LES 29, sucrose 24	Subjects 'correctly informed' or 'mis-informed'	**ΔEI**<br>LES: -0.67 MJ/d<br><br>(-160 kcal/d)<br>SSB: -0.14 MJ/d<br>(-33 kcal/d)	**ΔBW**<br><br>LES: 0.08 kg<br>SSB: +0.57 kg<br><br>Data provided by authors
*Maersk et al.[9]<br><br>LES v SSB comparison	Parallel design LES vs SSB n=22<br><br>Denmark	9 M / 13 F<br><br>39 y, BMI 32.1	LES: 1 L/d of LES cola (45 kcal/d)<br><br>Control: 1 L/d of SSB cola (430 kcal/day)	6 months<br><br>Completers n=22:<br><br>LES 12, SSB 10	Covert, subjects ‘blind’ to treatments	Not reported	**%ΔBW vs baseline**<br>LES: +0.114%<br>SSB: +1.28%<br><br>**Δfat mass vs baseline**<br>LES: -0.052 kg<br>SSB: +3.14 kg

Reference	Design,<br><br>Location, n	Subjects:<br><br>M/F,mean age and BMI	Intervention<br><br>(nutritional characteristics)	Duration and n at completion or follow-up	Blinding	Energy intake (reported values)	Anthropometric measures (reported values)
*Maersk et al.[9]<br><br>LES v Water comparison	Parallel design LES vs water n=25<br><br>Denmark	8 M / 17 F<br><br>39 y, BMI 32.6	LES: 1 L/d of LES cola (45 kcal/d)<br><br>Control: 1 L/d of water (0 kcal/d)	Completers n=22:<br><br>LES 12, water 13	Subjects 'blind' to treatments	Not reported	**%ΔBW vs baseline**<br>LES: +0.114%<br><br>Water: +0.576%<br><br>**Δfat mass vs baseline**<br>LES: -0.052<br><br>Water: +0.49 kg
*De Ruyter et al.[7]	Parallel design n=641:<br>LES (n=319) vs SSB (n=322)<br><br>Netherlands	340 M / 301 F, 8 y, BMI z-score 0.03 (SD units vs. national mean)	Once 250 ml/d drinks with LES (34 mg sucralose/12 mg AceK) vs. control with sucrose.<br><br>LES: 0 kcal/d<br>SSB: 104 kcal/d	18 months<br><br>Completers n=477:<br><br>LES 225, SSB 252	Double-blind	Not reported	**ΔBMI z-score**<br>LES: +0.02<br>SSB: +0.15<br><br>**ΔBW**<br>LES: +6.35 kg,<br>SSBl: +7.37 kg<br><br>Data for completers and imputed for non-completers

Reference	Design, Location, n	Subjects: M/F,mean age and BMI	Intervention (nutritional characteristics)	Duration and n at completion or follow-up	Blinding	Energy intake (reported values)	Anthropometric measures (reported values)
*Tate et al.[16]<br>CHOICE trial<br>LES v SSB comparison	Parallel design n=210: Substituting SSB with LES (n=105) vs control (n=105, no specific substitution)<br>USA	38 M/ 172 F, 41 yr, BMI 36.2, consuming ≥280 kcal/d from beverages	Substitute ≥2 servings/d (≥200 kcal/d) of SSB with LES vs 'dietary advice' with no specific beverage advice or intervention in a weight control program	6 months<br>Completers n=181:<br>LES 93, SSB 88	Blinding of subjects not possible	**ΔEI**<br>LES: -658 kcal/d<br>SSB: -581 kcal/d	**ΔBW**<br>LES: -2.6 kg<br>SSB: -1.9 kg<br>**Δwaist circumference**<br>LES: -2.1 cm<br>SSB: -2.1 cm<br>Intention-to-treat analysis
*Tate et al.[16]<br>CHOICE trial<br>LES v Water comparison	Parallel design n=213:<br>substituting SSB with LES (n=105) vs water (n=108)<br>USA	35 M/ 178 F, 42 yr, BMI 35.8, consuming ≥280 kcal/d from beverages	Substitute ≥2 servings/d (≥200 kcal/d) of caloric beverages with LES vs water in a weight control program	6 months<br>Completers n=184:<br>LES 93, water 91	Blinding of subjects not possible	**ΔEI**<br>LES: -658 kcal/d<br>Water: -532 kcal/d	**ΔBW**<br>LES: -2.6 kg<br>Water: -1.9 kg<br>**Δwaist circumference**<br>LES: -2.1 cm<br>Water: -2.0 cm<br>Intent to treat analysis

Reference	Design, Location, n	Subjects: M/F,mean age and BMI	Intervention (nutritional characteristics)	Duration and n at completion or follow-up	Blinding	Energy intake (reported values)	Anthropometric measures (reported values)
*Peters et al.[12]	Parallel design n=303: LES (n=154) vs water (n=149) USA	255F/48M, 48 yr, BMI 33.7, consuming at least 3 LES beverages per week	Intervention carried out within a behavioral weight loss treatment program<br>LES: Counselled to consume ≥24 US ounces (710 ml) LES beverages per day<br>Water: Same but water (and avoid LES beverages)	12 weeks<br>Completers n=279:<br>LES 145, water 134	Blinding of subjects not possible	Not reported	**ΔBW**<br>LES: -5.95 kg,<br>Water: -4.09 kg<br>**Δwaist circumference**<br>LES: -5.73 cm<br>Water: -4.36 cm<br>Intention-to-treat analysis

* - included in the meta-analysis
SSB = sugars-sweetened beverages (includes pure fruit juice)
LES = low energy sweetener
Δ = Change (difference from baseline to last time point unless otherwise described)
EI = Energy intake
BW = Body Weight, kg

Table S15. Results of meta-regression analyses for the sustained intervention studies

Factor	B	P	95% CI
Length of follow-up (months)	-0.09	0.179	-0.25 to 0.07
Gender (ref = Female)			
Male	N/A	N/A	N/A
Mixed	1.08	0.484	-3.25 to 5.42
Weight category (ref = Normal weight)			
Overweight	-1.36	0.376	-5.52 to 2.81
Obese	-1.01	0.538	-5.67 to 3.64

Residual I^2 = 87.0%
Adjusted R^2 = 40.7%

Table S16. Summary of sensitivity analyses for sustained intervention studies

		Sensitivity analyses	
	Main results	SA1	SA2
**LES vs sugar**			
Summary ES	-1.35 kg	-1.32 kg	-1.43 kg
95% CI	-2.28 to -0.42 kg	-2.23 to -0.42 kg	-2.41 to -0.45 kg
Number of studies	9	9	8

Main results: Imputation of missing SDs with maximum SDs
SA1: Imputation of missing SDs with mean SDs
SA2: Exclusion of studies with no reported SDs

Sensitivity analyses were conducted to examine the influence of using imputed missing SDs on the overall effect estimates. The results are summarised in the Table below. They revealed only negligible differences between the magnitude of estimates from the main analyses and those from the sensitivity analyses, and no difference in direction of effect. This suggests that the imputed SDs did not substantially alter the results of the analysis.

Table S17. Summary of risk of bias assessments for the sustained intervention studies

	Sequence generation	Allocation concealment	Blinding of participants	Blinding of personnel	Blinding of outcome assessors	Completeness of outcome data	Selective outcome reporting
Kanders et al.(8)	?	?	-[a]	-[a]	?	+[b]	?
Tordoff & Alleva(17)	?	?	+	?	?	-[c]	+[d]
Wolraich et al.(18)	+	+[f]	+	+	+	-[f]	?[e]
Naismith & Rhodes(10)	?	?	+	-[a]	?	+	+[d]
Blackburn et al.(6)	+	?	-[a]	-[a]	?	+	+[d]
Raben et al.(143)	?	?	-[a]	-[a]	?	+	+[d,g]
Reid et al.(14)	?	?	-	?	?	-[c]	+[h]
Nijke et al.(11)	+	?	+	+	+	+	+
Reid et al.(15)	?	?	-	?[i]	?[i]	-[c]	+[h]
Maersk et al.(9)	?	?	-[a]	-	?	-[j]	+[f]
de Ruyter et al.(7)	+	+	+	+	+	+	+[f]
Tate et al.(16)	+	+	-[a]	?[i]	?[i]	+[k]	+[f]
Peters et al.(12)	+	?	-[a]	-	-[l]	+	+[f]

+ / - / ? = Assessed that criterion has been met (+) / not met (-) / could not be determined (?)
[a] Not possible due to nature of intervention
[b] No intention-to-treat (ITT) analysis but dropouts <10% of randomized population
[c] No ITT analysis but dropouts >10% of randomized population
[d] No registered protocol, but the list in methods includes the variables relevant to energy balance (potential to explain group differences), and all are reported in outcomes. It is conceivable other variables were measured, but these are unlikely to be a source of bias for EI or BW outcomes.
[e] It is conceivable that other variables (e.g. body weight) were measured and not reported
[f] Relevant outcomes for energy balance reported as per registered protocol
[g] Complete for relevant outcomes but DEXA data were not used and the measurement of energy metabolism in a subset of participants is not reported but described in a later paper
[h] Body weight numerical data not reported but accessed from authors
[i] Described as "single blind" but does not explicitly state who was blinded
[j] Attrition imbalanced between groups, missing values not imputed
[k] ITT on all analyses except blood pressure and glucose
[l] Group leaders recording outcomes were likely to be aware of assignment

Table S18. Summary of methodological quality assessment for sustained intervention studies

	Similar on baseline characteristics	Power calculation conducted	Power reached	Withdrawal numbers reported? By group?	Reasons for withdrawals reported? / By group?
Kanders et al.[(8)]	Y[a]	NA[b]	NA	Y/Y	Y/Y
Tordoff & Alleva[(17)]	NA	NR	NR	Y/NA	Y/NA
Wolraich et al.[(18)]	NA	NR	NR	Y/NA	Y/NA
Naismith & Rhodes[(10)]	NA	NR	NR	NA	NA
Blackburn et al.[(6)]	Y	Y	?	Y/Y	Y/N
Raben et al.[(13)]	Y	NR	NR	NR	NR
Reid et al.[(14)]	?[c]	Y	?	Y/N	Y/N
Nijke et al.[(11)]	Y	Y	?	Y/Y	Y/Y
Reid et al.[(15)]	Y	Y	N[d]	N/N	N/N
Maersk et al.[(9)]	N[e]	NR	NR	Y/Y	N/N
de Ruyter et al.[(7)]	Y[f]	Y	Y	Y/Y	Y/Y
Tate et al.[(16)]	Y	Y	NR	Y/Y	N/N
Peters et al.[(12)]	Y	Y	N[d,g]	Y/Y	N/N

Y = yes, N = No, NR= not reported, NA= not applicable, ? = unclear

[a]Control group had lower LES intake at baseline
[b]Feasibility trial, no power calculation applicable
[c]Not reported by treatment (sucrose and LES) group
[d]Fell below sample size calculation
[e]Unequal distribution of genders across treatments
[f]Slight difference in parental levels of education
[g]Powered for n=150 per group but fell to n=149 in water group

Comparison of the present review with Miller and Perez[144]

Miller and Perez[144] also reviewed prospective cohort and sustained intervention studies on LES and body weight. They did not review relevant evidence from animal studies, or from short-term intervention studies on effects of LES on food intake. With respect to the prospective cohort and sustained intervention studies there are differences in the data or studies included in our review and Miller and Perez's review as summarized below in **Tables S19 and S20**.

Table S19. Prospective cohort studies that differed in inclusion/exclusion between the present review and the review by Miller and Perez[144]

Study	Present review	Miller and Perez	Comments
Chen[26]	Included	Not included	
Colditz[145]	Excluded	Included	Excluded as Pan et al included a more recent and detailed analysis of this cohort
Duffey[27]	Included	Not included	
Johnson[146]	Excluded	Included	Excluded as less than 500 subjects
Ludwig[28]	Included	Not included	
Newby[147]	Excluded	Included	Excluded as follow up less than one year
Pan[22]	Included	Not included	
Parker[148]	Excluded	Included	Excluded as less than 500 subjects
Schulze[149]	Excluded	Included	Excluded as Pan et al included a more recent and detailed analysis of this cohort
Striegel- Moore[23]	Included	Not included	
Vaneslow[30]	Included	Not included	

Table S20. Sustained intervention studies that differed in inclusion/exclusion between the present review and the review by Miller and Perez[144].

In addition, Peters et al.[12] in the present review was published after the Miller and Perez[144] cut-off date.

Study	Present review	Miller and Perez	Comments
Knopp[150]	Excluded	Included in meta-analysis	Aspartame versus lactose capsules. While relevant to understanding mechanisms, we excluded on the grounds that this is not how LES are consumed in the diet.
Tordoff and Alleva[17]	Included in narrative section only	Included in meta-analysis	Below our cut-off duration of 4 weeks for inclusion in meta-analysis
Naismith and Rhodes[10]	Included in narrative section only	Excluded	Below Miller and Perez's cut-off duration of 2 weeks and our cut-off duration of 4 weeks for inclusion in meta-analysis
Blackburn et al.[6]	Week 175 results included in meta-analysis	Week 19 results included in meta-analysis	This study had an active weight loss period of 19 weeks with follow-ups at weeks 71 and 175. Participants were advised to continue their intervention throughout. We included the data for the last available intervention measurement as per our protocol.
Gatenby et al.[151]	Excluded	Included in meta-analysis	Participants were advised to reduce sugar intake in general, and not only through replacement with LES
Gostner et al.[152]	Excluded	Included in meta-analysis	Sweetener investigated was isomalt, which has an energy value of 2.1 kcal/g and approximately half that of sucrose, so not a LES by our definition
Ebbeling et al.[153,154]	Excluded	Included in meta-analysis	These were mixed interventions. Participants were offered LES or water (versus sugar-sweetened beverages), and these were not separated in the analyses

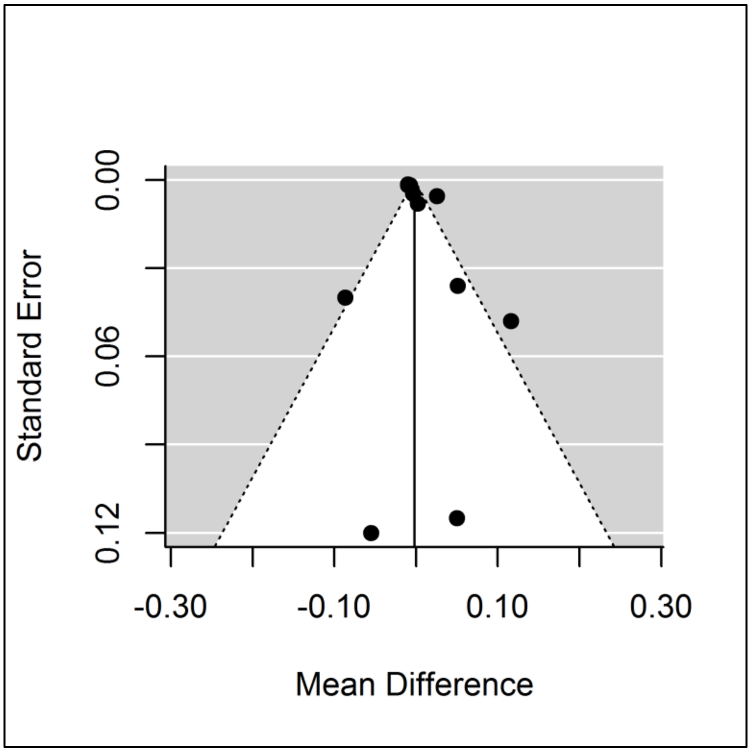

Figure S1. Funnel plot of prospective cohort studies reporting information on association between LES consumption and body weight status change.

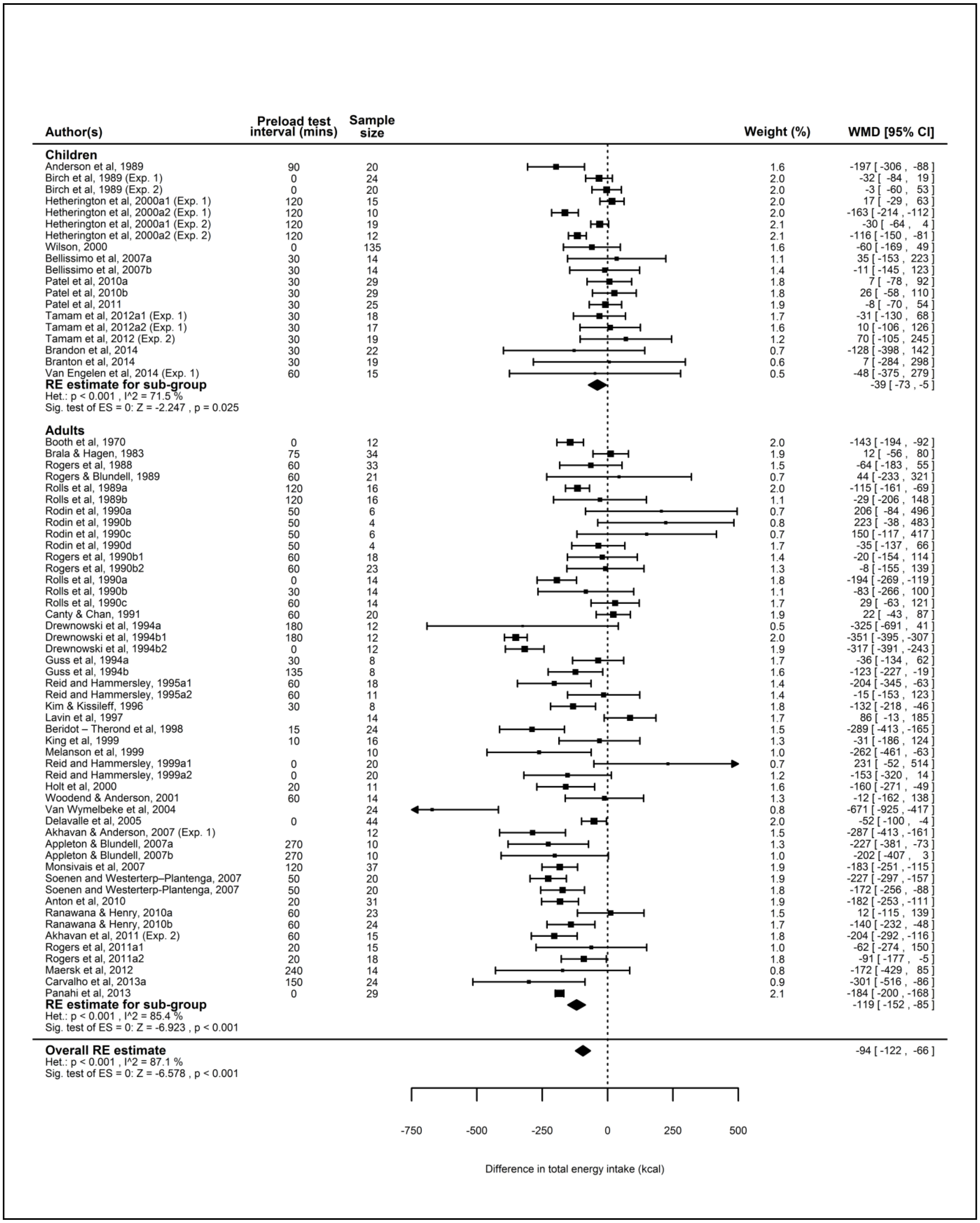

Figure S2. Forest plot of short-term intervention studies comparing the effects on EI of LES versus sugar in children and in adults.

Difference in total EI is the difference in cumulative EI (preload plus test meal) for the LES condition minus the sugar condition. Squares represent mean difference in EI for the individual comparisons; square size is proportional to the weight of each comparison; horizontal lines represent 95%CIs; diamonds represent the summary estimates and 95%CIs from random effects models for comparisons in children and adults separately and for all of the comparisons.

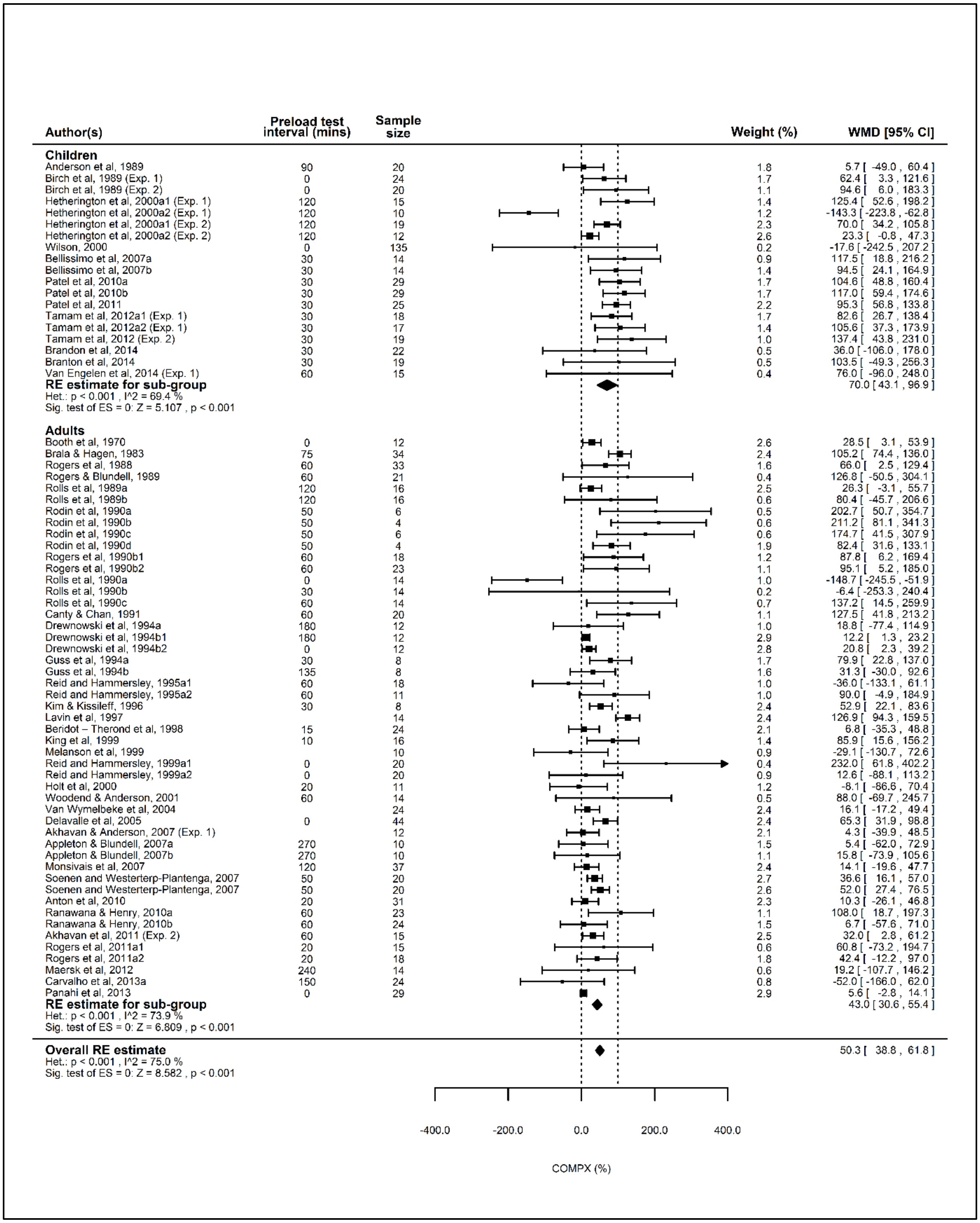

Figure S3. Forest plot of short-term intervention studies comparing the effects on COMPX of LES versus sugar in children and in adults.

COMPX is (EI in test meal after LES minus EI in test meal after sugar) / (EI from sugar preload minus EI from LES preload) expressed as percentage. Squares represent mean COMPX for the individual comparisons; square size is proportional to the weight of each comparison; horizontal lines represent 95%CIs; filled diamonds represent the summary estimates and 95%CIs from random effects models for comparisons in children and adults separately and for all of the comparisons. The two reference lines represent no compensation (0%, i.e., the amount eaten in the test meal is the same after the LES and the sugar preloads), and full compensation (100%, i.e., the amount eaten in the test meal is greater after the LES preload than after the sugar preload and that greater amount equals the energy difference between the sugar and LES preloads).

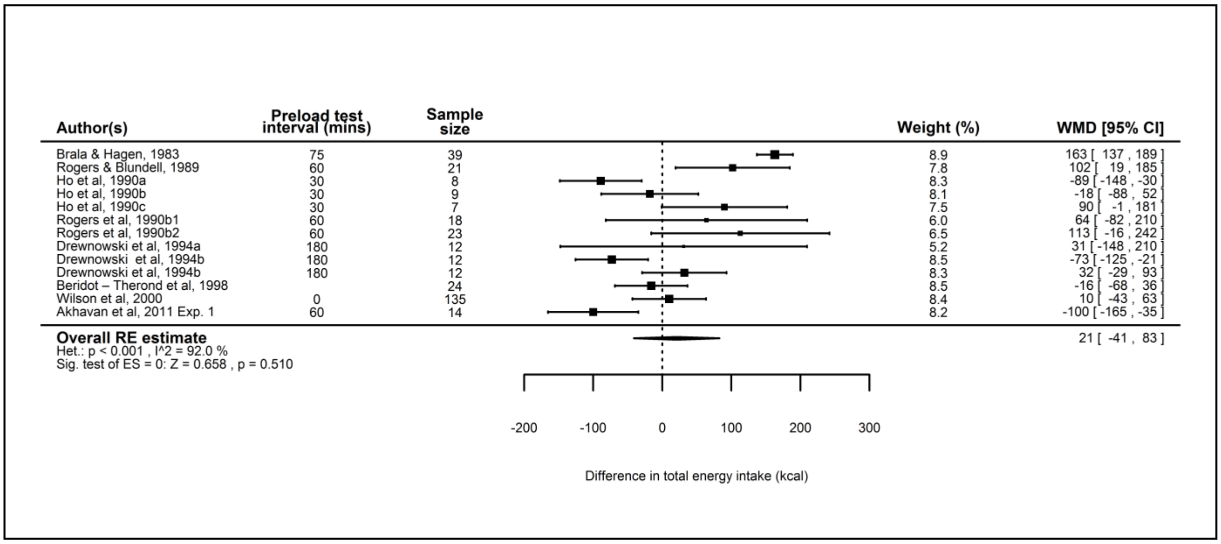

Figure S4. Forest plot of short-term intervention studies comparing the effects on EI of LES-sweetened versus unsweetened products.

Difference in total EI is the difference in cumulative EI (preload plus test meal) for the LES condition minus the unsweetened condition. Squares represent mean difference in EI for the individual comparisons; square size is proportional to the weight of each comparison; horizontal lines represent 95%CIs; the filled diamond represents the summary estimates and 95%CIs from random effects models for all of the comparisons.

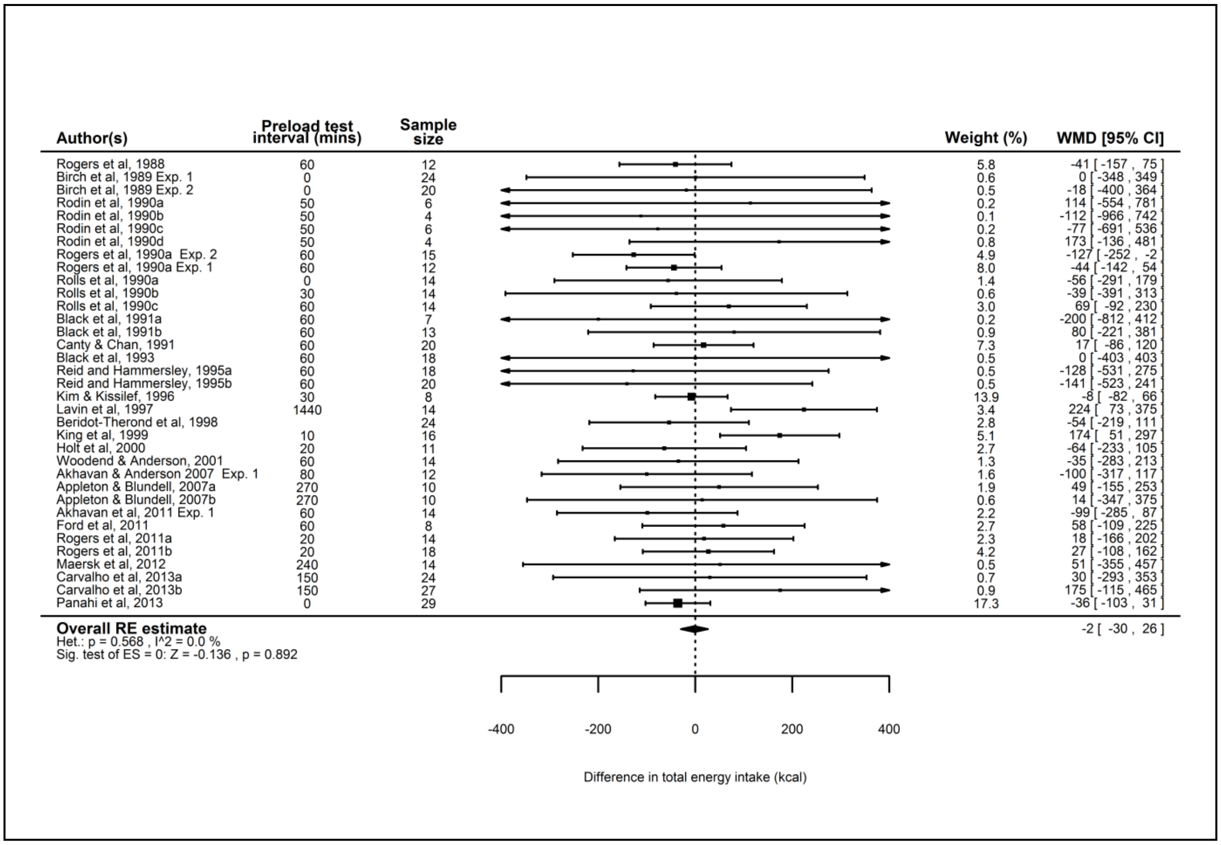

Figure S5. Forest plot of short-term intervention studies comparing the effects on EI of LES versus water.

Difference in total EI is the difference in cumulative EI (preload plus test meal) for the LES condition minus the water condition. Squares represent mean difference in EI for the individual comparisons; square size is proportional to the weight of each comparison; horizontal lines represent 95%CIs; the filled diamond represents the summary estimates and 95%CIs from random effects models for all of the comparisons.

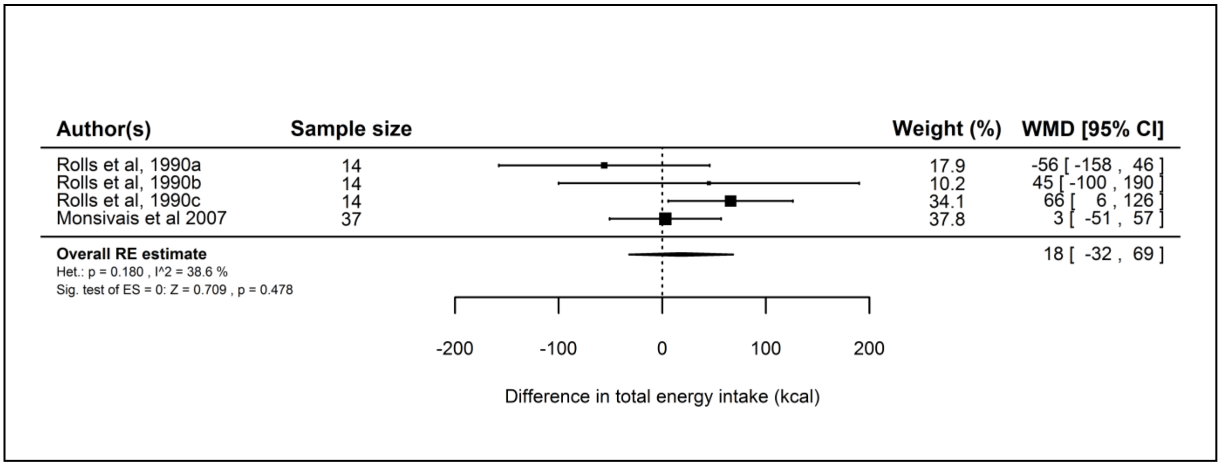

Figure S6. Forest plot of short-term intervention studies comparing the effects on EI of LES versus nothing.

Difference in total EI is the difference in cumulative EI (preload plus test meal) for the LES condition minus the nothing condition. Squares represent mean difference in EI for the individual comparisons; square size is proportional to the weight of each comparison; horizontal lines represent 95% CIs; the filled diamond represents the summary estimates and 95% CIs from random effects models for all of the comparisons.

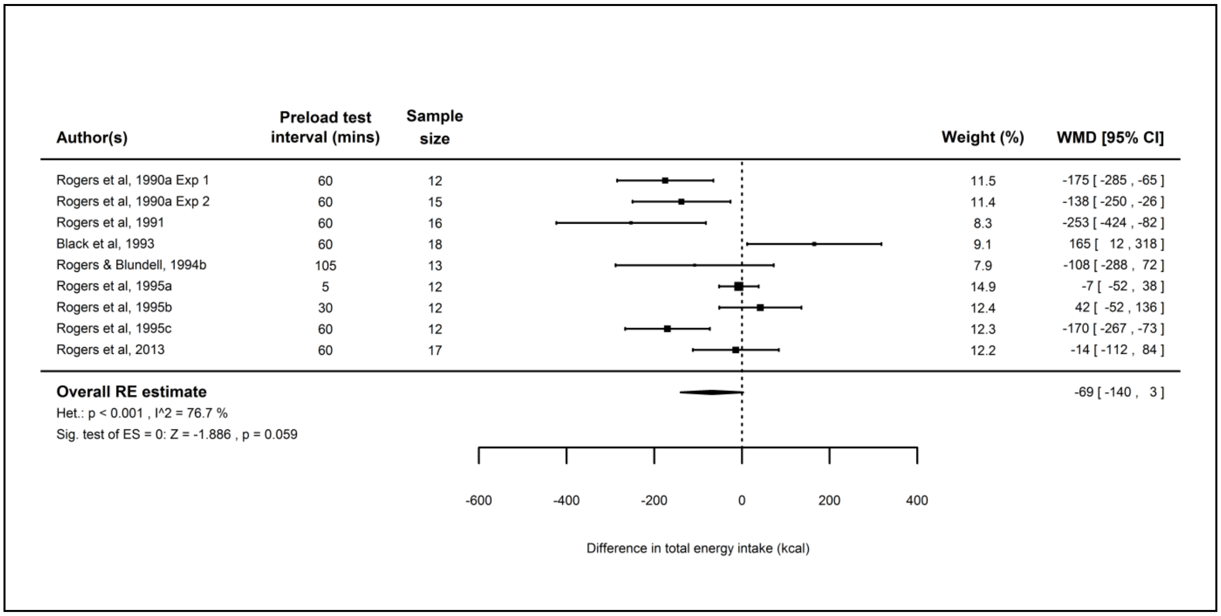

Figure S7. Forest plot of short-term intervention studies comparing the effects on EI of LES in capsules versus placebo capsules.

Difference in total EI is the difference in cumulative EI (preload plus test meal) for the LES condition minus the placebo condition. Squares represent mean difference in EI for the individual comparisons; square size is proportional to the weight of each comparison; horizontal lines represent 95% CIs; the filled diamond represents the summary estimates and 95% CIs from random effects models for all of the comparisons.

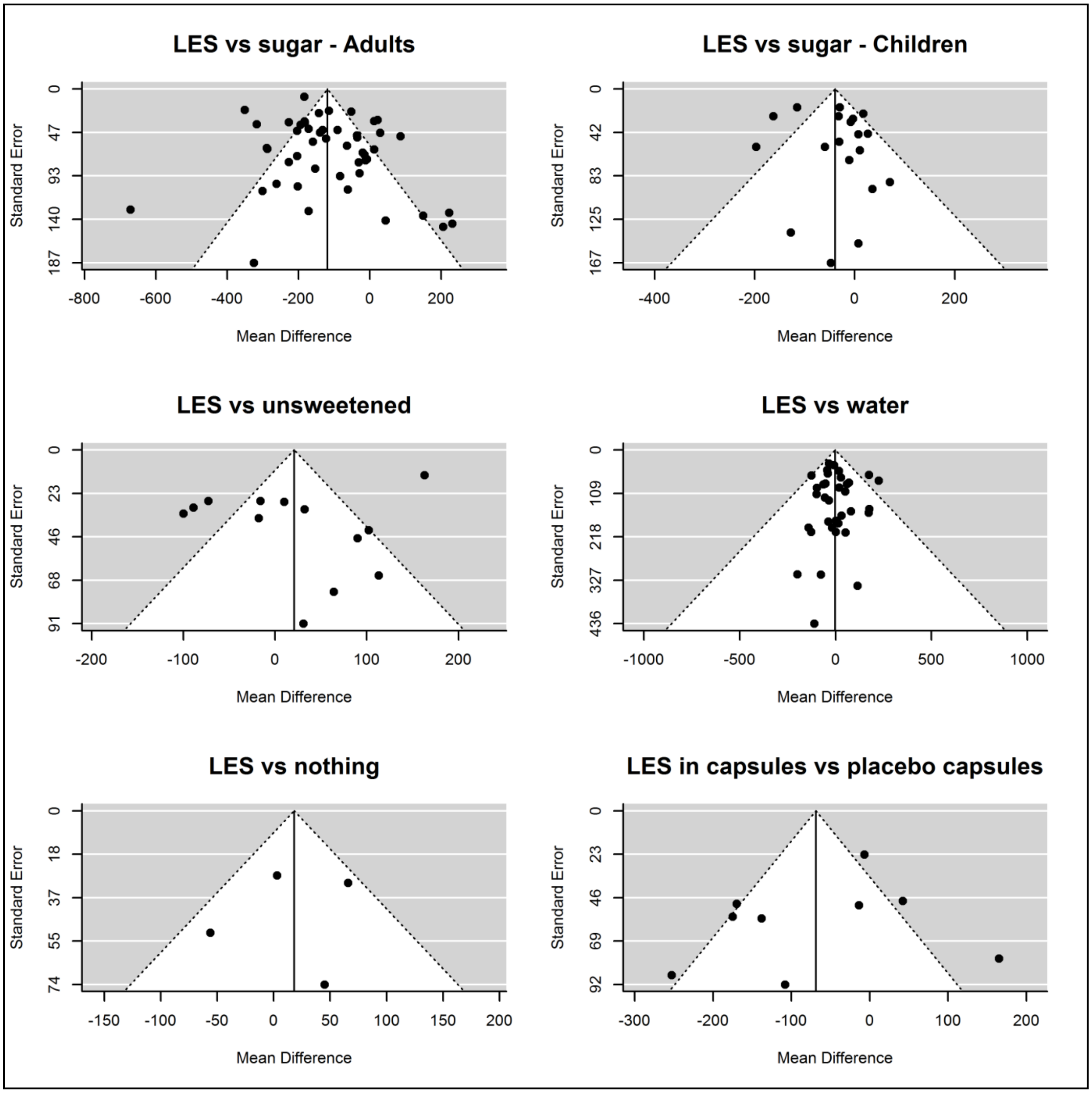

Figure S8. Funnel plots of short-term intervention studies comparing the effects on EI of LES versus sugar (for adults and children separately).

LES-sweetened versus unsweetened products, LES versus water, LES versus nothing and LES in capsules versus placebo capsules. Mean difference is the difference in cumulative EI (preload plus test meal) for the LES condition minus the comparison condition.

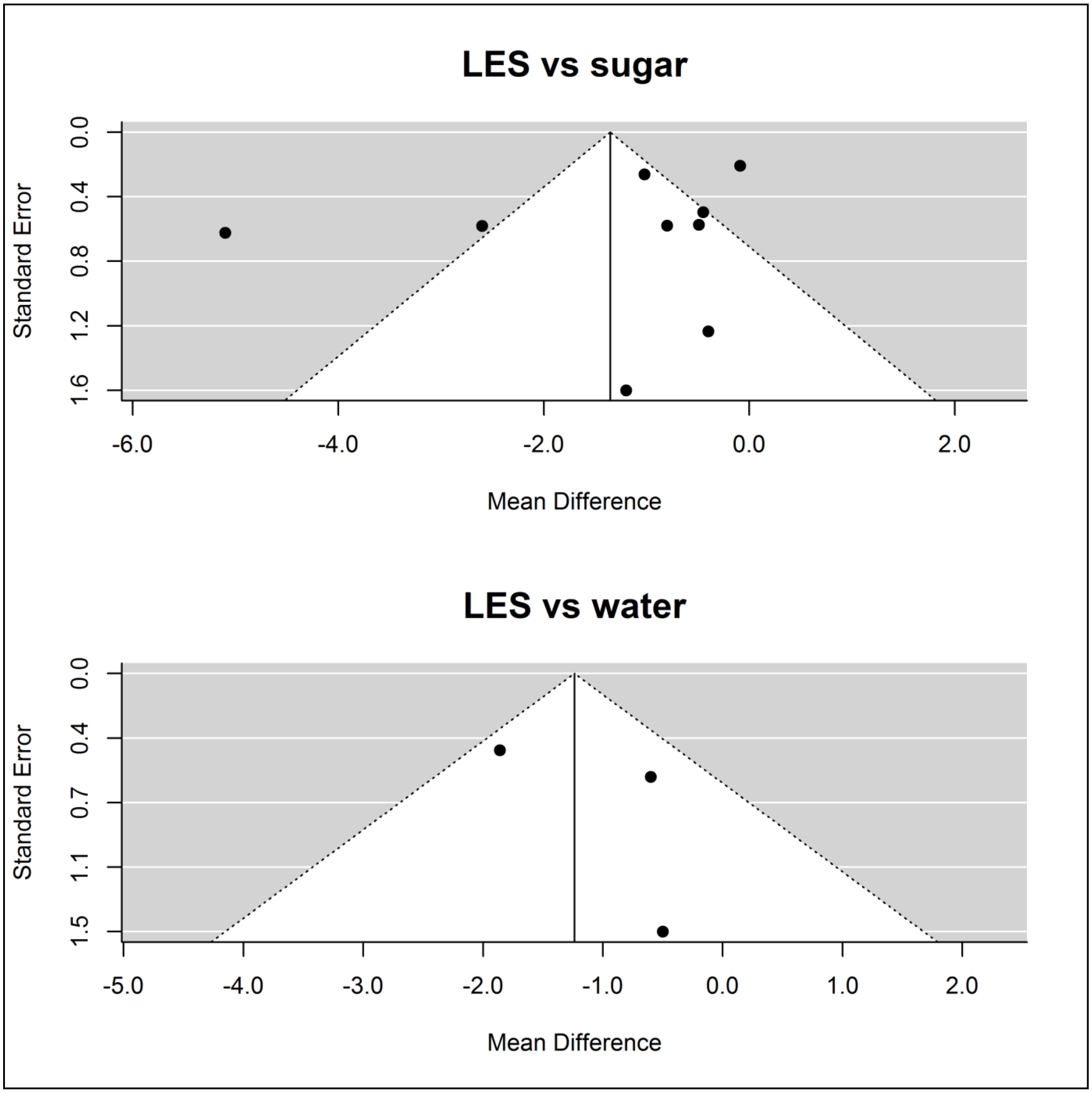

Figure S9. Funnel plots of sustained intervention studies comparing the effects on BW of LES versus sugar and LES versus water.

Mean difference is weight change in kg (end point minus baseline) in the LES condition minus weight change in the water condition over the intervention period.

Reference List

1. European Commission. European Parliament and the Council of the European Union. Corrigendum to Regulation EC)No 1924/2006 of the European Parliament and of the Council of 20 December 2006 on nutrition and health claims made on foods. *Off J Eur Union* 2006; **50**: 3-18.

2. Van Wymelbeke V, Beridot-Therond ME, de La Gueronniere V, Fantino M. Influence of repeated consumption of beverages containing sucrose or intense sweeteners on food intake. *Eur J Clin Nutr* 2004; **58**: 154-161.

3. Appleton KM, Blundell JE. Habitual high and low consumers of artificially-sweetened beverages: effects of sweet taste and energy on short-term appetite. *Physiol Behav* 2007; **92**: 479-486.

4. Beridot-Therond ME, Arts I, Fantino M, de La Gueronniere V. Short-term effects of the flavour of drinks on ingestive behaviours in man. *Appetite* 1998; **31**: 67-81.

5. Lavin JH, French SJ, Read NW. The effect of sucrose-and aspartame-sweetened drinks on energy intake, hunger and food choice of female, moderately restrained eaters. *Int J Obes Relat Metab Disord* 1997; **21**: 37-42.

6. Blackburn GL, Kanders BS, Lavin PT, Keller SD, Whatley J. The effect of aspartame as part of a multidisciplinary weight-control program on short-and long-term control of body weight. *Am J Clin Nutr* 1997; **65**: 409-418.

7. de Ruyter JC, Olthof MR, Seidell JC, Katan MB. A trial of sugar-free or sugar-sweetened beverages and body weight in children. *N Engl J Med* 2012; **367**: 1397-1406.

8. Kanders BS, Lavin PT, Kowalchuk MB, Greenberg I, Blackburn GL. An evaluation of the effect of aspartame on weight loss. *Appetite* 1988; **11**: 73-84.

9. Maersk M, Belza A, Stødkilde-Jørgensen H, Ringgaard S, Chabanova E, Thomsen H*, et al.* Sucrose-sweetened beverages increase fat storage in the liver, muscle, and visceral fat depot: a 6-mo randomized intervention study. *Am J Clin Nutr* 2012; **95**: 283-289.

10. Naismith DJ, Rhodes C. Adjustment in energy intake following the covert removal of sugar from the diet. *J Hum Nutr Diet* 1995; **8**: 167-175.

11. Njike VY, Faridi Z, Shuval K, Dutta S, Kay CD, West SG*, et al.* Effects of sugar-sweetened and sugar-free cocoa on endothelial function in overweight adults. *Int J Cardiol* 2011; **149**: 83-88.

12. Peters JC, Wyatt HR, Foster GD, Pan Z, Wojtanowski AC, Vander Veur SS*, et al.* The effects of water and non‐nutritive sweetened beverages on weight loss during a 12‐week weight loss treatment program. *Obesity* 2014; **22**: 1415-1421.

13. Raben A, Vasilaras TH, Müller AC, Astrup A. Sucrose compared with artificial sweeteners: different effects on ad libitum food intake and body weight after 10 wk of supplementation in overweight subjects. *Am J Clin Nutr* 2002; **76**: 721-729.

14. Reid M, Hammersley R, Hill AJ, Skidmore P. Long-term dietary compensation for added sugar: effects of supplementary sucrose drinks over a 4-week period. *Br J Nutr* 2007; **97**: 193-203.

15. Reid M, Hammersley R, Duffy M. Effects of sucrose drinks on macronutrient intake, body weight, and mood state in overweight women over 4 weeks. *Appetite* 2010; **55**: 130-136.

16. Tate DF, Turner-McGrievy G, Lyons E, Stevens J, Erickson K, Polzien K*, et al.* Replacing caloric beverages with water or diet beverages for weight loss in adults: main results of the Choose Healthy Options Consciously Everyday (CHOICE) randomized clinical trial. *Am J Clin Nutr* 2012; **95**: 555-563.

17. Tordoff MG, Alleva AM. Effect of drinking soda sweetened with aspartame or high-fructose corn syrup on food intake and body weight. *Am J Clin Nutr* 1990; **51**: 963-969.

18. Wolraich ML, Lindgren SD, Stumbo PJ, Stegink LD, Appelbaum MI, Kiritsy MC. Effects of diets high in sucrose or aspartame on the behavior and cognitive performance of children. *N Engl J Med* 1994; **330**: 301-307.

19. Higgins JPT, Green S. Cochrane handbook for systematic reviews of interventions Version 5.1. 0 [updated March 2011]. The Cochrane Collaboration, 2011. Available at: www cochrane-hbook org.

20. Fowler SP, Williams K, Resendez RG, Hunt KJ, Hazuda HP, Stern MP. Fueling the obesity epidemic? Artificially sweetened beverage use and long-term weight gain. *Obesity* 2008; **16**: 1894-1900.

21. Pan A, Malik VS, Hao T, Willett WC, Mozaffarian D, Hu FB. Changes in water and beverage intake and long-term weight changes: results from three prospective cohort studies. *Int J Obes* 2013; **37**: 1378-1385.

22. Striegel-Moore RH, Thompson D, Affenito SG, Franko DL, Obarzanek E, Barton BA*, et al.* Correlates of beverage intake in adolescent girls: the National Heart, Lung, and Blood Institute Growth and Health Study. *J Pediatr* 2006; **148**: 183-187.

23. Berkey CS, Rockett HR, Field AE, Gillman MW, Colditz GA. Sugar‐added beverages and adolescent weight change. *Obes Res* 2004; **12**: 778-788.

24. Laska MN, Murray DM, Lytle LA, Harnack LJ. Longitudinal associations between key dietary behaviors and weight gain over time: transitions through the adolescent years. *Obesity* 2012; **20**: 118-125.

25. Chen L, Appel LJ, Loria C, Lin PH, Champagne CM, Elmer PJ*, et al.* Reduction in consumption of sugar-sweetened beverages is associated with weight loss: the PREMIER trial. *Am J Clin Nutr* 2009; **89**: 1299-1306.

26. Duffey KJ, Steffen LM, Van Horn L, Jacobs DR, Popkin BM. Dietary patterns matter: diet beverages and cardiometabolic risks in the longitudinal Coronary Artery Risk Development in Young Adults (CARDIA) Study. *Am J Clin Nutr* 2012; **95**: 909-915.

27. Ludwig DS, Peterson KE, Gortmaker SL. Relation between consumption of sugar-sweetened drinks and childhood obesity: a prospective, observational analysis. *Lancet* 2001; **357**: 505-508.

28. Nettleton JA, Lutsey PL, Wang Y, Lima JA, Michos ED, Jacobs DR. Diet soda intake and risk of incident metabolic syndrome and type 2 diabetes in the Multi-Ethnic Study of Atherosclerosis (MESA). *Diabetes Care* 2009; **32**: 688-694.

29. Vanselow MS, Pereira MA, Neumark-Sztainer D, Raatz SK. Adolescent beverage habits and changes in weight over time: findings from Project EAT. *Am J Clin Nutr* 2009; **90**: 1489-1495.

30. Akhavan T, Anderson GH. Effects of glucose-to-fructose ratios in solutions on subjective satiety, food intake, and satiety hormones in young men. *Am J Clin Nutr* 2007; **86**: 1354-1363.

31. Hedges LV, Tipton E, Johnson MC. Robust variance estimation in meta-regression with dependent effect size estimates. *Res Synth Methods* 2010; **1**: 39-65.

32. Tanner□Smith EE, Tipton E. Robust variance estimation with dependent effect sizes: practical considerations including a software tutorial in Stata and SPSS. *Res Synth Methods* 2014; **5**: 13-30.

33. Dalderup LM, Visser W. Effects of sodium cyclamate on the growth of rats compared with other variations in the diet. *Nature* 1969; **221**.

34. Dalderup LM, Visser W. Influence of extra sucrose, fats, protein and of cyclamate in the daily food on the life-span of rats. *Experientia* 1971; **27**: 519-521.

35. Friedhoff R, Simon JA, Friedhoff AJ. Sucrose solution vs. no-calorie sweetener vs. water in weight gain. *J Am Diet Assoc* 1971; **59**: 485-486.

36. Brantom PG, Gaunt IF, Grasso P. Long-term toxicity of sodium cyclamate in mice. *Food Cosmet Toxicol* 1973; **11**: 735-746.

37. Munro IC, Moodie CA, Krewski D, Grice HC. A carcinogenicity study of commercial saccharin in the rat. *Toxicol Appl Pharmacol* 1975; **32**: 513-526.

38. Oser BL, Carson S, Cox GE, Vogin EE, Sternberg SS. Chronic toxicity study of cyclamate: saccharin (10: 1) in rats. *Toxicology* 1975; **4**: 385-386.

39. Anderson RL. Response of male rats to sodium saccharin ingestion: urine composition and mineral balance. *Food Cosmet Toxicol* 1979; **17**: 195-200.

40. Watkins AL, Williams L, Carroll C. Influence of saccharin on growth and tissue lipids in rats fed different fats and carbohydrates. *Nutr Rep Int* 1980; **21**: 329-339.

41. Ishii H, Koshimizu T, Usami S, Fujimoto T. Toxicity of aspartame and its diketopiperazine for Wistar rats by dietary administration for 104 weeks. *Toxicology* 1981; **21**: 91-94.

42. Higginbotham JD, Snodin DJ, Eaton KK, Daniel JW. Safety evaluation of thaumatin (talin protein). *Food Chem Toxicol* 1983; **21**: 815-823.

43. Schoenig GP, Goldenthal EI, Geil RG, Frith CH, Richter WR, Carlborg FW. Evaluation of the dose response and in utero exposure to saccharin in the rat. *Food Chem Toxicol* 1985; **23**: 475-490.

44. Fisher MJ, Sakata T, Tibbels TS, Smith RA, Patil K, Khachab M*, et al.* Effect of sodium saccharin and calcium saccharin on urinary parameters in rats fed Prolab 3200 or AIN-76 diet. *Food Chem Toxicol* 1989; **27**: 1-9.

45. Lina BAR, Dreef-van der Meulen H, Leegwater DC. Subchronic (13-week) oral toxicity of neohesperidin dihydrochalcone in rats. *Food Chem Toxicol* 1990; **28**: 507-513.

46. Xili L, Chengjiany B, Eryi X, Reiming S, Yuengming W, Haodong S*, et al.* Chronic oral toxicity and carcinogenicity study of stevioside in rats. *Food Chem Toxicol* 1992; **30**: 957-965.

47. Lina BAR, Bos-Kuijpers MHM, Til HP, Bär A. Chronic toxicity and carcinogenicity study of erythritol in rats. *Regul Toxicol Pharmacol* 1996; **24**: S264-S279.

48. Til HP, Modderman J. Four-week oral toxicity study with erythritol in rats. *Regul Toxicol Pharmacol* 1996; **24**: S214-S220.

49. Til HP, Kuper CF, Falke HE, Bä A. Subchronic oral toxicity studies with erythritol in mice and rats. *Regul Toxicol Pharmacol* 1996; **24**: S221-S231.

50. Bailey CJ, Day C, Knapper JM, Turner SL, Flatt PR. Antihyperglycaemic effect of saccharin in diabetic ob/ob mice. *Br J Pharmacol* 1997; **120**: 74-78.

51. Goldsmith LA. Acute and subchronic toxicity of sucralose. *Food Chem Toxicol* 2000; **38**: 53-69.

52. Beck B, Burlet A, Max JP, Stricker-Krongrad A. Effects of long-term ingestion of aspartame on hypothalamic neuropeptide Y, plasma leptin and body weight gain and composition. *Physiol Behav* 2002; **75**: 41-47.

53. Jeppesen PB, Gregersen S, Rolfsen SED, Jepsen M, Colombo M, Agger A*, et al.* Antihyperglycemic and blood pressure-reducing effects of stevioside in the diabetic Goto-Kakizaki rat. *Metabolism* 2003; **52**: 372-378.

54. Waalkens-Berendsen DH, Kuilman-Wahls MEM, Bär A. Embryotoxicity and teratogenicity study with neohesperidin dihydrochalcone in rats. *Regul Toxicol Pharmacol* 2004; **40**: 74-79.

55. Jürgens H, Haass W, Castaneda TR, Schürmann A, Koebnick C, Dombrowski F*, et al.* Consuming fructose‐sweetened beverages increases body adiposity in mice. *Obes Res* 2005; **13**: 1146-1156.

56. Dyrskog SEU, Jeppesen PB, Colombo M, Abudula R, Hermansen K. Preventive effects of a soy-based diet supplemented with stevioside on the development of the metabolic syndrome and type 2 diabetes in Zucker diabetic fatty rats. *Metabolism* 2005; **54**: 1181-1188.

57. Tago K, Naito Y, Nagata T, Morimura T, Furuya M, Seki T*, et al.* A ninety-day feeding, subchronic toxicity study of oligo-N-acetylglucosamine in Fischer 344 rats. *Food Chem Toxicol* 2007; **45**: 1186-1193.

58. Bergheim I, Weber S, Vos M, Krämer S, Volynets V, Kaserouni S*, et al.* Antibiotics protect against fructose-induced hepatic lipid accumulation in mice: role of endotoxin. *J Hepatol* 2008; **48**: 983-992.

59. Curry LL, Roberts A. Subchronic toxicity of rebaudioside A. *Food Chem Toxicol* 2008; **46**: S11-S20.

60. Nikiforov AI, Eapen AK. A 90-day oral (dietary) toxicity study of rebaudioside A in Sprague-Dawley rats. *Int J Toxicol* 2008; **27**: 65-80.

61. Figlewicz DP, Ioannou G, Bennett Jay J, Kittleson S, Savard C, Roth CL. Effect of moderate intake of sweeteners on metabolic health in the rat. *Physiol Behav* 2009; **98**: 618-624.

62. Yagi K, Matsuo T. The study on long-term toxicity of D-psicose in rats. *J Clin Biochem Nutr* 2009; **45**: 271-277.

63. Park J, Cha Y-S. Stevia rebaudiana Bertoni extract supplementation improves lipid and carnitine profiles in C57BL/6J mice fed a high‐fat diet. *J Sci Food Agriculture* 2010; **90**: 1099-1105.

64. Park JS, Yoo SB, Kim JY, Lee SJ, Oh SB, Kim JS*, et al.* Effects of saccharin intake on hippocampal and cortical plasticity in juvenile and adolescent rats. *Korean J Physiol Pharmacol* 2010; **14**: 113-118.

65. Geeraert B, Crombe F, Hulsmans M, Benhabiles N, Geuns JM, Holvoet P. Stevioside inhibits atherosclerosis by improving insulin signaling and antioxidant defense in obese insulin-resistant mice. *Int J Obes* 2009; **34**: 569-577.

66. Andrejic B, Mijatovic V, Calasan J, Horvat O, Samojlik I. Influence of Saccharin on Changes in Rat Pancreas and Liver Histology, Glycaemia, Food Intake and Weight. *J Comp Pathol* 2010; **143**: 344.

67. Polyák E, Gombos K, Hajnal B, Bonyár-Müller K, Szabó S, Gubicskó-Kisbenedek A*, et al.* Effects of artificial sweeteners on body weight, food and drink intake. *Acta Physiol Hung* 2010; **97**: 401-407.

68. Hlywka J, Brathwaite WA, Rihner MO, Nikiforov AI, Eapen AK. A 90-day oral (dietary) toxicity study of the 2R,4R-isomer of monatin salt in Sprague�Dawley rats. *Food ChemToxicol* 2011; **49**: 3249-3257.

69. Otabe A, Fujieda T, Masuyama T. Chronic toxicity and carcinogenicity of N-N-[3-(3-hydroxy-4-methoxyphenyl) propyl]-alpha-aspartyl]-l-phenylalanine 1-methyl ester, monohydrate (advantame) in the rat. *Food Chem Toxicol* 2011; **49**: S35-S48.

70. Otabe A, Fujieda T, Masuyama T. A two-generation reproductive toxicity study of the high-intensity sweetener advantame in CD rats. *Food Chem Toxicol* 2011; **49**: S70-S76.

71. Reis TA, Goulart P, de Oliveira RME, Oliveira Ld, de Abreu PS, Azevedo Ad. Metabolic parameters of wistar rats subjected to diet supplemented with stevia and sugar. *Semina: Ciéncias Agrarias (Londrina)* 2011; **32**: 1477-1488.

72. Brathwaite WA, Casterton PL, Nikiforov AI, Rihner MO, Sloter ED, Hlywka JJ. A dietary embryo/fetal developmental toxicity study of arruva, an R,R-monatin salt isomer, in Crl: CD (SD) rats. *Food Chem Toxicol* 2013; **62**: 68-75.

73. Nikiforov AI, Rihner MO, Eapen AK, Thomas JA. Metabolism and Toxicity Studies Supporting the Safety of Rebaudioside D. *Int J Toxicol* 2013; **32**: 261-273.

74. Mitsutomi K, Masaki T, Shimasaki T, Gotoh K, Chiba S, Kakuma T*, et al.* Effects of a nonnutritive sweetener on body adiposity and energy metabolism in mice with diet-induced obesity. *Metabolism* 2014; **63**: 69-78.

75. Fulop I, Bobes D, Croitoru D, Barbu C. influence of sugars and sweeteners on mouse body weight, stress-induced body weight changes and life expectancy. *Farmacia* 2014; **62**: 376-389.

76. Abu-Taweel GM, Zyadah MA, Ajarem JS, Ahmad M. Cognitive and biochemical effects of monosodium glutamate and aspartame, administered individually and in combination in male albino mice. *Neurotoxicol Teratol* 2014; **42**: 60-67.

77. Palmnas MS, Cowan TE, Bomhof MR, Su J, Reimer RA, Vogel HJ*, et al.* Low-dose aspartame consumption differentially affects gut microbiota-host metabolic interactions in the diet-induced obese rat. *PloS one* 2014; **9**: e109841.

78. Porikos KP, Koopmans HS. The effect of non-nutritive sweeteners on body weight in rats. *Appetite* 1988; **11**: 12-15.

79. Ramirez I. Stimulation of energy intake and growth by saccharin in rats. *J Nutr* 1990; **120**: 123-133.

80. Kanarek RB, White ES, Biegen MT, Marks-Kaufman R. Dietary influences on morphine-induced analgesia in rats. *Pharmacol Biochem Behav* 1991; **38**: 681-684.

81. D'Anci KE, Kanarek RB, Marks-Kaufman R. Beyond sweet taste: saccharin, sucrose, and polycose differ in their effects upon morphine-induced analgesia. *Pharmacol Biochem Behav* 1997; **56**: 341-345.

82. Kanarek RB, Przypek J, D'Anci KE, Marks-Kaufman R. Dietary modulation of mu and kappa opioid receptor-mediated analgesia. *Pharmacol Biochem Behav* 1997; **58**: 43-49.

83. Kanarek RB, Mathes WF, Heisler LK, Lima RP, Monfared LS. Prior exposure to palatable solutions enhances the effects of naltrexone on food intake in rats. *Pharmacol Biochem Behav* 1997; **57**: 377-381.

84. Yeomans MR, Clifton PG. Exposure to Sweetened Solutions Enhances the Anorectic Effect of Naloxone But Not d-Fenfluramine. *Physiol Behav* 1997; **62**: 255-262.

85. D'Anci KE. Tolerance to morphine-induced antinociception is decreased by chronic sucrose or polycose intake. *Pharmacol Biochem Behav* 1999; **63**: 1-11.

86. Kanarek RB, Homoleski B. Modulation of morphine-induced antinociception by palatable solutions in male and female rats. *Pharmacol Biochem Behav* 2000; **66**: 653-659.

87. Feijó FdM, Ballard CR, Foletto KC, Batista BAM, Neves AM, Ribeiro MFM*, et al.* Saccharin and aspartame, compared with sucrose, induce greater weight gain in adult Wistar rats, at similar total caloric intake levels. *Appetite* 2013; **60**: 203-207.

88. Swithers SE, Davidson TL. A role for sweet taste: calorie predictive relations in energy regulation by rats. *Behav Neurosci* 2008; **122**: 161-173.

89. Swithers SE, Baker CR, Davidson TL. General and persistent effects of high-intensity sweeteners on body weight gain and caloric compensation in rats. *Behav Neurosci* 2009; **123**: 772-780.

90. Swithers SE, Martin AA, Clark KM, Laboy AF, Davidson TL. Body weight gain in rats consuming sweetened liquids. Effects of caffeine and diet composition. *Appetite* 2010; **55**: 528-533.

91. Davidson TL, Martin AA, Clark K, Swithers SE. Intake of high-intensity sweeteners alters the ability of sweet taste to signal caloric consequences: implications for the learned control of energy and body weight regulation. *Q J Exp Psychol* 2011; **64**: 1430-1441.

92. Swithers SE, Laboy AF, Clark K, Cooper S, Davidson TL. Experience with the high-intensity sweetener saccharin impairs glucose homeostasis and GLP-1 release in rats. *Behav Brain Res* 2012; **233**: 1-14.

93. Swithers SE. Artificial sweeteners produce the counterintuitive effect of inducing metabolic derangements. *Trends Endocrinol Metab* 2013; **24**: 431-441.

94. Swithers SE, Sample CH, Katz DP. Influence of ovarian and non-ovarian estrogens on weight gain in response to disruption of sweet taste–calorie relations in female rats. *Horm Behav* 2013; **63**: 40-48.

95. Anderson GH, Saravis S, Schacher R, Zlotkin S, Leiter LA. Aspartame: effect on lunch-time food intake, appetite and hedonic response in children. *Appetite* 1989; **13**: 93-103.

96. Birch LL, McPhee L, Sullivan S. Children's food intake following drinks sweetened with sucrose or aspartame: time course effects. *Physiol Behav* 1989; **45**: 387-395.

97. Gheller B, Akhavan T, Pollard D, Gladanac B, Constantino M, Luhovyy B*, et al.* A pre-meal glucose drink, but not video game playing, suppresses food intake in overweight and obese boys (1040.8). *FASEB J* 2014; **28**: 1040-1048.

98. Branton A, Akhavan T, Gladanac B, Pollard D, Welch J, Rossiter M*, et al.* Pre-meal video game playing and a glucose preload suppress food intake in normal weight boys. *Appetite* 2014; **83**: 256-262.

99. Van Engelen M, Khodabandeh S, Akhavan T, Agarwal J, Gladanac B, Bellissimo N. Effect of sugars in solutions on subjective appetite and short-term food intake in 9-to 14-year-old normal weight boys. *Eur J Clin Nutr* 2014; **68**: 773-777.

100. Hetherington MM, Wood C, Lyburn SC. Response to energy dilution in the short term: evidence of nutritional wisdom in young children? *Nutr Neurosci* 2000; **3**: 321-329.

101. Wilson JF. Lunch eating behavior of preschool children: effects of age, gender, and type of beverage served. *Physi Behav* 2000; **70**: 27-33.

102. Bellissimo N, Pencharz PB, Thomas SG, Anderson GH. Effect of television viewing at mealtime on food intake after a glucose preload in boys. *Pediatr Res* 2007; **61**: 745-749.

103. Bellissimo N, Thomas SG, Goode RC, Anderson GH. Effect of short-duration physical activity and ventilation threshold on subjective appetite and short-term energy intake in boys. *Appetite* 2007; **49**: 644-651.

104. Patel BP, Bellissimo N, Thomas SG, Hamilton JK, Anderson GH. Television viewing at mealtime reduces caloric compensation in peripubertal, but not postpubertal, girls. *Pediatr Res* 2011; **70**: 513-517.

105. Tamam S, Bellissimo N, Patel BP, Thomas SG, Anderson GH. Overweight and obese boys reduce food intake in response to a glucose drink but fail to increase intake in response to exercise of short duration. *Appl Physiol Nutr Metab* 2012; **37**: 520-529.

106. Booth DCA, Chase AILE. Temporal bounds of post-ingestive glucose induced satiety in man. *Nature* 1970; **228**: 1104-1105.

107. Brala PM, Hagen RL. Effects of sweetness perception and caloric value of a preload on short term intake. *Physiol Behav* 1983; **30**: 1-9.

108. Rogers PJ, Carlyle JA, Hill AJ, Blundell JE. Uncoupling sweet taste and calories: comparison of the effects of glucose and three intense sweeteners on hunger and food intake. *Physiol Behav* 1988; **43**: 547-552.

109. Rogers PJ, Blundell JE. Separating the actions of sweetness and calories: effects of saccharin and carbohydrates on hunger and food intake in human subjects. *Physiol Behav* 1989; **45**: 1093-1099.

110. Rolls BJ, Laster LJ, Summerfelt A. Hunger and food intake following consumption of low-calorie foods. *Appetite* 1989; **13**: 115-127.

111. Rodin J. Comparative effects of fructose, aspartame, glucose, and water preloads on calorie and macronutrient intake. *Am J Clin Nutr* 1990; **51**: 428-435.

112. Rogers PJ, Lambert TC, Alikanizadeh LA, Blundell JE. Intense sweeteners and appetite: responses of informed and uninformed subjects consuming food sweetened with aspartame or sugar. *Int J Obes* 1990; **14**: 105.

113. Rolls BJ, Kim S, Fedoroff IC. Effects of drinks sweetened with sucrose or aspartame on hunger, thirst and food intake in men. *Physiol Behav* 1990; **48**: 19-26.

114. Canty DJ, Chan MM. Effects of consumption of caloric vs noncaloric sweet drinks on indices of hunger and food consumption in normal adults. *Am J Clin Nutr* 1991; **53**: 1159-1164.

115. Drewnowski A, Massien C, Louis-Sylvestre J, Fricker J, Chapelot D, Apfelbaum M. The effects of aspartame versus sucrose on motivational ratings, taste preferences, and energy intakes in obese and lean women. *Int J Obes Relat Metab Disord* 1994; **18**: 570-578.

116. Drewnowski A, Massien C, Louis-Sylvestre J, Fricker J, Chapelot D, Apfelbaum M. Comparing the effects of aspartame and sucrose on motivational ratings, taste preferences, and energy intakes in humans. *Am J Clin Nutr* 1994; **59**: 338-345.

117. Guss JL, Kissileff HR, Pi-Sunyer FX. Effects of glucose and fructose solutions on food intake and gastric emptying in nonobese women. *Am J Physi-Regul Integr Comp Physiol* 1994; **36**: R1537.

118. Reid M, Hammersley R. Effects of carbohydrate intake on subsequent food intake and mood state. *Physiol Behav* 1995; **58**: 421-427.

119. Kim JY, Kissileff HR. The effect of social setting on response to a preloading manipulation in non-obese women and men. *Appetite* 1996; **27**: 25-40.

120. King NA, Appleton K, Rogers PJ, Blundell JE. Effects of sweetness and energy in drinks on food intake following exercise. *Physiol Behav* 1999; **66**: 375-379.

121. Melanson KJ, Westerterp-Plantenga MS, Campfield AL, Saris WH. Blood glucose and meal patterns in time-blinded males, after aspartame, carbohydrate, and fat consumption, in relation to sweetness perception. *Br J Nutr* 1999; **82**: 437-446.

122. Reid M, Hammersley R. The effects of sucrose and maize oil on subsequent food intake and mood. *Br J Nutr* 1999; **82**: 447-455.

123. Holt NS. The effects of sugar-free vs sugar-rich beverages on feelings of fullness and subsequent food intake. *Int J Food Sci Nutr* 2000; **51**: 59-71.

124. Woodend DM, Anderson GH. Effect of sucrose and safflower oil preloads on short term appetite and food intake of young men. *Appetite* 2001; **37**: 185-195.

125. DellaValle DM, Roe LS, Rolls BJ. Does the consumption of caloric and non-caloric beverages with a meal affect energy intake? *Appetite* 2005; **44**: 187-193.

126. Monsivais P, Perrigue MM, Drewnowski A. Sugars and satiety: does the type of sweetener make a difference? *Am J Clin Nutr* 2007; **86**: 116-123.

127. Soenen S, Westerterp-Plantenga MS. No differences in satiety or energy intake after high-fructose corn syrup, sucrose, or milk preloads. *Am J Clin Nutr* 2007; **86**: 1586-1594.

128. Anton SD, Martin CK, Han H, Coulon S, Cefalu WT, Geiselman P*, et al.* Effects of stevia, aspartame, and sucrose on food intake, satiety, and postprandial glucose and insulin levels. *Appetite* 2010; **55**: 37-43.

129. Ranawana DV, Henry CJK. Are caloric beverages compensated for in the short-term by young adults? An investigation with particular focus on gender differences. *Appetite* 2010; **55**: 137-146.

130. Akhavan T, Luhovyy BL, Anderson GH. Effect of drinking compared with eating sugars or whey protein on short-term appetite and food intake. *Int J Obesity* 2010; **35**: 562-569.

131. Rogers PJ, Gadah NS, Kyle LA, Brunstrom JM. Standard preload-test meal study designs may underestimate satiety effects: sugar containing drinks as an example. *Proc Nutr Soc* 2011; **70**: E401.

132. Maersk M, Belza A, Holst JJ, Fenger-Grøn M, Pedersen SB, Astrup A*, et al.* Satiety scores and satiety hormone response after sucrose-sweetened soft drink compared with isocaloric semi-skimmed milk and with non-caloric soft drink: a controlled trial. *Eur J Clin Nutr* 2012; **66**: 523-529.

133. Carvalho P, Sousa M, Barros R, Padrao P, Moreira P, Teixeira V. Impact of morning ingestion of sugary and sweetened beverages on energy and fluid intake throughout day. *Ann Nutr Metab* 2013; **63**: 1448-1449.

134. Panahi S, El Khoury D, Luhovyy BL, Goff HD, Anderson GH. Caloric beverages consumed freely at meal-time add calories to an ad libitum meal. *Appetite* 2013; **65**: 75-82.

135. Ho EE, Liszt AF, Pudel V. The effects of energy content and sweet taste on food consumption in restrained and non-restrained eaters. *J Am Diet Assoc* 1990; **90**: 1223-8

136. Rogers PJ. Why a palatability construct is needed. *Appetite* 1990; **14**: 167-170.

137. Black RM, Tanaka P, Leiter LA, Anderson GH. Soft drinks with aspartame: effect on subjective hunger, food selection, and food intake of young adult males. *Physiol Behav* 1991; **49**: 803-810.

138. Black RM, Leiter LA, Anderson GH. Consuming aspartame with and without taste: differential effects on appetite and food intake of young adult males. *Physiol Behav* 1993; **53**: 459-466.

139. Ford HE, Peters V, Martin NM, Sleeth ML, Ghatei MA, Frost GS*, et al.* Effects of oral ingestion of sucralose on gut hormone response and appetite in healthy normal-weight subjects. *Eur J Clin Nutr* 2011; **65**: 508-513.

140. Rogers PJ, Blundell JE. Reanalysis of the effects of phenylalanine, alanine, and aspartame on food intake in human subjects. *Physiol Behav* 1994; **56**: 247-250.

141. Rogers PJ, Burley VJ, Alikhanizadeh LA, Blundell JE. Postingestive inhibition of food intake by aspartame: importance of interval between aspartame administration and subsequent eating. *Physiol Behav* 1995; **57**: 489-493.

142. Rogers PJ. What is the mechanism for the postingestive anorectic effect of aspartame? *Appetite* 2013; **71**: 485.

143. Raben A, Richelsen B. Artificial sweeteners: a place in the field of functional foods? Focus on obesity and related metabolic disorders. *Curr Opin Clin Nutr Metab Care* 2012; **15**: 597-604.

144. Miller P, Perez V. Low-calorie sweeteners and body weight and composition: a meta-analysis of randomized controlled trials and prospective cohorts (391.1). *FASEB J* 2014; **28**: 391.

145. Colditz GA, Willett WC, Stampfer MJ, London SJ, Segal MR, Speizer FE. Patterns of weight change and their relation to diet in a cohort of healthy women. *Am J Clin Nutr* 1990; **51**: 1100-1105.

146. Johnson L, Mander AP, Jones LR, Emmett PM, Jebb SA. Is sugar-sweetened beverage consumption associated with increased fatness in children? *Nutrition* 2007; **23**: 557-563.

147. Newby PK, Tucker KL. Empirically derived eating patterns using factor or cluster analysis: a review. *Nutr Rev* 2004; **62**: 177-203.

148. Parker DR, Gonzalez S, Derby CA, Gans KM, Lasater TM, Carleton RA. Dietary factors in relation to weight change among men and women from two southeastern New England communities. *Int J Obes Relat Metab Disord* 1997; **21**: 103-109.

149. Schulze MB, Manson JE, Ludwig DS, Colditz GA, Stampfer MJ, Willett WC*, et al.* Sugar-sweetened beverages, weight gain, and incidence of type 2 diabetes in young and middle-aged women. *JAMA* 2004; **292**: 927-934.

150. Knopp RH, Brandt K, Arky RA. Effects of aspartame in young persons during weight reduction. *J Toxic Environ Health A* 1976; **2**: 417-428.

151. Gatenby SJ, Aaron JI, Jack VA, Mela DJ. Extended use of foods modified in fat and sugar content: nutritional implications in a free-living female population. *Am J Clin Nutr* 1997; **65**: 1867-1873.

152. Gostner A, Schäffer V, Theis S, Menzel T, Lührs H, Melcher R*, et al.* Effects of isomalt consumption on gastrointestinal and metabolic parameters in healthy volunteers. *Br J Nutr* 2005; **94**: 575-581.

153. Ebbeling CB, Feldman HA, Osganian SK, Chomitz VR, Ellenbogen SJ, Ludwig DS. Effects of decreasing sugar-sweetened beverage consumption on body weight in adolescents: a randomized, controlled pilot study. *Pediatrics* 2006; **117**: 673-680.

154. Ebbeling CB, Feldman HA, Chomitz VR, Antonelli TA, Gortmaker SL, Osganian SK*, et al.* A randomized trial of sugar-sweetened beverages and adolescent body weight. *N Engl J Med* 2012; **367**: 1407-1416.
